# Interplay Between Residential Nature Exposure and Walkability and Their Association with Cardiovascular Health

**DOI:** 10.1016/j.jacadv.2024.101457

**Published:** 2024-12-17

**Authors:** Omar M. Makram, Nwabunie Nwana, Alan Pan, Juan C. Nicolas, Rakesh Gullapelli, Budhaditya Bose, Ashutosh Sabharwal, Jenny Chang, Zulqarnain Javed, Bita Kash, Jay E. Maddock, Khurram Nasir, Sadeer Al-Kindi

**Affiliations:** a Center for Health & Nature, Houston Methodist Research Institute, Houston, Texas, USA; b Department of Medicine, Medical College of Georgia at Augusta University, Augusta, Georgia, USA; c Center for Health Data Science and Analytics, Houston Methodist Research Institute, Houston, Texas, USA; d Department of Electrical and Computer Engineering, Rice University, Houston, Texas, USA; e Houston Methodist Neal Cancer Center, Houston Methodist Hospital, Houston, Texas, USA; f Department of Environmental and Occupational Health, School of Public Health, Texas A&M University, College Station, Texas, USA; g Division of Cardiovascular Prevention and Wellness, Department of Cardiology, Houston Methodist DeBakey Heart and Vascular Center, Houston, Texas, USA; h Center for Cardiovascular Computational Health & Precision Medicine (C3-PH), Houston Methodist Hospital, Houston, Texas, USA

**Keywords:** cardiovascular, diabetes, hypertension, nature, walkability

## Abstract

**Background:**

Green space has been linked with cardiovascular (CV) health. Nature access and quality may have significant impact on CV risk factors and health.

**Objectives:**

The authors aimed to investigate the relationship between NatureScore, a composite score for natural environment exposure and quality of green spaces, with CV risk factors and atherosclerotic cardiovascular diseases (ASCVD).

**Methods:**

A cross-sectional study including one million adult patients from the Houston Methodist Learning Health System Outpatient Registry (2016-2022). NatureScore is a composite measure of natural environment exposure and quality (0-100) calculated for each patient based on residential address. NatureScores was divided into 4 categories: nature deficient/light (0-39), nature adequate (40-59), nature rich (60-79), and nature utopia (80-100). CV risk factors included hypertension, diabetes, dyslipidemia, and obesity.

**Results:**

Among 1.07 million included patients (mean age 52 years, female 59%, Hispanic 16%, Non-Hispanic Black 14%), median NatureScore was 69.4. After adjusting for neighborhood walkability, patients living in highest NatureScore neighborhoods had lower prevalence of CV risk factors (OR: 0.91, 95% CI: 0.90-0.93) and ASCVD (OR: 0.96, 95% CI: 0.93-0.98) than those in lowest NatureScore neighborhoods. A significant interaction existed between NatureScore and Walkability (*P* < 0.001), where those in high NatureScore (≥60) high walkability (≥40) areas had lower prevalence of CV risk factors (OR: 0.93, 95% CI: 0.90-0.97, *P* < 0.001) and were more likely to have optimal CV risk profile (relative risk ratio: 1.09, 95% CI: 1.04-1.14, *P* = 0.001).

**Conclusions:**

These findings suggest that while green spaces benefit health, their accessibility through walkable environments is crucial for cardiovascular disease protection.

Environmental, occupational, behavioral, and metabolic risk factors contribute around 50% of the global burden of disease and disability.^1^ Among these factors, neighborhood characteristics and built environment, including exposure to green spaces and natural environments, have been extensively studied for their association with cardiovascular (CV) health.^2^, ^3^, ^4^, ^5^ This association was frequently explained through the impact of nature on stress reduction,^6^^,^^7^ physical activity promotion,^8^ and mitigation of climate change and pollution impacts.^9^

However, most studies have relied on population-level data or focused on a single metric without considering the complex interplay with other neighborhood characteristics. Notably, the combined impact of neighborhood walkability and greenness exposure on cardiovascular health remains underexplored. In our previous study, we found that adults residing in highly walkable neighborhoods had significantly lower CV risk factors.^10^

The present study aims to investigate the relationship between residential greenness exposure using NatureScore and the prevalence of CV risk factors and atherosclerotic cardiovascular diseases (ASCVD). Furthermore, we sought to elucidate the potential modifying effect of neighborhood walkability on this relationship.

## Methods

### Study setting and design

This cross-sectional study utilized data from the Houston Methodist Learning Health System Outpatient Registry (HM registry) from Houston Methodist Hospital system, which is a multicenter hospital with 8 locations all over Houston. The registry included adult individuals over the age of 18 who either presented or were transferred to one of the hospital with at least one outpatient encounter starting June 2016; de-identified patient health information were retrieved from the electronic health records (EHRs) and it included demographics, vitals, laboratory and imaging tests and results, medications, diagnoses, comorbid conditions, visit discharge diagnoses, International Classification of Diseases-10 Revision-Clinical Modification (ICD-10-CM), and the Current Procedural Terminology (CPT) codes. Further extensive analysis and discussion of the methodology and the framework used to integrate all the patients’ EHR with other data, such as SDOH, is currently available through recently published paper by our team.^11^ The methodology used for data retrieval, calculation of WalkScore, and dataset linkage was comprehensively described in an earlier study conducted by our team.^10^ The Houston Methodist institutional review board (IRB) office granted a waiver to both patient consent and Health Insurance Portability and Accountability Act (HIPAA) authorization (IRB ID PRO00025790). The study findings were reported following the Strengthening the Reporting of Observational Studies in Epidemiology (STROBE) guidelines (Supplemental Table 1).^12^

### Study population

Our population comprised adults, aged 18 years or older who had at least one outpatient encounter within the Houston Methodist Healthcare System between June 1st, 2016 to May 6th, 2022 (see Supplemental Table 2 for encounter types). Each individual was assigned a NatureScore based on the latest residential address recorded in the EHR after geocoding. The final analytical sample included 1,077,181 individuals.

### Study variables

#### Independent variable: NatureScore

NatureScore™ is a technology system that measures both the quantity and quality of nature in a given area by incorporating various data sources including land classification data, satellite images to assess both quantity and quality of green areas, noise and air pollution, and tree canopy coverage. This weights the information provided by each source and create an overall score for each given area, thus providing a more comprehensive measure for nature exposure, rather than the frequently used single-measure indices, such as the Normalized Difference Vegetation Index (NDVI).^13^^,^^14^ This Score is an improvement over the NDVI as it considers not just the greenness of certain area but also considers the other contextual factors, including the quality of nature. Thanks to the cross-referencing system with other land classification datasets and incorporating several satellite images over a year, it is able to correct for both water and cloud error in regular NDVI. The score ranges from 0 to 100, with higher scores indicating more abundant and beneficial natural elements. In this study, the city of Houston was divided into different polygons using a 50-m grid and 500-m radius for the NatureScore of each point. Over 13 million points were created to make sure the data is as granular and specific as possible, then each patient address was geocoded and matched to the corresponding point and NatureScore. For the analysis purposes, we categorized the scores into 4 categories: Nature Deficient/Nature Light (0-39), Nature Adequate (40-59), Nature Rich (60-79), and Nature Utopia (80-100). This measure was previously validated against NDVI in a large nationwide U.S. census tract-based study and found a strong correlation (r = 0.87) between NDVI and NatureScore^14^^,^^15^ and it was also used in mental health studies.^16^^,^^17^

#### Dependent variable: cardiovascular risk factors and diseases

Cardiovascular risk factors and ASCVD included in this study were defined based on the ICD-10-CM codes from the patients’ EHRs, listed as a discharge or comorbid diagnosis. The CV risk factors included hypertension, diabetes, dyslipidemia, obesity, and smoking history (Supplemental Tables 3 to 5). ASCVD were defined as the presence of coronary artery disease (CAD), peripheral artery disease (PAD), or stroke (Supplemental Tables 6 to 8).

Using the patient’s recorded weight and height, a body mass index (BMI) was calculated, and an individual was considered obese if had BMI ≥30 kg/m^2^. Dyslipidemia was defined as having a low-density lipoprotein >130 mg/dL, triglycerides >150 mg/dL at any encounter, or ever taking statin, proprotein convertase subtilisin/kexin type 9 (PCSK9) inhibitor, bile acid sequestrants, ezetimibe, fibrates, bempedoic acid, inclisiran, or omega-3 fatty acids.

Cardiovascular risk profiles were based on the number of CV risk factors present: poor CV health (≥3 CV risk factors), average CV health (1-2 CV risk factors), or optimal CV health (0 CV risk factors).^18^^,^^19^

#### Covariates

The following covariates were included: age, sex, race/ethnicity, health insurance, and 2020 Area Deprivation Index version 3.2 (ADI) as a proxy for socioeconomic status. The ADI was developed by the Center for Health Disparities Research at the University of Wisconsin^20^ as a composite score for socioeconomic status at the census-block level.^21^, ^22^, ^23^, ^24^ ADI was assigned to each individual based on their latest residential address and was categorized into quintiles where higher quintiles indicating greater deprivation. Health insurance status was categorized as following: Government plans which included Medicare, Medicaid, pending Medicaid, and Military (TRICARE and CHAMPUS); private insurance which included Blue Cross Blue Shield, Managed Care, Medicaid replacement, and Medicare replacement; self-pay; and others.

#### WalkScore

WalkScore is a tool (0-100) used to quantify the neighborhood walkability in a certain area using the latest recorded patient’s residential address.^25^ This score was developed using the weighted average of several addresses’ scores in a ZIP code while also being weighted by population density. Walkability is calculated after considering the number of amenities and the distance between a certain residential address and these amenities. These amenities included schools, retail stores (eg, grocery stores), food stores, recreational amenities (eg, gyms and parks), and entertainment amenities (eg, theaters). As the distance increases, the score decreases. The score was further validated by several previous studies.^26^, ^27^, ^28^, ^29^, ^30^, ^31^ In this study, we used WalkScores at the zip code level and categorized it as follows: 0 to 19 car-dependent (all errands), 20 to 39 car-dependent (most errands), 40 to 59 somewhat walkable, 60 to 100 very walkable/walker’s paradise.

### Statistical analyses

Participants characteristics were reported using median and interquartile range (IQR) for continuous variables and numbers and frequencies for categorical variables. Kruskal-Wallis H test and Chi-square test were used to compare the distributions across NatureScore categories for continuous and categorial variables, respectively (Table 1).

**Table 1:** Demographics and Prevalence of Cardiovascular Risk Factors and Diseases Among the Total Population

	Total (N = 1,077,181)	Nature Deficient/Nature Light (0-39) (n = 160,818, 15%)	Nature Adequate (40-59) (n = 224,163, 21%)	Nature Rich (60-79) (n = 351,942, 33%)	Nature Utopia (80-100) (n = 340,258, 32%)	*P* Value^a^
NatureScore	69 (51-83)	27 (17-34)	51 (46-56)	71 (66-75)	88 (84-93)	<0.001
Sex						<0.001
Male	442,517 (41%)	66,669 (41%)	90,788 (41%)	142,696 (41%)	142,364 (42%)	
Female	634,664 (59%)	94,149 (59%)	133,375 (59%)	209,246 (59%)	197,894 (58%)	
Age, y	52 (37-67)	47 (33-64)	50 (36-66)	52 (38-67)	55 (40-68)	<0.001
Age by group, y						<0.001
18-39	314,776 (29%)	62,315 (39%)	69,588 (31%)	99,286 (28%)	83,587 (25%)	
40-64	450,291 (42%)	59,916 (37%)	93,717 (42%)	149,426 (42%)	147,232 (43%)	
65-79	243,760 (23%)	29,615 (18%)	47,471 (21%)	80,473 (23%)	86,201 (25%)	
80+	68,354 (6%)	8,972 (6%)	13,387 (6%)	22,757 (6%)	23,238 (7%)	
Race/ethnicity						<0.001
Hispanic (H)	172,066 (16%)	28,645 (18%)	40,684 (18%)	59,142 (17%)	43,595 (13%)	
Non-H White	582,554 (54%)	74,639 (46%)	103,704 (46%)	181,101 (51%)	223,110 (66%)	
Non-H Black	155,766 (14%)	26,571 (17%)	40,419 (18%)	57,845 (16%)	30,931 (9%)	
Non-H Asian	80,454 (7%)	14,857 (9%)	20,795 (9%)	26,370 (7%)	18,432 (5%)	
Others	86,341 (8%)	16,106 (10%)	18,561 (8%)	27,484 (8%)	24,190 (7%)	
ADI National						<0.001
1st quintile (least deprived)	253,168 (24%)	52,114 (32%)	53,986 (24%)	72,663 (21%)	74,405 (22%)	
2nd quintile	319,725 (30%)	33,329 (21%)	60,031 (27%)	113,276 (32%)	113,089 (33%)	
3rd quintile	259,515 (24%)	31,931 (20%)	53,090 (24%)	85,934 (24%)	88,560 (26%)	
4th quintile	165,410 (15%)	28,298 (18%)	37,673 (17%)	55,172 (16%)	44,267 (13%)	
5th quintile (most deprived)	76,240 (7%)	13,996 (9%)	18,975 (8%)	24,018 (7%)	19,251 (6%)	
Unknown	3,123 (0%)	1,150 (1%)	408 (0%)	879 (0%)	686 (0%)	
Health Insurance^b^						<0.001
Government programs	151,093 (14%)	18,048 (11%)	26,725 (12%)	45,172 (13%)	48,477 (14%)	
Private Insurance	530,561 (50%)	75,062 (47%)	111,697 (50%)	176,245 (50%)	167,557 (49%)	
Self-pay	78,305 (7%)	14,681 (9%)	17,189 (8%)	24,737 (7%)	21,698 (6%)	
Others	317,222 (29%)	51,721 (32%)	65,890 (29%)	101,291 (29%)	98,320 (29%)	
WalkScore	27 (18-38)	50 (33-68)	28 (20-45)	26 (18-35)	20 (15-28)	<0.001
WalkScore categories						<0.001
Car dependent (all errands)	436,293 (41%)	19,605 (12%)	77,048 (34%)	154,279 (44%)	185,361 (54%)	
Car dependent (most errands)	377,466 (35%)	54,162 (34%)	89,212 (40%)	142,508 (40%)	91,584 (27%)	
Somewhat walkable	110,652 (10%)	49,212 (31%)	30,776 (14%)	24,915 (7%)	5,749 (2%)	
Very walkable/walker's paradise	26,195 (2%)	19,661 (12%)	3,720 (2%)	2,263 (1%)	551 (0%)	
Unknown	126,575 (12%)	18,178 (11%)	23,407 (10%)	27,977 (8%)	57,013 (17%)	
Urban/Rural						<0.001
Urban	1,063,002 (99%)	158,451 (99%)	222,820 (99%)	350,475 (100%)	331,256 (97%)	
Rural	14,177 (1%)	2,367 (1%)	1,342 (1%)	1,467 (0%)	9,001 (3%)	

Values are median (IQR) or n (%).

ADI = area deprivation index; ASCVD = atherosclerotic cardiovascular disease; BMI = body mass index; CV = cardiovascular; CAD = coronary artery disease; PAD = peripheral artery disease.

a Chi-square test and Kruskal-Wallis test conducted to compare the 4 categories of NatureScore for categorical and continuous non-normally distributed variables, respectively.

b Government plans included Medicare, Medicaid, pending Medicaid, and Military (TRICARE and CHAMPUS); private insurance included Blue Cross Blue Shield, Managed Care, Medicaid replacement, and Medicare replacement.

Logistic regression analyses, presented by odds ratio, were conducted to investigate the relationship between NatureScore and the various cardiovascular outcomes. A-priori set of variables were used to create 4 models: model 1 (unadjusted), model 2 (adjusted for age, sex, and race/ethnicity), model 3 (adjusted for model 2 variables plus ADI), model 4 (adjusted for model 3 variables plus WalkScore).

A multinomial logistic regression using the same variables was conducted to explore the relationship between the NatureScore and the cardiovascular risk profiles (poor, average, and optimal), presented as relative risk ratio (RRR). The multinomial model was chosen after the likelihood ratio test concluded nonproportionality of odds across the categories of the outcome (*P* < 0.001), violating the assumption needed for ordinal logistic regression. In this model, poor (3+ risk factors) cardiovascular risk profile was used the reference category. Lastly, a subgroup analysis was performed using the ASCVD status.

Interaction testing between NatureScore and each covariate was conducted using the likelihood ratio tests and stratified analyses were performed if significant interactions were found. All the statistical analyses were conducted using Stata/MP 17.0 analytical software (StataCorp) with a level of significance of 2-sided *P* value <0.05.

## Results

### Neighborhoods characteristics

Our analytical sample comprised 1,077,181 adults (59% female, 16% Hispanic, 14% non-Hispanic Black) residing in 293 unique zip codes across Houston, Texas. Over half (54%) of our sample lived in neighborhoods with NatureScores of 40 to 80. The median age of included individuals was 52 (IQR: 37-67), with more elderly residing in higher NatureScore neighborhoods (Central Illustration).

**Central Illustration:**
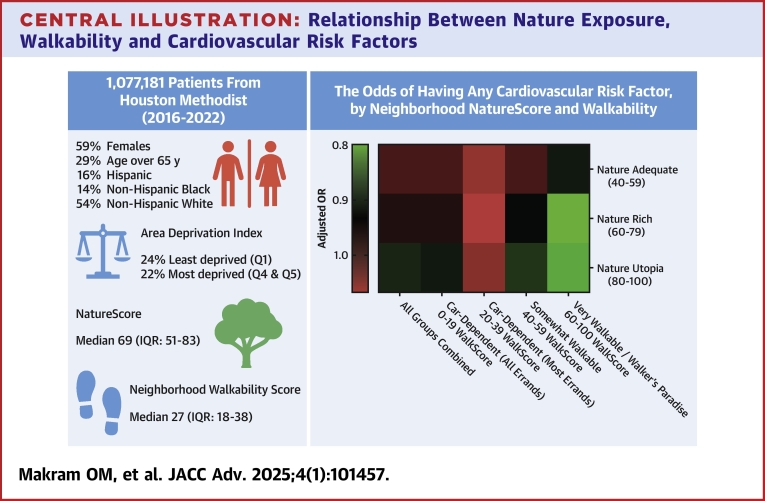
Relationship Between Nature Exposure, Walkability and Cardiovascular Risk Factors

Overall, over 50% of individuals lived in least deprived neighborhoods (lowest 2 ADI quintiles). Notably, areas with NatureScore below 40 had the highest deprivation rates but higher neighborhood walkability. However, we did not find a significant relationship between NatureScore and ADI (r = −0.0009, *P* = 0.326). On the other hand, areas with higher NatureScores had lower WalkScores. Interestingly, there was a moderate negative correlation between NatureScore and WalkScore (r = −0.538, *P* < 0.001) (Table 1).

Upon stratifying by ASCVD status, it was noted that the cohort with ASCVD had higher percentage of elderly (70% vs 25%), males (55% vs 40%), and over 28% (vs 22%) lived in the most deprived neighborhoods, highest 2 ADI quintiles (Supplemental Table 9).

### Cardiovascular risk factors

**Figure 1:**
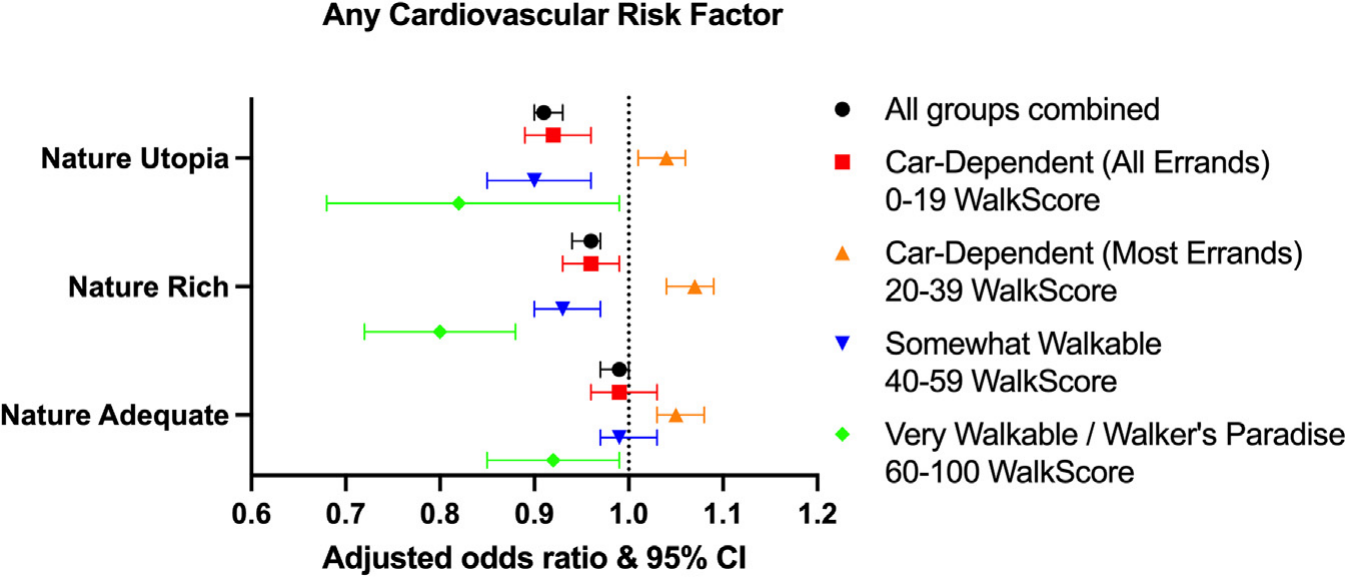
Forest Plot and Heatmap Demonstrating the Association Between Neighborhoods With Various WalkScores and NatureScores and Any Cardiovascular Risk Factor

Across the 4 NatureScore categories, we noticed a higher prevalence of overweight, obesity, hypertension, diabetes, dyslipidemia, and smoking in neighborhoods with NatureScore over 40 (Table 2). A similar finding was found after stratifying by WalkScore (Supplemental Table 10) and running various logistic regression models (Supplemental Table 11). However, on adjusting for WalkScore (Model 4), the relationship was reversed, with individuals living in neighborhoods with NatureScores ≥60 had significantly lower odds of any CV risk factors (NatureScore 60-79, aOR: 0.96, 95% CI: 0.94-0.97, *P* < 0.001; NatureScore ≥80, aOR: 0.91, 95% CI: 0.90-0.93, *P* < 0.001) (Figure 1). Again, individual risk factors followed the same pattern after adjusting for the neighborhood walkability (Table 3).

**Table 2:** Prevalence of Cardiovascular Risk Factors and Diseases Among the Total Population

	Total (N = 1,077,181)	Nature Deficient/Nature Light (0-39) (n = 160,818, 15%)	Nature Adequate (40-59) (n = 224,163, 21%)	Nature Rich (60-79) (n = 351,942, 33%)	Nature Utopia (80-100) (n = 340,258, 32%)	*P* Value^a^
BMI, kg/m^2^						<0.001
Underweight (<18.5)	17,221 (2%)	2,879 (2%)	3,513 (2%)	5,458 (2%)	5,371 (2%)	
Normal (18.5-24.9)	270,534 (25%)	44,430 (28%)	55,760 (25%)	86,157 (24%)	84,187 (25%)	
Overweight (25-29.9)	314,430 (29%)	45,048 (28%)	64,328 (29%)	102,772 (29%)	102,282 (30%)	
Obesity class I (30-34.9)	199,779 (19%)	25,334 (16%)	41,561 (19%)	66,910 (19%)	65,974 (19%)	
Obesity class II (35-39.9)	94,611 (9%)	11,685 (7%)	19,625 (9%)	32,124 (9%)	31,177 (9%)	
Obesity class III (40+)	67,778 (6%)	9,077 (6%)	14,477 (6%)	23,116 (7%)	21,108 (6%)	
Unknown	112,828 (10%)	22,365 (14%)	24,899 (11%)	35,405 (10%)	30,159 (9%)	
CV risk factors						
Any CV risk	733,214 (68%)	98,340 (61%)	151,501 (68%)	243,409 (69%)	239,964 (71%)	<0.001
Hypertension	449,352 (42%)	56,633 (35%)	91,393 (41%)	150,214 (43%)	151,112 (44%)	<0.001
Diabetes mellitus	179,249 (17%)	22,767 (14%)	38,006 (17%)	61,205 (17%)	57,271 (17%)	<0.001
Dyslipidemia	316,104 (29%)	42,287 (26%)	64,692 (29%)	104,329 (30%)	104,796 (31%)	<0.001
Obesity	362,168 (34%)	46,096 (29%)	75,663 (34%)	122,150 (35%)	118,259 (35%)	<0.001
Smoking ever	294,028 (27%)	37,515 (23%)	60,369 (27%)	98,073 (28%)	98,071 (29%)	<0.001
ASCVD						
Any ASCVD	102,238 (9%)	12,544 (8%)	20,456 (9%)	33,638 (10%)	35,600 (10%)	<0.001
CAD	67,392 (6%)	8,066 (5%)	13,237 (6%)	22,180 (6%)	23,909 (7%)	<0.001
PAD	20,650 (2%)	2,447 (2%)	4,163 (2%)	7,031 (2%)	7,009 (2%)	<0.001
Stroke	37,866 (4%)	4,859 (3%)	7,709 (3%)	12,501 (4%)	12,797 (4%)	<0.001

Values are n (%).

ADI = area deprivation index; ASCVD = atherosclerotic cardiovascular disease; BMI = body mass index; CAD = coronary artery disease; CV = cardiovascular; IQR = inter-quartile range; PAD = peripheral artery disease.

a Chi-square test and Kruskal-Wallis test conducted to compare the 4 categories of NatureScore for categorical and continuous non-normally distributed variables, respectively.

**Table 3:** Multivariable Logistic Regression for Cardiovascular Risk Factors Across Various NatureScore Groups, Stratified by Neighborhood Walkability

		Multivariable Analysis After Stratification Using Model 4			
	Model 4	Car-Dependent (All Errands) (0-19)	Car-Dependent (Most Errands) (20-39)	Somewhat Walkable (40-59)	Very Walkable/Walker's Paradise (60-100)
Any CV risk factor a(LR test, *P* < 0.001)					
Nature Adequate	0.99 (0.97-1.00), *P* = 0.092	0.99 (0.96-1.03), *P* = 0.721	**1.05 (1.03-1.08), *P* < 0.001**	0.99 (0.97-1.03), *P* = 0.998	**0.92 (0.85-0.99), *P* = 0.034**
Nature Rich	**0.96 (0.94-0.97), *P* < 0.001**	**0.96 (0.93-0.99), *P* = 0.020**	**1.07 (1.04-1.09), *P* < 0.001**	**0.93 (0.90-0.97), *P* < 0.001**	**0.80 (0.72-0.88), *P* < 0.001**
Nature Utopia	**0.91 (0.90-0.93), *P* < 0.001**	**0.92 (0.89-0.96), *P* < 0.001**	**1.04 (1.01-1.06), *P* = 0.005**	**0.90 (0.85-0.96), *P* = 0.001**	**0.82 (0.68-0.99), *P* = 0.038**
Hypertension a(LR test, *P* < 0.001)					
Nature Adequate	**0.97 (0.96-0.99), *P* = 0.002**	0.97 (0.94-1.01), *P* = 0.178	**1.04 (1.02-1.07), *P* = 0.001**	**0.94 (0.91-0.98), *P* = 0.002**	0.92 (0.83-1.01), *P* = 0.069
Nature Rich	**0.95 (0.94-0.97), *P* < 0.001**	**0.96 (0.92-0.99), *P* = 0.013**	**1.04 (1.01-1.06), *P* = 0.003**	**0.93 (0.89-0.97), *P* < 0.001**	**0.83 (0.74-0.92), *P* = 0.001**
Nature Utopia	**0.91 (0.90-0.93), *P* < 0.001**	0.93 (0.90-0.97), *P* < 0.001	1.00 (0.97-1.02), *P* = 0.726	**0.87 (0.82-0.94), *P* < 0.001**	0.89 (0.72-1.09), *P* = 0.254
Diabetes Mellitus a(LR test, *P* < 0.001)					
Nature Adequate	1.00 (0.98-1.02), *P* = 0.942	**0.94 (0.90-0.99), *P* = 0.010**	**1.04 (1.01-1.07), *P* = 0.023**	1.02 (0.97-1.07), *P* = 0.411	0.94 (0.81-1.08), *P* = 0.362
Nature Rich	**0.98 (0.96-1.00), *P* = 0.030**	**0.94 (0.90-0.98), *P* = 0.003**	1.02 (0.99-1.05), *P* = 0.247	0.95 (0.91-0.99), *P* = 0.046	0.96 (0.81-1.14), *P* = 0.628
Nature Utopia	**0.92 (0.90-0.94), *P* < 0.001**	**0.91 (0.87-0.95), *P* < 0.001**	**0.96 (0.93-0.99), *P* = 0.010**	**0.86 (0.79-0.94), *P* = 0.001**	1.05 (0.77-1.43), *P* = 0.770
Dyslipidemia a(LR test, *P* < 0.001)					
Nature Adequate	1.00 (0.98-1.01), *P* = 0.717	**1.06 (1.02-1.10), *P* = 0.001**	**1.03 (1.01-1.06), *P* = 0.015**	1.02 (0.99-1.06), *P* = 0.203	0.98 (0.89-1.06), *P* = 0.565
Nature Rich	**0.98 (0.96-0.99), *P* = 0.009**	**1.04 (1.00-1.07), *P* = 0.033**	**1.04 (1.02-1.07), *P* = 0.001**	0.99 (0.96-1.03), *P* = 0.766	**0.84 (0.75-0.93), *P* = 0.001**
Nature Utopia	**0.96 (0.95-0.98), *P* < 0.001**	1.01 (0.97-1.04), *P* = 0.757	**1.05 (1.03-1.08), *P* < 0.001**	0.99 (0.94-1.06), *P* = 0.910	0.88 (0.72-1.07), *P* = 0.187
Obesity a(LR test, *P* < 0.001)					
Nature Adequate	**1.02 (1.00-1.04), *P* = 0.016**	1.00 (0.96-1.03), *P* = 0.810	**1.05 (1.03-1.08), *P* < 0.001**	**1.04 (1.00-1.07), *P* = 0.032**	0.96 (0.87-1.06), *P* = 0.422
Nature Rich	1.00 (0.99-1.02), *P* = 0.795	0.97 (0.94-1.01), *P* = 0.106	**1.07 (1.04-1.09), *P* < 0.001**	0.97 (0.93-1.00), *P* = 0.059	**0.82 (0.72-0.94), *P* = 0.003**
Nature Utopia	**0.96 (0.94-0.97), *P* < 0.001**	**0.95 (0.92-0.98), *P* = 0.002**	**1.05 (1.03-1.07), *P* < 0.001**	**0.89 (0.83-0.96), *P* = 0.002**	0.87 (0.67-1.12), *P* = 0.272
Smoking a(LR test, *P* < 0.001)					
Nature Adequate	1.00 (0.98-1.01), *P* = 0.710	**0.96 (0.92-0.99), *P* = 0.033**	**1.04 (1.01-1.07), *P* = 0.003**	1.00 (0.96-1.04), *P* = 0.992	0.97 (0.88-1.07), *P* = 0.514
Nature Rich	**0.98 (0.96-0.99), *P* = 0.003**	**0.94 (0.91-0.98), *P* = 0.001**	**1.06(1.01-1.06), *P* = 0.004**	**0.92(0.89-0.96), *P* < 0.001**	**0.88(0.78-0.99), *P* = 0.046**
Nature Utopia	**0.94 (0.92-0.95), *P* < 0.001**	**0.92 (0.89-0.96), *P* < 0.001**	0.98 (0.96-1.01), *P* = 0.215	**0.88 (0.82-0.94), *P* < 0.001**	1.03 (0.82-1.28), *P* = 0.813
Any ASCVD a(LR test, *P* < 0.001)					
Nature Adequate	0.99 (0.96-1.02), *P* = 0.491	1.02 (0.96-1.09), *P* = 0.473	1.03 (0.99-1.07), *P* = 0.149	1.04 (0.98-1.09), *P* = 0.189	1.06 (0.93-1.23), *P* = 0.351
Nature Rich	**0.95 (0.93-0.98), *P* < 0.001**	0.99 (0.94-1.05), *P* = 0.749	0.99 (0.96-1.04), *P* = 0.917	1.01 (0.96-1.07), *P* = 0.686	0.94 (0.79-1.11), *P* = 0.455
Nature Utopia	**0.96 (0.93-0.98), *P* = 0.002**	1.02 (0.96-1.08), *P* = 0.580	0.98 (0.94-1.02), *P* = 0.300	1.05 (0.95-1.16), *P* = 0.337	1.05 (0.79-1.40), *P* = 0.730
CAD a(LR test, *P* = 0.001)					
Nature Adequate	0.98 (0.95-1.01), *P* = 0.154	1.00 (0.93-1.07), *P* = 0.959	1.03 (0.98-1.08), *P* = 0.319	1.06 (0.99-1.13), *P* = 0.087	1.01 (0.86-1.20), *P* = 0.874
Nature Rich	**0.95(0.92-0.98), *P* = 0.002**	0.98 (0.91-1.04), *P* = 0.481	1.02 (0.98-1.07), *P* = 0.345	1.02 (0.95-1.10), *P* = 0.540	0.85 (0.70-1.04), *P* = 0.122
Nature Utopia	**0.95 (0.92-0.98), *P* = 0.003**	1.00 (0.94-1.07), *P* = 0.951	1.00 (0.95-1.05), *P* = 0.891	1.00 (0.88-1.13), *P* = 0.958	1.05 (0.75-1.47), *P* = 0.763
PAD a(LR test, *P* < 0.001)					
Nature Adequate	0.97 (0.92-1.03), *P* = 0.293	0.97 (0.86-1.10), *P* = 0.663	0.99 (0.92-1.07), *P* = 0.785	1.00 (0.89-1.13), *P* = 0.963	**1.53(1.11-2.12), *P* = 0.010**
Nature Rich	0.95 (0.90-1.00), *P* = 0.046	0.98 (0.87-1.10), *P* = 0.765	0.96 (0.89-1.03), *P* = 0.239	1.03 (0.91-1.16), *P* = 0.696	0.93 (0.59-1.45), *P* = 0.735
Nature Utopia	**0.93(0.88-0.98), *P* = 0.012**	1.01 (0.90-1.13), *P* = 0.928	**0.91(0.84-0.98), *P* = 0.018**	0.99 (0.79-1.24), *P* = 0.937	0.60 (0.22-1.65), *P* = 0.326

Values are aOR (95% CI), *P* value.

aOR = adjusted odds ratio; ASCVD = atherosclerotic cardiovascular disease; CAD = coronary artery disease; CV = cardiovascular; LR = likelihood ratio test; PAD = peripheral artery disease.

**Bold** text indicates significant results.

Model 4: Adjusted for age, sex, race/ethnicity, area deprivation index, and WalkScore.

a Interaction testing between NatureScore and WalkScore using likelihood ratio test. No statistically significant interaction was found between NatureScore and WalkScore in stroke model (LR test, *P* = 0.396).

A significant interaction was observed between NatureScore and WalkScore among all CV risk factors as the outcome (LR, *P* < 0.001). Upon further stratification by WalkScore, we found that in neighborhoods with WalkScore ≥40, higher NatureScores were associated with lower prevalence of any CV risk factor. For instance, in neighborhoods with WalkScore 40 to 59, individuals living in neighborhoods with NatureScore 60 to 79 (aOR: 0.93, 95% CI: 0.90-0.97, *P* < 0.001) and NatureScore 80+ (aOR: 0.90, 95% CI: 0.85-0.96, *P* < 0.001) had lower odds of any CV risk factor. Moreover, neighborhoods with the highest WalkScores (≥60) had lower prevalence of any CV risk factor in all neighborhoods with NatureScore above 40 (NatureScore 40-59, aOR: 0.92, 95% CI: 0.85-0.99, *P* = 0.034; 60-79, aOR: 0.80, 95% CI: 0.72-0.88, *P* < 0.001; ≥80, aOR: 0.82, 95% CI: 0.68-0.99, *P* = 0.038). When considering individual risk factors, those living in neighborhoods with higher NatureScores had lower CV risk factors mostly in areas with WalkScore 40 to 59 (Table 3). However, it is to be noted that only 2% of all individuals lived in areas with WalkScore 60+ and only 0.16% of individuals lived in areas with NatureScore 80+ and WalkScore 60+ (Table 1).

Multinomial regression analysis revealed that higher NatureScores were associated with a lower likelihood of having an optimal CV risk profile. However, on stratifying by WalkScore, individuals living in neighborhoods with WalkScore of 40 to 59 and NatureScore ≥60 were more likely to have optimal CV risk profile (NatureScore 60-79, RRR: 1.09, 95% CI: 1.04-1.14, *P* = 0.001; NatureScore ≥80, RRR: 1.20, 95% CI: 1.09-1.32, *P* < 0.001) (Table 4).

**Table 4:** Multinomial Logistic Regression for Cardiovascular Risk Profiles Across Various NatureScore Groups, Stratified by Neighborhood walkability

		Multinomial Logistic Regression after Stratification Using Model 4			
	Model 4	Car-Dependent (all Errands) (0-19)	Car-Dependent (Most Errands) (20-39)	Somewhat Walkable (40-59)	Very Walkable/Walker's Paradise (60-100)
Optimal (0 risk factors) vs Poor (3+) Risk Profile a(LR test, *P* < 0.001)					
Nature Adequate	**0.94 (0.92-0.96), *P* < 0.001**	1.03 (1.08-1.15), *P* = 0.163	**0.92 (0.89-0.95), *P* < 0.001**	1.01 (0.97-1.06), *P* = 0.633	**1.20 (1.06-1.37), *P* = 0.006**
Nature Rich	**0.95 (0.93-0.97), *P* < 0.001**	**1.08 (1.03-1.13), *P* = 0.002**	**0.92 (0.89-0.95), *P* < 0.001**	**1.09 (1.04-1.14), *P* = 0.001**	**1.54 (1.31-1.81), *P* < 0.001**
Nature Utopia	**0.97 (0.95-0.99), *P* = 0.014**	**1.15 (1.10-1.20), *P* < 0.001**	**0.95 (0.92-0.99), *P* = 0.005**	**1.20 (1.09-1.32), *P* < 0.001**	**1.39 (1.04-1.85), *P* = 0.026**
Average (1-2) vs Poor (3+) Risk Profile a(LR test, *P* < 0.001)					
Nature Adequate	0.98 (0.96-1.00), *P* = 0.064	1.04 (0.99-1.08), *P* = 0.097	**0.96 (0.94-0.99), *P* = 0.010**	1.02 (0.97-1.06), *P* = 0.438	1.05 (0.92-1.18), *P* = 0.480
Nature Rich	**0.98 (0.97-1.00), *P* = 0.046**	1.04 (1.00-1.08), *P* = 0.062	**0.97 (0.94-0.99), *P* = 0.022**	1.03 (0.98-1.08), *P* = 0.209	1.16 (1.00-1.36), *P* = 0.051
Nature Utopia	1.00 (0.98-1.02), *P* = 0.914	**1.06 (1.02-1.11), *P* = 0.000**	1.00 (0.97-1.03), *P* = 0.949	**1.13 (1.04-1.22), *P* = 0.005**	0.98 (0.75-1.28), *P* = 0.878

Values are RRR (95% CI), *P* value.

CI = Confidence intervals; RRR = Relative risk ratio.

**Bold** text indicates significant results.

Model 4: Adjusted for age, sex, race/ethnicity, area deprivation index, and WalkScore.

a Interaction testing between NatureScore and WalkScore using likelihood ratio test.

### Cardiovascular diseases

The overall prevalence of ASCVD was 9%, with CAD being the most reported cardiac disease (6%) (Table 1). We demonstrated a higher prevalence of ASCVD and CAD in neighborhoods with NatureScore ≥40 (Table 2). A minimal difference was noted after stratifying by WalkScore (Supplemental Table 10).

**Figure 2:**
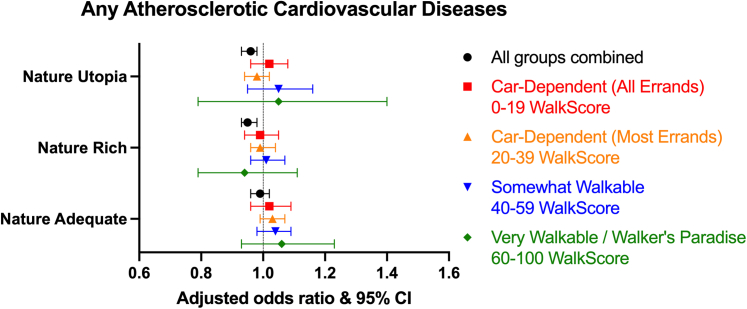
Forest Plot Demonstrating the Association Between Neighborhoods With Various WalkScores and NatureScores and Atherosclerotic Cardiovascular Diseases

Similar to the findings in CV risk factors, ASCVD was higher in areas with higher NatureScores using models 1, 2 and 3. However, on adjusting for WalkScore, individuals living in neighborhoods with NatureScores 60 to 79 (aOR: 0.95, 95% CI: 0.93-0.98, *P* < 0.001) and 80+ (aOR: 0.96, 95% CI: 0.93-0.98, *P* = 0.002) showed lower odds of having ASCVD (Figure 2). Similar findings were observed for CAD and PAD, but not for stroke (Table 3). While a significant interaction was found between NatureScore and WalkScore in CAD and PAD models, no meaningful or statistically significant results were observed after stratification (Table 3). No specific direction in outcomes was noted, in terms of prevalence or after multivariable regression, upon stratification by sex, age, race/ethnicity, ADI, or ASCVD status (Supplemental Tables 12 to 17).

## Discussion

In this study, we aimed to investigate the relationship between nature exposure using NatureScore and the prevalence of cardiovascular diseases and risk factors, while examining how neighborhood walkability modifies this relationship. Although we initially demonstrated higher prevalence of both CV risk factors and ASCVD among neighborhoods with higher NatureScores, this relationship was altered after considering neighborhood walkability. After adjusting for WalkScore, we found that individuals living in neighborhoods with higher NatureScore had lower prevalence of CV risk factors and diseases. Furthermore, our findings revealed that those residing in neighborhoods with at least a WalkScore of 60 and a NatureScore of 40 had a better CV risk profiles and lower prevalence of CV risk factors. Yet, no significant difference was observed in ASCVD prevalence upon stratification by walkability.

The discrepancy between our findings in CV risk factors and ASCVD could be explained by several factors. As ASCVD represents a more advanced stage of CVD, it may be less immediately responsive to environmental factors compared to the more immediate risk factors. Also, a temporal disconnection between might be present between the current environmental exposures and ASCVD development, which might take years to occur. Also, an unexpected finding in our study was that individuals living in car-dependent (most errands) might show higher prevalence of CV risk factors than those living in car-dependent (all-errands). We believe that this finding might be related to demographics and social factors as we demonstrated in our previous paper that individuals living in car-dependent (most-errands) were more likely to self-identify as Hispanic or NHB (38% vs 26%) and to live in neighborhoods with the 2 highest ADI quintiles (25% vs 13%), as well as being over the age 65 (30% vs 27%).^10^

In an earlier study conducted by our team, we demonstrated better CV risk profiles and lower prevalence of CV risk factors in neighborhoods with higher walkability.^10^ Our current findings further underscores the role of accessibility, in terms of neighborhood walkability, in obtaining the beneficial impact of nature exposure repeatedly reported in previous studies.^32^, ^33^, ^34^, ^35^ This is also the first study to utilize the innovative multifaceted NatureScore tool to study the impact of nature exposure on cardiovascular health, capturing a myriad of nature elements that impact individuals’ health while overcoming the limitations of other previously used measures.

Our findings are consistent with previous studies worldwide. In a study conducted using the entire population of England, Mitchell et al. demonstrated lower mortality rates due to circulatory disease among individuals living in the most green areas, using the classification provided by the land use databases.^2^ A study in Canada utilizing the NDVI as a measure of greenness in urban setting showed lower mortality rates for cardiopulmonary, CV, stroke, and ischemic heart disease among individuals living in areas with higher greenness scores.^4^ Pereira et al. found a weak evidence linking mean greenness scores and CAD in Australia using NDVI but demonstrated a protective effect of variability in neighborhood greenness on CAD and stroke, underscoring the importance of biodiversity.^3^ Additionally, a recent systematic review on nature-based interventions revealed beneficial cardiovascular outcomes associated with forest bathing, physical activity occurring in natural environment, vegetable gardening, and even nature viewing.^36^ Conversely, Donovan et al. showed that the loss of tree canopy from 2002 to 2007 significantly increased cardiovascular mortality with over 16 additional deaths per 100,000 adults per year.^37^

Our results not only align with, but significantly extend the previous MESA Neighborhood Study results on physical activity and walking environments.^38^^,^^39^ These earlier studies laid crucial groundwork, demonstrating that higher density of recreational resources directly correlated with increased physical activity,^38^ and that higher neighborhood walkability was associated with more walking for transportation.^39^ Our research build upon that foundation by integrating both NatureScore and WalkScore to provide a more comprehensive understanding of how the interplay between nature exposure and accessibility influences cardiovascular health.

It is crucial to understand the underlying complexity of mechanistic pathways between nature exposure and cardiovascular health. One proposed mechanism is that green spaces encourage healthier behaviors like physical activity,^40^ while lowering stress,^41^ improving mental health,^17^^,^^42^ improving sleep,^43^ and reducing air pollution.^44^^,^^45^ However, for individuals to benefit from nature exposure, it must be accessible and attractive to utilize, yet little is known about the interaction between neighborhood walkability and residential greenness. In other earlier studies in Australia, Canada, and the USA, the authors consistently demonstrated a negative association was found between neighborhood walkability and greenness, similar to our findings where areas with higher NatureScores had lower walkability scores.^46^, ^47^, ^48^ This suggests that areas with higher walkability, where people typically walk and shop, not only might have busier roads and higher pollution but also lack greenness where it is needed more. Conversely, in neighborhoods where greenness prevails, lower walkability and accessibility may hinder the ultimate utilization of these natural resources.

### Study Limitations

Our study is the first to study the impact of nature exposure using the composite score, NatureScore while also considering the area deprivation and neighborhood walkability. This was further strengthened by the large sample size of over 1 million patients at the individual level.

Despite the strengths, some limitations still exist, including the lack of measurements for perceived safety, crime rates, traffic, and other neighborhood characteristics,^49^^,^^50^ such as access to healthy food options and facilities to exercise, that may contribute to individuals’ decision to spend time in nature. We acknowledge limitations related to such scores which do not account for the actual time spent in nature and activities performed during that time, potentially leading to residual confounding. Additionally, we are aware that, while the sample size was large, all individuals were included from single institution dataset which might limit generalizability and some patient data from other institutions might not be available in that dataset due to care fragmentation. Furthermore, ICD coding for the outcomes are associated with limitations that include inaccurate and inconsistent coding, up-coding, identifying the cause of outcome, or the severity of the outcome. Finally, the observational nature of the study limits causal inference. Future studies should not only address these limitations but also test our hypothesis and NatureScore in other settings for external validation, including different geographical areas, areas with different social norms where people’s interactions with nature may differ, and using different areal units other than the zip code.

## Conclusions

This large cross-sectional study reveals the complex interplay between residential nature exposure, neighborhood walkability, and cardiovascular health. Initially, higher nature exposure measured by NatureScore was associated with increased cardiovascular risk factors and diseases. However, after accounting for neighborhood walkability, this relationship reversed, suggesting that individuals residing in highly walkable neighborhoods with abundant nature exposure (NatureScore ≥60 and WalkScore ≥40) are more likely to have optimal cardiovascular risk profile and lower odds of cardiovascular risk factors. Additionally, higher nature exposure was associated with lower odds of both coronary and peripheral artery disease after adjusting for walkability.

These findings underscore the critical importance of urban planning strategies that integrate both walkability and green spaces. Our results call for immediate action from urban planners and policy makers to prioritize the development of accessible, walkable, and nature-rich environments in all communities. Such efforts could serve as powerful public health interventions, potentially reducing cardiovascular disease burden across diverse populations. Future research should focus on developing and evaluating interventions that increase both nature exposure and walkability, particularly in underserved areas to ensure equitable access to these health-promoting environments.

Perspectives **COMPETENCY IN MEDICAL KNOWLEDGE:** Recognize the influence of neighborhood nature exposure and walkability on cardiovascular risk factors and atherosclerotic cardiovascular disease. Incorporate assessment of patients' residential environments into cardiovascular risk stratification and lifestyle counseling. Collaborate with urban planners and public health officials to advocate for health-promoting neighborhood designs.

**TRANSLATIONAL OUTLOOK:** Conduct longitudinal studies to establish causal relationships between changes in neighborhood nature exposure and walkability and long-term cardiovascular outcomes. Develop standardized tools for assessing combined nature exposure and walkability that can be easily implemented in clinical and research settings. Investigate biological mechanisms underlying the observed associations between nature exposure, walkability, and cardiovascular health. Design and conduct community-based intervention studies that manipulate both nature exposure and walkability in urban environments, particularly in underserved areas.

## Data sharing statement

Houston Methodist data registry is available at the Houston Methodist Hospital and has access restricted to researchers with IRB approval and access to the data is available upon request.

## Funding support and author disclosure

The authors have reported that they have no relationships relevant to the contents of this paper to disclose.
