# Supplementary material for: Interplay Between Residential Nature Exposure and Walkability and Their Association with Cardiovascular Health
Source: JACC Adv. 2024 Dec 17;4(1):101457. doi: 10.1016/j.jacadv.2024.101457 (PMC11719309; doi:10.1016/j.jacadv.2024.101457)
Supplement: Supplemental Material [file mmc1.pdf]

Supplementary Material

Table S1. STROBE Statement—checklist of items that should be included in reports of observational studies.

	Item No.	Recommendation	Page No.
**Title and abstract**	1	(*a*) Indicate the study's design with a commonly used term in the title or the abstract	1-3
		(*b*) Provide in the abstract an informative and balanced summary of what was done and what was found	3-4
**Introduction**			
Background/rationale	2	Explain the scientific background and rationale for the investigation being reported	5
Objectives	3	State specific objectives, including any prespecified hypotheses	5
**Methods**			
Study design	4	Present key elements of study design early in the paper	5
Setting	5	Describe the setting, locations, and relevant dates, including periods of recruitment, exposure, follow-up, and data collection	5-6
Participants	6	(*a*) *Cohort study*—Give the eligibility criteria, and the sources and methods of selection of participants. Describe methods of follow-up<br>*Case-control study*—Give the eligibility criteria, and the sources and methods of case ascertainment and control selection. Give the rationale for the choice of cases and controls<br>*Cross-sectional study*—Give the eligibility criteria, and the sources and methods of selection of participants	5-6
		(*b*) *Cohort study*—For matched studies, give matching criteria and number of exposed and unexposed<br>*Case-control study*—For matched studies, give matching criteria and the number of controls per case	5-6
Variables	7	Clearly define all outcomes, exposures, predictors, potential confounders, and effect modifiers. Give diagnostic criteria, if applicable	6-8
Data sources/ measurement	8*	For each variable of interest, give sources of data and details of methods of assessment (measurement). Describe comparability of assessment methods if there is more than one group	*5-8*
Bias	9	Describe any efforts to address potential sources of bias	8-9

Study size	10	Explain how the study size was arrived at	8-9
Quantitative variables	11	Explain how quantitative variables were handled in the analyses. If applicable, describe which groupings were chosen and why	7-8
Statistical methods	12	(*a*) Describe all statistical methods, including those used to control for confounding	8-9
		(*b*) Describe any methods used to examine subgroups and interactions	8-9
		(*c*) Explain how missing data were addressed	8-9
		(*d*) *Cohort study*—If applicable, explain how loss to follow-up was addressed<br><br>*Case-control study*—If applicable, explain how matching of cases and controls was addressed<br><br>*Cross-sectional study*—If applicable, describe analytical methods taking account of sampling strategy	8-9
		(*e*) Describe any sensitivity analyses	N/A
**Results**			
Participants	13*	(a) Report numbers of individuals at each stage of study—eg numbers potentially eligible, examined for eligibility, confirmed eligible, included in the study, completing follow-up, and analysed	9
		(b) Give reasons for non-participation at each stage	N/A
		(c) Consider use of a flow diagram	N/A
Descriptive data	14*	(a) Give characteristics of study participants (eg demographic, clinical, social) and information on exposures and potential confounders	9
		(b) Indicate number of participants with missing data for each variable of interest	9
		(c) *Cohort study*—Summarise follow-up time (eg, average and total amount)	9
Outcome data	15*	*Cohort study*—Report numbers of outcome events or summary measures over time	*10-11*
		Case-control study—Report numbers in each exposure category, or summary measures of exposure	*N/A*
		Cross-sectional study—Report numbers of outcome events or summary measures	*N/A*
Main results	16	(*a*) Give unadjusted estimates and, if applicable, confounder-adjusted estimates and their precision (e.g., 95% confidence interval). Make clear which confounders were adjusted for and why they were included	10-11

		(*b*) Report category boundaries when continuous variables were categorized	10-11
		(*c*) If relevant, consider translating estimates of relative risk into absolute risk for a meaningful time period	10-11
Other analyses	17	Report other analyses done—e.g. analyses of subgroups and interactions, and sensitivity analyses	10-11
**Discussion**			
Key results	18	Summarise key results with reference to study objectives	11-12
Limitations	19	Discuss limitations of the study, taking into account sources of potential bias or imprecision. Discuss both direction and magnitude of any potential bias	13-14
Interpretation	20	Give a cautious overall interpretation of results considering objectives, limitations, multiplicity of analyses, results from similar studies, and other relevant evidence	14
Generalisability	21	Discuss the generalisability (external validity) of the study results	14
**Other information**			
Funding	22	Give the source of funding and the role of the funders for the present study and, if applicable, for the original study on which the present article is based	14

STROBE: Strengthening the Reporting of Observational Studies in Epidemiology; *Give information separately for cases and controls in case-control studies and, if applicable, for exposed and unexposed groups in cohort and cross-sectional studies.

Note: An Explanation and Elaboration article discusses each checklist item and gives methodological background and published examples of transparent reporting. The STROBE checklist is best used in conjunction with this article (freely available on the Web sites of PLoS Medicine at http://www.plosmedicine.org/, Annals of Internal Medicine at http://www.annals.org/, and Epidemiology at http://www.epidem.com/). Information on the STROBE Initiative is available at www.strobe-statement.org.

Table S2. List of outpatient encounter types used in the registry.

	List of outpatient encounter types
**1**	E-visit
**2**	Employee health
**3**	Employee wellness
**4**	Executive wellness
**5**	Multidisciplinary visit
**6**	Office visit
**7**	Telemedicine
**8**	Telemedicine clinical staff
**9**	Virtual collaborative care
**10**	Consult
**11**	Nurse only
**12**	Scheduled phone consult
**13**	Telephone consult

Table S3. List of ICD-10 CM codes used to define Hypertension.

ICD-10 CM CODE	ICD-10 CM CODE DESCRIPTION
**I10**	Essential (primary) hypertension
**I11.0**	Hypertensive heart disease with heart failure
**I11.9**	Hypertensive heart disease without heart failure
**I12.0**	Hypertensive chronic kidney disease with stage 5 chronic kidney disease or end stage renal disease
**I12.9**	Hypertensive chronic kidney disease with stage 1 through stage 4 chronic kidney disease, or unspecified chronic kidney disease
**I13.0**	Hypertensive heart and chronic kidney disease with heart failure and stage 1 through stage 4 chronic kidney disease, or unspecified chronic kidney disease
**I13.10**	Hypertensive heart and chronic kidney disease without heart failure, with stage 1 through stage 4 chronic kidney disease, or unspecified chronic kidney disease
**I13.11**	Hypertensive heart and chronic kidney disease without heart failure, with stage 5 chronic kidney disease, or end stage renal disease
**I13.2**	Hypertensive heart and chronic kidney disease with heart failure and with stage 5 chronic kidney disease, or end stage renal disease
**I15.0**	Renovascular hypertension
**I15.1**	Hypertension secondary to other renal disorders

**I15.2**	Hypertension secondary to endocrine disorders
**I15.8**	Other secondary hypertension
**I15.9**	Secondary hypertension, unspecified

Table S4. List of ICD-10 CM codes used to define Diabetes Mellitus.

ICD-10 CM	ICD-10 CM DESCRIPTION
**E10.10**	Type 1 diabetes mellitus with ketoacidosis without coma
**E10.11**	Type 1 diabetes mellitus with ketoacidosis with coma
**E10.21**	Type 1 diabetes mellitus with diabetic nephropathy
**E10.22**	Type 1 diabetes mellitus with diabetic chronic kidney disease
**E10.29**	Type 1 diabetes mellitus with other diabetic kidney complication
**E10.311**	Type 1 diabetes mellitus with unspecified diabetic retinopathy with macular edema
**E10.319**	Type 1 diabetes mellitus with unspecified diabetic retinopathy without macular edema
**E10.3211**	Type 1 diabetes mellitus with mild nonproliferative diabetic retinopathy with macular edema, right eye
**E10.3212**	Type 1 diabetes mellitus with mild nonproliferative diabetic retinopathy with macular edema, left eye
**E10.3213**	Type 1 diabetes mellitus with mild nonproliferative diabetic retinopathy with macular edema, bilateral
**E10.3219**	Type 1 diabetes mellitus with mild nonproliferative diabetic retinopathy with macular edema, unspecified eye
**E10.3291**	Type 1 diabetes mellitus with mild nonproliferative diabetic retinopathy without macular edema, right eye
**E10.3292**	Type 1 diabetes mellitus with mild nonproliferative diabetic retinopathy without macular edema, left eye
**E10.3293**	Type 1 diabetes mellitus with mild nonproliferative diabetic retinopathy without macular edema, bilateral
**E10.3299**	Type 1 diabetes mellitus with mild nonproliferative diabetic retinopathy without macular edema, unspecified eye

**E10.3311**	Type 1 diabetes mellitus with moderate nonproliferative diabetic retinopathy with macular edema, right eye
**E10.3312**	Type 1 diabetes mellitus with moderate nonproliferative diabetic retinopathy with macular edema, left eye
**E10.3313**	Type 1 diabetes mellitus with moderate nonproliferative diabetic retinopathy with macular edema, bilateral
**E10.3319**	Type 1 diabetes mellitus with moderate nonproliferative diabetic retinopathy with macular edema, unspecified eye
**E10.3391**	Type 1 diabetes mellitus with moderate nonproliferative diabetic retinopathy without macular edema, right eye
**E10.3392**	Type 1 diabetes mellitus with moderate nonproliferative diabetic retinopathy without macular edema, left eye
**E10.3393**	Type 1 diabetes mellitus with moderate nonproliferative diabetic retinopathy without macular edema, bilateral
**E10.3399**	Type 1 diabetes mellitus with moderate nonproliferative diabetic retinopathy without macular edema, unspecified eye
**E10.3411**	Type 1 diabetes mellitus with severe nonproliferative diabetic retinopathy with macular edema, right eye
**E10.3412**	Type 1 diabetes mellitus with severe nonproliferative diabetic retinopathy with macular edema, left eye
**E10.3413**	Type 1 diabetes mellitus with severe nonproliferative diabetic retinopathy with macular edema, bilateral
**E10.3419**	Type 1 diabetes mellitus with severe nonproliferative diabetic retinopathy with macular edema, unspecified eye
**E10.3491**	Type 1 diabetes mellitus with severe nonproliferative diabetic retinopathy without macular edema, right eye
**E10.3492**	Type 1 diabetes mellitus with severe nonproliferative diabetic retinopathy without macular edema, left eye
**E10.3493**	Type 1 diabetes mellitus with severe nonproliferative diabetic retinopathy without macular edema, bilateral
**E10.3499**	Type 1 diabetes mellitus with severe nonproliferative diabetic retinopathy without macular edema, unspecified eye
**E10.3511**	Type 1 diabetes mellitus with proliferative diabetic retinopathy with macular edema, right eye

**E10.3512**	Type 1 diabetes mellitus with proliferative diabetic retinopathy with macular edema, left eye
**E10.3513**	Type 1 diabetes mellitus with proliferative diabetic retinopathy with macular edema, bilateral
**E10.3519**	Type 1 diabetes mellitus with proliferative diabetic retinopathy with macular edema, unspecified eye
**E10.3521**	Type 1 diabetes mellitus with proliferative diabetic retinopathy with traction retinal detachment involving the macula, right eye
**E10.3522**	Type 1 diabetes mellitus with proliferative diabetic retinopathy with traction retinal detachment involving the macula, left eye
**E10.3523**	Type 1 diabetes mellitus with proliferative diabetic retinopathy with traction retinal detachment involving the macula, bilateral
**E10.3529**	Type 1 diabetes mellitus with proliferative diabetic retinopathy with traction retinal detachment involving the macula, unspecified eye
**E10.3531**	Type 1 diabetes mellitus with proliferative diabetic retinopathy with traction retinal detachment not involving the macula, right eye
**E10.3532**	Type 1 diabetes mellitus with proliferative diabetic retinopathy with traction retinal detachment not involving the macula, left eye
**E10.3533**	Type 1 diabetes mellitus with proliferative diabetic retinopathy with traction retinal detachment not involving the macula, bilateral
**E10.3539**	Type 1 diabetes mellitus with proliferative diabetic retinopathy with traction retinal detachment not involving the macula, unspecified eye

**E10.3541**	Type 1 diabetes mellitus with proliferative diabetic retinopathy with combined traction retinal detachment and rhegmatogenous retinal detachment, right eye
**E10.3542**	Type 1 diabetes mellitus with proliferative diabetic retinopathy with combined traction retinal detachment and rhegmatogenous retinal detachment, left eye
**E10.3543**	Type 1 diabetes mellitus with proliferative diabetic retinopathy with combined traction retinal detachment and rhegmatogenous retinal detachment, bilateral
**E10.3549**	Type 1 diabetes mellitus with proliferative diabetic retinopathy with combined traction retinal detachment and rhegmatogenous retinal detachment, unspecified eye
**E10.3551**	Type 1 diabetes mellitus with stable proliferative diabetic retinopathy, right eye
**E10.3552**	Type 1 diabetes mellitus with stable proliferative diabetic retinopathy, left eye
**E10.3553**	Type 1 diabetes mellitus with stable proliferative diabetic retinopathy, bilateral
**E10.3559**	Type 1 diabetes mellitus with stable proliferative diabetic retinopathy, unspecified eye
**E10.3591**	Type 1 diabetes mellitus with proliferative diabetic retinopathy without macular edema, right eye
**E10.3592**	Type 1 diabetes mellitus with proliferative diabetic retinopathy without macular edema, left eye
**E10.3593**	Type 1 diabetes mellitus with proliferative diabetic retinopathy without macular edema, bilateral
**E10.3599**	Type 1 diabetes mellitus with proliferative diabetic retinopathy without macular edema, unspecified eye
**E10.36**	Type 1 diabetes mellitus with diabetic cataract
**E10.37X1**	Type 1 diabetes mellitus with diabetic macular edema, resolved following treatment, right eye

**E10.37X2**	Type 1 diabetes mellitus with diabetic macular edema, resolved following treatment, left eye
**E10.37X3**	Type 1 diabetes mellitus with diabetic macular edema, resolved following treatment, bilateral
**E10.37X9**	Type 1 diabetes mellitus with diabetic macular edema, resolved following treatment, unspecified eye
**E10.39**	Type 1 diabetes mellitus with other diabetic ophthalmic complication
**E10.40**	Type 1 diabetes mellitus with diabetic neuropathy, unspecified
**E10.41**	Type 1 diabetes mellitus with diabetic mononeuropathy
**E10.42**	Type 1 diabetes mellitus with diabetic polyneuropathy
**E10.43**	Type 1 diabetes mellitus with diabetic autonomic (poly)neuropathy
**E10.44**	Type 1 diabetes mellitus with diabetic amyotrophy
**E10.49**	Type 1 diabetes mellitus with other diabetic neurological complication
**E10.51**	Type 1 diabetes mellitus with diabetic peripheral angiopathy without gangrene
**E10.52**	Type 1 diabetes mellitus with diabetic peripheral angiopathy with gangrene
**E10.59**	Type 1 diabetes mellitus with other circulatory complications
**E10.610**	Type 1 diabetes mellitus with diabetic neuropathic arthropathy
**E10.618**	Type 1 diabetes mellitus with other diabetic arthropathy
**E10.620**	Type 1 diabetes mellitus with diabetic dermatitis
**E10.621**	Type 1 diabetes mellitus with foot ulcer

**E10.622**	Type 1 diabetes mellitus with other skin ulcer
**E10.628**	Type 1 diabetes mellitus with other skin complications
**E10.630**	Type 1 diabetes mellitus with periodontal disease
**E10.638**	Type 1 diabetes mellitus with other oral complications
**E10.641**	Type 1 diabetes mellitus with hypoglycemia with coma
**E10.649**	Type 1 diabetes mellitus with hypoglycemia without coma
**E10.65**	Type 1 diabetes mellitus with hyperglycemia
**E10.69**	Type 1 diabetes mellitus with other specified complication
**E10.8**	Type 1 diabetes mellitus with unspecified complications
**E10.9**	Type 1 diabetes mellitus without complications
**E10.10**	Type 1 diabetes mellitus with ketoacidosis without coma
**E10.11**	Type 1 diabetes mellitus with ketoacidosis with coma
**E10.641**	Type 1 diabetes mellitus with hypoglycemia with coma
**E11.00**	Type 2 diabetes mellitus with hyperosmolarity without nonketotic hyperglycemic-hyperosmolar coma (NKHHC)
**E11.01**	Type 2 diabetes mellitus with hyperosmolarity with coma
**E11.10**	Type 2 diabetes mellitus with ketoacidosis without coma
**E11.11**	Type 2 diabetes mellitus with ketoacidosis with coma

**E11.21**	Type 2 diabetes mellitus with diabetic nephropathy
**E11.29**	Type 2 diabetes mellitus with other diabetic kidney complication
**E11.311**	Type 2 diabetes mellitus with unspecified diabetic retinopathy with macular
**E11.319**	Type 2 diabetes mellitus with unspecified diabetic retinopathy without macular edema
**E11.36**	Type 2 diabetes mellitus with diabetic cataract
**E11.39**	Type 2 diabetes mellitus with other diabetic ophthalmic complication
**E11.40**	Type 2 diabetes mellitus with diabetic neuropathy, unspecified
**E11.51**	Type 2 diabetes mellitus with diabetic peripheral angiopathy without gangrene
**E11.618**	Type 2 diabetes mellitus with other diabetic arthropathy
**E11.620**	Type 2 diabetes mellitus with diabetic dermatitis
**E11.621**	Type 2 diabetes mellitus with foot ulcer
**E11.622**	Type 2 diabetes mellitus with other skin ulcer
**E11.628**	Type 2 diabetes mellitus with other skin complications
**E11.630**	Type 2 diabetes mellitus with periodontal disease
**E11.638**	Type 2 diabetes mellitus with other oral complications
**E11.641**	Type 2 diabetes mellitus with hypoglycemia with coma
**E11.649**	Type 2 diabetes mellitus with hypoglycemia without coma

**E11.65**	Type 2 diabetes mellitus with hyperglycemia
**E11.69**	Type 2 diabetes mellitus with other specified complication
**E11.8**	Type 2 diabetes mellitus with unspecified complications
**E11.9**	Type 2 diabetes mellitus without complications
**E13.10**	Other specified diabetes mellitus with ketoacidosis without coma

Table S5. List of ICD-10 CM codes used to define Smoking.

ICD-10 CM CODE	ICD-10 CM CODE DESCRIPTION
**Z72.0**	Tobacco use

Table S6. List of ICD-10 CM codes used to define coronary artery disease (CAD).

ICD-10 CM	ICD-10 CM DESCRIPTION
**I20.0**	Unstable angina
**I20.1**	Angina pectoris with documented spasm
**I20.8**	Other forms of angina pectoris
**I20.9**	Angina pectoris, unspecified
**I21.01**	ST elevation (STEMI) myocardial infarction involving left main coronary artery
**I21.02**	ST elevation (STEMI) myocardial infarction involving left anterior descending coronary artery
**I21.09**	ST elevation (STEMI) myocardial infarction involving other coronary artery of anterior wall
**I21.11**	ST elevation (STEMI) myocardial infarction involving right coronary artery
**I21.19**	ST elevation (STEMI) myocardial infarction involving other coronary artery of inferior wall
**I21.21**	ST elevation (STEMI) myocardial infarction involving left circumflex coronary artery
**I21.29**	ST elevation (STEMI) myocardial infarction involving other sites
**I21.3**	ST elevation (STEMI) myocardial infarction of unspecified site
**I21.4**	Non-ST elevation (NSTEMI) myocardial infarction
**I21.9**	Acute myocardial infarction, unspecified
**I21.A1**	Myocardial infarction type 2
**I21.A9**	Other myocardial infarction type
**I22.0**	Subsequent ST elevation (STEMI) myocardial infarction of anterior wall
**I22.1**	Subsequent ST elevation (STEMI) myocardial infarction of inferior wall
**I22.2**	Subsequent non-ST elevation (NSTEMI) myocardial infarction

**I22.8**	Subsequent ST elevation (STEMI) myocardial infarction of other sites
**I22.9**	Subsequent ST elevation (STEMI) myocardial infarction of unspecified site
**I23.0**	Hemopericardium as current complication following acute myocardial infarction
**I23.1**	Atrial septal defect as current complication following acute myocardial infarction
**I23.2**	Ventricular septal defect as current complication following acute myocardial infarction
**I23.3**	Rupture of cardiac wall without hemopericardium as current complication following acute myocardial infarction
**I23.4**	Rupture of chordae tendineae as current complication following acute myocardial infarction
**I23.5**	Rupture of papillary muscle as current complication following acute myocardial infarction
**I23.6**	Thrombosis of atrium, auricular appendage, and ventricle as current complications following acute myocardial infarction
**I23.7**	Postinfarction angina
**I23.8**	Other current complications following acute myocardial infarction
**I24.0**	Acute coronary thrombosis not resulting in myocardial infarction
**I24.1**	Dressler's syndrome
**I24.8**	Other forms of acute ischemic heart disease
**I24.9**	Acute ischemic heart disease, unspecified
**I25.10**	Atherosclerotic heart disease of native coronary artery without angina pectoris
**I25.110**	Atherosclerotic heart disease of native coronary artery with unstable angina pectoris
**I25.111**	Atherosclerotic heart disease of native coronary artery with angina pectoris with documented spasm
**I25.118**	Atherosclerotic heart disease of native coronary artery with other forms of angina pectoris
**I25.119**	Atherosclerotic heart disease of native coronary artery with unspecified angina pectoris
**I25.2**	Old myocardial infarction

**I25.3**	Aneurysm of heart
**I25.41**	Coronary artery aneurysm
**I25.42**	Coronary artery dissection
**I25.5**	Ischemic cardiomyopathy
**I25.6**	Silent myocardial ischemia
**I25.700**	Atherosclerosis of coronary artery bypass graft(s), unspecified, with unstable angina pectoris
**I25.701**	Atherosclerosis of coronary artery bypass graft(s), unspecified, with angina pectoris with documented spasm
**I25.708**	Atherosclerosis of coronary artery bypass graft(s), unspecified, with other forms of angina pectoris
**I25.709**	Atherosclerosis of coronary artery bypass graft(s), unspecified, with unspecified angina pectoris
**I25.710**	Atherosclerosis of autologous vein coronary artery bypass graft(s) with unstable angina pectoris
**I25.711**	Atherosclerosis of autologous vein coronary artery bypass graft(s) with angina pectoris with documented spasm
**I25.718**	Atherosclerosis of autologous vein coronary artery bypass graft(s) with other forms of angina pectoris
**I25.719**	Atherosclerosis of autologous vein coronary artery bypass graft(s) with unspecified angina pectoris
**I25.720**	Atherosclerosis of autologous artery coronary artery bypass graft(s) with unstable angina pectoris
**I25.721**	Atherosclerosis of autologous artery coronary artery bypass graft(s) with angina pectoris with documented spasm
**I25.728**	Atherosclerosis of autologous artery coronary artery bypass graft(s) with other forms of angina pectoris
**I25.729**	Atherosclerosis of autologous artery coronary artery bypass graft(s) with unspecified angina pectoris
**I25.730**	Atherosclerosis of nonautologous biological coronary artery bypass graft(s) with unstable angina pectoris
**I25.731**	Atherosclerosis of nonautologous biological coronary artery bypass graft(s) with angina pectoris with documented spasm
**I25.738**	Atherosclerosis of nonautologous biological coronary artery bypass graft(s) with other forms of angina pectoris
**I25.739**	Atherosclerosis of nonautologous biological coronary artery bypass graft(s) with unspecified angina pectoris

**I25.750**	Atherosclerosis of native coronary artery of transplanted heart with unstable angina
**I25.751**	Atherosclerosis of native coronary artery of transplanted heart with angina pectoris with documented spasm
**I25.758**	Atherosclerosis of native coronary artery of transplanted heart with other forms of angina pectoris
**I25.759**	Atherosclerosis of native coronary artery of transplanted heart with unspecified angina pectoris
**I25.760**	Atherosclerosis of bypass graft of coronary artery of transplanted heart with unstable angina
**I25.761**	Atherosclerosis of bypass graft of coronary artery of transplanted heart with angina pectoris with documented spasm
**I25.768**	Atherosclerosis of bypass graft of coronary artery of transplanted heart with other forms of angina pectoris
**I25.769**	Atherosclerosis of bypass graft of coronary artery of transplanted heart with unspecified angina pectoris
**I25.790**	Atherosclerosis of other coronary artery bypass graft(s) with unstable angina pectoris
**I25.791**	Atherosclerosis of other coronary artery bypass graft(s) with angina pectoris with documented spasm
**I25.798**	Atherosclerosis of other coronary artery bypass graft(s) with other forms of angina pectoris
**I25.799**	Atherosclerosis of other coronary artery bypass graft(s) with unspecified angina pectoris
**I25.810**	Atherosclerosis of coronary artery bypass graft(s) without angina pectoris
**I25.811**	Atherosclerosis of native coronary artery of transplanted heart without angina pectoris
**I25.812**	Atherosclerosis of bypass graft of coronary artery of transplanted heart without angina pectoris
**I25.82**	Chronic total occlusion of coronary artery
**I25.83**	Coronary atherosclerosis due to lipid rich plaque
**I25.84**	Coronary atherosclerosis due to calcified coronary lesion
**I25.89**	Other forms of chronic ischemic heart disease
**I25.9**	Chronic ischemic heart disease, unspecified
**T82.211A**	Breakdown (mechanical) of coronary artery bypass graft, initial encounter
**T82.212A**	Displacement of coronary artery bypass graft, initial encounter

**T82.213A**	Leakage of coronary artery bypass graft, initial encounter
**T82.218A**	Other mechanical complication of coronary artery bypass graft, initial encounter
**Z95.1**	Presence of aortocoronary bypass graft
**Z98.61**	Coronary angioplasty status

Table S7. List of ICD-10 CM codes used to define peripheral artery disease (PAD).

ICD-10 CM	ICD-10 CM DESCRIPTION
**I70.0**	Atherosclerosis of aorta
**I70.1**	Atherosclerosis of renal artery
**I70.201**	Unspecified atherosclerosis of native arteries of extremities, right leg
**I70.202**	Unspecified atherosclerosis of native arteries of extremities, left leg
**I70.203**	Unspecified atherosclerosis of native arteries of extremities, bilateral legs
**I70.208**	Unspecified atherosclerosis of native arteries of extremities, other extremity
**I70.209**	Unspecified atherosclerosis of native arteries of extremities, unspecified extremity
**I70.211**	Atherosclerosis of native arteries of extremities with intermittent claudication, right leg
**I70.212**	Atherosclerosis of native arteries of extremities with intermittent claudication, left leg
**I70.213**	Atherosclerosis of native arteries of extremities with intermittent claudication, bilateral legs
**I70.218**	Atherosclerosis of native arteries of extremities with intermittent claudication, other extremity
**I70.219**	Atherosclerosis of native arteries of extremities with intermittent claudication, unspecified extremity
**I70.221**	Atherosclerosis of native arteries of extremities with rest pain, right leg
**I70.222**	Atherosclerosis of native arteries of extremities with rest pain, left leg
**I70.223**	Atherosclerosis of native arteries of extremities with rest pain, bilateral legs

**I70.228**	Atherosclerosis of native arteries of extremities with rest pain, other extremity
**I70.229**	Atherosclerosis of native arteries of extremities with rest pain, unspecified extremity
**I70.231**	Atherosclerosis of native arteries of right leg with ulceration of thigh
**I70.232**	Atherosclerosis of native arteries of right leg with ulceration of calf
**I70.233**	Atherosclerosis of native arteries of right leg with ulceration of ankle
**I70.234**	Atherosclerosis of native arteries of right leg with ulceration of heel and midfoot
**I70.235**	Atherosclerosis of native arteries of right leg with ulceration of other part of foot
**I70.238**	Atherosclerosis of native arteries of right leg with ulceration of other part of lower leg
**I70.239**	Atherosclerosis of native arteries of right leg with ulceration of unspecified site
**I70.241**	Atherosclerosis of native arteries of left leg with ulceration of thigh
**I70.242**	Atherosclerosis of native arteries of left leg with ulceration of calf
**I70.243**	Atherosclerosis of native arteries of left leg with ulceration of ankle
**I70.244**	Atherosclerosis of native arteries of left leg with ulceration of heel and midfoot
**I70.245**	Atherosclerosis of native arteries of left leg with ulceration of other part of foot
**I70.248**	Atherosclerosis of native arteries of left leg with ulceration of other part of lower leg
**I70.249**	Atherosclerosis of native arteries of left leg with ulceration of unspecified site
**I70.25**	Atherosclerosis of native arteries of other extremities with ulceration

**I70.261**	Atherosclerosis of native arteries of extremities with gangrene, right leg
**I70.262**	Atherosclerosis of native arteries of extremities with gangrene, left leg
**I70.263**	Atherosclerosis of native arteries of extremities with gangrene, bilateral legs
**I70.268**	Atherosclerosis of native arteries of extremities with gangrene, other extremity
**I70.269**	Atherosclerosis of native arteries of extremities with gangrene, unspecified extremity
**I70.291**	Other atherosclerosis of native arteries of extremities, right leg
**I70.292**	Other atherosclerosis of native arteries of extremities, left leg
**I70.293**	Other atherosclerosis of native arteries of extremities, bilateral legs
**I70.298**	Other atherosclerosis of native arteries of extremities, other extremity
**I70.299**	Other atherosclerosis of native arteries of extremities, unspecified extremity
**I70.301**	Unspecified atherosclerosis of unspecified type of bypass graft(s) of the extremities, right leg
**I70.302**	Unspecified atherosclerosis of unspecified type of bypass graft(s) of the extremities, left leg
**I70.303**	Unspecified atherosclerosis of unspecified type of bypass graft(s) of the extremities, bilateral legs
**I70.308**	Unspecified atherosclerosis of unspecified type of bypass graft(s) of the extremities, other extremity
**I70.309**	Unspecified atherosclerosis of unspecified type of bypass graft(s) of the extremities, unspecified extremity
**I70.311**	Atherosclerosis of unspecified type of bypass graft(s) of the extremities with intermittent claudication, right leg
**I70.312**	Atherosclerosis of unspecified type of bypass graft(s) of the extremities with intermittent claudication, left leg

**I70.313**	Atherosclerosis of unspecified type of bypass graft(s) of the extremities with intermittent claudication, bilateral legs
**I70.318**	Atherosclerosis of unspecified type of bypass graft(s) of the extremities with intermittent claudication, other extremity
**I70.319**	Atherosclerosis of unspecified type of bypass graft(s) of the extremities with intermittent claudication, unspecified extremity
**I70.321**	Atherosclerosis of unspecified type of bypass graft(s) of the extremities with rest pain, right leg
**I70.322**	Atherosclerosis of unspecified type of bypass graft(s) of the extremities with rest pain, left leg
**I70.323**	Atherosclerosis of unspecified type of bypass graft(s) of the extremities with rest pain, bilateral legs
**I70.328**	Atherosclerosis of unspecified type of bypass graft(s) of the extremities with rest pain, other extremity
**I70.329**	Atherosclerosis of unspecified type of bypass graft(s) of the extremities with rest pain, unspecified extremity
**I70.331**	Atherosclerosis of unspecified type of bypass graft(s) of the right leg with ulceration of thigh
**I70.332**	Atherosclerosis of unspecified type of bypass graft(s) of the right leg with ulceration of calf
**I70.333**	Atherosclerosis of unspecified type of bypass graft(s) of the right leg with ulceration of ankle
**I70.334**	Atherosclerosis of unspecified type of bypass graft(s) of the right leg with ulceration of heel and midfoot
**I70.335**	Atherosclerosis of unspecified type of bypass graft(s) of the right leg with ulceration of other part of foot
**I70.338**	Atherosclerosis of unspecified type of bypass graft(s) of the right leg with ulceration of other part of lower leg
**I70.339**	Atherosclerosis of unspecified type of bypass graft(s) of the right leg with ulceration of unspecified site
**I70.341**	Atherosclerosis of unspecified type of bypass graft(s) of the left leg with ulceration of thigh

**I70.342**	Atherosclerosis of unspecified type of bypass graft(s) of the left leg with ulceration of calf
**I70.343**	Atherosclerosis of unspecified type of bypass graft(s) of the left leg with ulceration of ankle
**I70.344**	Atherosclerosis of unspecified type of bypass graft(s) of the left leg with ulceration of heel and midfoot
**I70.345**	Atherosclerosis of unspecified type of bypass graft(s) of the left leg with ulceration of other part of foot
**I70.348**	Atherosclerosis of unspecified type of bypass graft(s) of the left leg with ulceration of other part of lower leg
**I70.349**	Atherosclerosis of unspecified type of bypass graft(s) of the left leg with ulceration of unspecified site
**I70.35**	Atherosclerosis of unspecified type of bypass graft(s) of other extremity with ulceration
**I70.361**	Atherosclerosis of unspecified type of bypass graft(s) of the extremities with gangrene, right leg
**I70.362**	Atherosclerosis of unspecified type of bypass graft(s) of the extremities with gangrene, left leg
**I70.363**	Atherosclerosis of unspecified type of bypass graft(s) of the extremities with gangrene, bilateral legs
**I70.368**	Atherosclerosis of unspecified type of bypass graft(s) of the extremities with gangrene, other extremity
**I70.369**	Atherosclerosis of unspecified type of bypass graft(s) of the extremities with gangrene, unspecified extremity
**I70.391**	Other atherosclerosis of unspecified type of bypass graft(s) of the extremities, right leg
**I70.392**	Other atherosclerosis of unspecified type of bypass graft(s) of the extremities, left leg
**I70.393**	Other atherosclerosis of unspecified type of bypass graft(s) of the extremities, bilateral legs
**I70.398**	Other atherosclerosis of unspecified type of bypass graft(s) of the extremities, other extremity
**I70.399**	Other atherosclerosis of unspecified type of bypass graft(s) of the extremities, unspecified extremity

**I70.401**	Unspecified atherosclerosis of autologous vein bypass graft(s) of the extremities, right leg
**I70.402**	Unspecified atherosclerosis of autologous vein bypass graft(s) of the extremities, left leg
**I70.403**	Unspecified atherosclerosis of autologous vein bypass graft(s) of the extremities, bilateral legs
**I70.408**	Unspecified atherosclerosis of autologous vein bypass graft(s) of the extremities, other extremity
**I70.409**	Unspecified atherosclerosis of autologous vein bypass graft(s) of the extremities, unspecified extremity
**I70.411**	Atherosclerosis of autologous vein bypass graft(s) of the extremities with intermittent claudication, right leg
**I70.412**	Atherosclerosis of autologous vein bypass graft(s) of the extremities with intermittent claudication, left leg
**I70.413**	Atherosclerosis of autologous vein bypass graft(s) of the extremities with intermittent claudication, bilateral legs
**I70.418**	Atherosclerosis of autologous vein bypass graft(s) of the extremities with intermittent claudication, other extremity
**I70.419**	Atherosclerosis of autologous vein bypass graft(s) of the extremities with intermittent claudication, unspecified extremity
**I70.421**	Atherosclerosis of autologous vein bypass graft(s) of the extremities with rest pain, right leg
**I70.422**	Atherosclerosis of autologous vein bypass graft(s) of the extremities with rest pain, left leg
**I70.423**	Atherosclerosis of autologous vein bypass graft(s) of the extremities with rest pain, bilateral legs
**I70.428**	Atherosclerosis of autologous vein bypass graft(s) of the extremities with rest pain, other extremity
**I70.429**	Atherosclerosis of autologous vein bypass graft(s) of the extremities with rest pain, unspecified extremity
**I70.431**	Atherosclerosis of autologous vein bypass graft(s) of the right leg with ulceration of thigh

**I70.432**	Atherosclerosis of autologous vein bypass graft(s) of the right leg with ulceration of calf
**I70.433**	Atherosclerosis of autologous vein bypass graft(s) of the right leg with ulceration of ankle
**I70.434**	Atherosclerosis of autologous vein bypass graft(s) of the right leg with ulceration of heel and midfoot
**I70.435**	Atherosclerosis of autologous vein bypass graft(s) of the right leg with ulceration of other part of foot
**I70.438**	Atherosclerosis of autologous vein bypass graft(s) of the right leg with ulceration of other part of lower leg
**I70.439**	Atherosclerosis of autologous vein bypass graft(s) of the right leg with ulceration of unspecified site
**I70.441**	Atherosclerosis of autologous vein bypass graft(s) of the left leg with ulceration of thigh
**I70.442**	Atherosclerosis of autologous vein bypass graft(s) of the left leg with ulceration of calf
**I70.443**	Atherosclerosis of autologous vein bypass graft(s) of the left leg with ulceration of ankle
**I70.444**	Atherosclerosis of autologous vein bypass graft(s) of the left leg with ulceration of heel and midfoot
**I70.445**	Atherosclerosis of autologous vein bypass graft(s) of the left leg with ulceration of other part of foot
**I70.448**	Atherosclerosis of autologous vein bypass graft(s) of the left leg with ulceration of other part of lower leg
**I70.449**	Atherosclerosis of autologous vein bypass graft(s) of the left leg with ulceration of unspecified site
**I70.45**	Atherosclerosis of autologous vein bypass graft(s) of other extremity with ulceration
**I70.461**	Atherosclerosis of autologous vein bypass graft(s) of the extremities with gangrene, right leg
**I70.462**	Atherosclerosis of autologous vein bypass graft(s) of the extremities with gangrene, left leg
**I70.463**	Atherosclerosis of autologous vein bypass graft(s) of the extremities with gangrene, bilateral legs

**I70.468**	Atherosclerosis of autologous vein bypass graft(s) of the extremities with gangrene, other extremity
**I70.469**	Atherosclerosis of autologous vein bypass graft(s) of the extremities with gangrene, unspecified extremity
**I70.491**	Other atherosclerosis of autologous vein bypass graft(s) of the extremities, right leg
**I70.492**	Other atherosclerosis of autologous vein bypass graft(s) of the extremities, left leg
**I70.493**	Other atherosclerosis of autologous vein bypass graft(s) of the extremities, bilateral legs
**I70.498**	Other atherosclerosis of autologous vein bypass graft(s) of the extremities, other extremity
**I70.499**	Other atherosclerosis of autologous vein bypass graft(s) of the extremities, unspecified extremity
**I70.501**	Unspecified atherosclerosis of nonautologous biological bypass graft(s) of the extremities, right leg
**I70.502**	Unspecified atherosclerosis of nonautologous biological bypass graft(s) of the extremities, left leg
**I70.503**	Unspecified atherosclerosis of nonautologous biological bypass graft(s) of the extremities, bilateral legs
**I70.508**	Unspecified atherosclerosis of nonautologous biological bypass graft(s) of the extremities, other extremity
**I70.509**	Unspecified atherosclerosis of nonautologous biological bypass graft(s) of the extremities, unspecified extremity
**I70.511**	Atherosclerosis of nonautologous biological bypass graft(s) of the extremities with intermittent claudication, right leg
**I70.512**	Atherosclerosis of nonautologous biological bypass graft(s) of the extremities with intermittent claudication, left leg
**I70.513**	Atherosclerosis of nonautologous biological bypass graft(s) of the extremities with intermittent claudication, bilateral legs

**I70.518**	Atherosclerosis of nonautologous biological bypass graft(s) of the extremities with intermittent claudication, other extremity
**I70.519**	Atherosclerosis of nonautologous biological bypass graft(s) of the extremities with intermittent claudication, unspecified extremity
**I70.521**	Atherosclerosis of nonautologous biological bypass graft(s) of the extremities with rest pain, right leg
**I70.522**	Atherosclerosis of nonautologous biological bypass graft(s) of the extremities with rest pain, left leg
**I70.523**	Atherosclerosis of nonautologous biological bypass graft(s) of the extremities with rest pain, bilateral legs
**I70.528**	Atherosclerosis of nonautologous biological bypass graft(s) of the extremities with rest pain, other extremity
**I70.529**	Atherosclerosis of nonautologous biological bypass graft(s) of the extremities with rest pain, unspecified extremity
**I70.531**	Atherosclerosis of nonautologous biological bypass graft(s) of the right leg with ulceration of thigh
**I70.532**	Atherosclerosis of nonautologous biological bypass graft(s) of the right leg with ulceration of calf
**I70.533**	Atherosclerosis of nonautologous biological bypass graft(s) of the right leg with ulceration of ankle
**I70.534**	Atherosclerosis of nonautologous biological bypass graft(s) of the right leg with ulceration of heel and midfoot
**I70.535**	Atherosclerosis of nonautologous biological bypass graft(s) of the right leg with ulceration of other part of foot
**I70.538**	Atherosclerosis of nonautologous biological bypass graft(s) of the right leg with ulceration of other part of lower leg
**I70.539**	Atherosclerosis of nonautologous biological bypass graft(s) of the right leg with ulceration of unspecified site
**I70.541**	Atherosclerosis of nonautologous biological bypass graft(s) of the left leg with ulceration of thigh

**I70.542**	Atherosclerosis of nonautologous biological bypass graft(s) of the left leg with ulceration of calf
**I70.543**	Atherosclerosis of nonautologous biological bypass graft(s) of the left leg with ulceration of ankle
**I70.544**	Atherosclerosis of nonautologous biological bypass graft(s) of the left leg with ulceration of heel and midfoot
**I70.545**	Atherosclerosis of nonautologous biological bypass graft(s) of the left leg with ulceration of other part of foot
**I70.548**	Atherosclerosis of nonautologous biological bypass graft(s) of the left leg with ulceration of other part of lower leg
**I70.549**	Atherosclerosis of nonautologous biological bypass graft(s) of the left leg with ulceration of unspecified site
**I70.55**	Atherosclerosis of nonautologous biological bypass graft(s) of other extremity with ulceration
**I70.561**	Atherosclerosis of nonautologous biological bypass graft(s) of the extremities with gangrene, right leg
**I70.562**	Atherosclerosis of nonautologous biological bypass graft(s) of the extremities with gangrene, left leg
**I70.563**	Atherosclerosis of nonautologous biological bypass graft(s) of the extremities with gangrene, bilateral legs
**I70.568**	Atherosclerosis of nonautologous biological bypass graft(s) of the extremities with gangrene, other extremity
**I70.569**	Atherosclerosis of nonautologous biological bypass graft(s) of the extremities with gangrene, unspecified extremity
**I70.591**	Other atherosclerosis of nonautologous biological bypass graft(s) of the extremities, right leg
**I70.592**	Other atherosclerosis of nonautologous biological bypass graft(s) of the extremities, left leg
**I70.593**	Other atherosclerosis of nonautologous biological bypass graft(s) of the extremities, bilateral legs
**I70.598**	Other atherosclerosis of nonautologous biological bypass graft(s) of the extremities, other extremity
**I70.599**	Other atherosclerosis of nonautologous biological bypass graft(s) of the extremities, unspecified extremity

**I70.601**	Unspecified atherosclerosis of nonbiological bypass graft(s) of the extremities, right leg
**I70.602**	Unspecified atherosclerosis of nonbiological bypass graft(s) of the extremities, left leg
**I70.603**	Unspecified atherosclerosis of nonbiological bypass graft(s) of the extremities, bilateral legs
**I70.608**	Unspecified atherosclerosis of nonbiological bypass graft(s) of the extremities, other extremity
**I70.609**	Unspecified atherosclerosis of nonbiological bypass graft(s) of the extremities, unspecified extremity
**I70.611**	Atherosclerosis of nonbiological bypass graft(s) of the extremities with intermittent claudication, right leg
**I70.612**	Atherosclerosis of nonbiological bypass graft(s) of the extremities with intermittent claudication, left leg
**I70.613**	Atherosclerosis of nonbiological bypass graft(s) of the extremities with intermittent claudication, bilateral legs
**I70.618**	Atherosclerosis of nonbiological bypass graft(s) of the extremities with intermittent claudication, other extremity
**I70.619**	Atherosclerosis of nonbiological bypass graft(s) of the extremities with intermittent claudication, unspecified extremity
**I70.621**	Atherosclerosis of nonbiological bypass graft(s) of the extremities with rest pain, right leg
**I70.622**	Atherosclerosis of nonbiological bypass graft(s) of the extremities with rest pain, left leg
**I70.623**	Atherosclerosis of nonbiological bypass graft(s) of the extremities with rest pain, bilateral legs
**I70.628**	Atherosclerosis of nonbiological bypass graft(s) of the extremities with rest pain, other extremity
**I70.629**	Atherosclerosis of nonbiological bypass graft(s) of the extremities with rest pain, unspecified extremity
**I70.631**	Atherosclerosis of nonbiological bypass graft(s) of the right leg with ulceration of thigh
**I70.632**	Atherosclerosis of nonbiological bypass graft(s) of the right leg with ulceration of calf

**I70.633**	Atherosclerosis of nonbiological bypass graft(s) of the right leg with ulceration of ankle
**I70.634**	Atherosclerosis of nonbiological bypass graft(s) of the right leg with ulceration of heel and midfoot
**I70.635**	Atherosclerosis of nonbiological bypass graft(s) of the right leg with ulceration of other part of foot
**I70.638**	Atherosclerosis of nonbiological bypass graft(s) of the right leg with ulceration of other part of lower leg
**I70.639**	Atherosclerosis of nonbiological bypass graft(s) of the right leg with ulceration of unspecified site
**I70.641**	Atherosclerosis of nonbiological bypass graft(s) of the left leg with ulceration of thigh
**I70.642**	Atherosclerosis of nonbiological bypass graft(s) of the left leg with ulceration of calf
**I70.643**	Atherosclerosis of nonbiological bypass graft(s) of the left leg with ulceration of ankle
**I70.644**	Atherosclerosis of nonbiological bypass graft(s) of the left leg with ulceration of heel and midfoot
**I70.645**	Atherosclerosis of nonbiological bypass graft(s) of the left leg with ulceration of other part of foot
**I70.648**	Atherosclerosis of nonbiological bypass graft(s) of the left leg with ulceration of other part of lower leg
**I70.649**	Atherosclerosis of nonbiological bypass graft(s) of the left leg with ulceration of unspecified site
**I70.65**	Atherosclerosis of nonbiological bypass graft(s) of other extremity with ulceration
**I70.661**	Atherosclerosis of nonbiological bypass graft(s) of the extremities with gangrene, right leg
**I70.662**	Atherosclerosis of nonbiological bypass graft(s) of the extremities with gangrene, left leg
**I70.663**	Atherosclerosis of nonbiological bypass graft(s) of the extremities with gangrene, bilateral legs
**I70.668**	Atherosclerosis of nonbiological bypass graft(s) of the extremities with gangrene, other extremity

**I70.669**	Atherosclerosis of nonbiological bypass graft(s) of the extremities with gangrene, unspecified extremity
**I70.691**	Other atherosclerosis of nonbiological bypass graft(s) of the extremities, right leg
**I70.692**	Other atherosclerosis of nonbiological bypass graft(s) of the extremities, left leg
**I70.693**	Other atherosclerosis of nonbiological bypass graft(s) of the extremities, bilateral legs
**I70.698**	Other atherosclerosis of nonbiological bypass graft(s) of the extremities, other extremity
**I70.699**	Other atherosclerosis of nonbiological bypass graft(s) of the extremities, unspecified extremity
**I70.701**	Unspecified atherosclerosis of other type of bypass graft(s) of the extremities, right leg
**I70.702**	Unspecified atherosclerosis of other type of bypass graft(s) of the extremities, left leg
**I70.703**	Unspecified atherosclerosis of other type of bypass graft(s) of the extremities, bilateral legs
**I70.708**	Unspecified atherosclerosis of other type of bypass graft(s) of the extremities, other extremity
**I70.709**	Unspecified atherosclerosis of other type of bypass graft(s) of the extremities, unspecified extremity
**I70.711**	Atherosclerosis of other type of bypass graft(s) of the extremities with intermittent claudication, right leg
**I70.712**	Atherosclerosis of other type of bypass graft(s) of the extremities with intermittent claudication, left leg
**I70.713**	Atherosclerosis of other type of bypass graft(s) of the extremities with intermittent claudication, bilateral legs
**I70.718**	Atherosclerosis of other type of bypass graft(s) of the extremities with intermittent claudication, other extremity
**I70.719**	Atherosclerosis of other type of bypass graft(s) of the extremities with intermittent claudication, unspecified extremity
**I70.721**	Atherosclerosis of other type of bypass graft(s) of the extremities with rest pain, right leg

**I70.722**	Atherosclerosis of other type of bypass graft(s) of the extremities with rest pain, left leg
**I70.723**	Atherosclerosis of other type of bypass graft(s) of the extremities with rest pain, bilateral legs
**I70.728**	Atherosclerosis of other type of bypass graft(s) of the extremities with rest pain, other extremity
**I70.729**	Atherosclerosis of other type of bypass graft(s) of the extremities with rest pain, unspecified extremity
**I70.731**	Atherosclerosis of other type of bypass graft(s) of the right leg with ulceration of thigh
**I70.732**	Atherosclerosis of other type of bypass graft(s) of the right leg with ulceration of calf
**I70.733**	Atherosclerosis of other type of bypass graft(s) of the right leg with ulceration of ankle
**I70.734**	Atherosclerosis of other type of bypass graft(s) of the right leg with ulceration of heel and midfoot
**I70.735**	Atherosclerosis of other type of bypass graft(s) of the right leg with ulceration of other part of foot
**I70.738**	Atherosclerosis of other type of bypass graft(s) of the right leg with ulceration of other part of lower leg
**I70.739**	Atherosclerosis of other type of bypass graft(s) of the right leg with ulceration of unspecified site
**I70.741**	Atherosclerosis of other type of bypass graft(s) of the left leg with ulceration of thigh
**I70.742**	Atherosclerosis of other type of bypass graft(s) of the left leg with ulceration of calf
**I70.743**	Atherosclerosis of other type of bypass graft(s) of the left leg with ulceration of ankle
**I70.744**	Atherosclerosis of other type of bypass graft(s) of the left leg with ulceration of heel and midfoot
**I70.745**	Atherosclerosis of other type of bypass graft(s) of the left leg with ulceration of other part of foot
**I70.748**	Atherosclerosis of other type of bypass graft(s) of the left leg with ulceration of other part of lower leg

**I70.749**	Atherosclerosis of other type of bypass graft(s) of the left leg with ulceration of unspecified site
**I70.75**	Atherosclerosis of other type of bypass graft(s) of other extremity with ulceration
**I70.761**	Atherosclerosis of other type of bypass graft(s) of the extremities with gangrene, right leg
**I70.762**	Atherosclerosis of other type of bypass graft(s) of the extremities with gangrene, left leg
**I70.763**	Atherosclerosis of other type of bypass graft(s) of the extremities with gangrene, bilateral legs
**I70.768**	Atherosclerosis of other type of bypass graft(s) of the extremities with gangrene, other extremity
**I70.769**	Atherosclerosis of other type of bypass graft(s) of the extremities with gangrene, unspecified extremity
**I70.791**	Other atherosclerosis of other type of bypass graft(s) of the extremities, right leg
**I70.792**	Other atherosclerosis of other type of bypass graft(s) of the extremities, left leg
**I70.793**	Other atherosclerosis of other type of bypass graft(s) of the extremities, bilateral legs
**I70.798**	Other atherosclerosis of other type of bypass graft(s) of the extremities, other extremity
**I70.799**	Other atherosclerosis of other type of bypass graft(s) of the extremities, unspecified extremity
**I70.8**	Atherosclerosis of other arteries
**I70.90**	Unspecified atherosclerosis
**I70.91**	Generalized atherosclerosis
**I70.92**	Chronic total occlusion of artery of the extremities
**I73.89**	Other specified peripheral vascular diseases

**I73.9**	Peripheral vascular disease, unspecified

Table S8. List of ICD-10 CM codes used to define stroke.

ICD-10 CM	ICD-10 CM DESCRIPTION
**I60.0**	Nontraumatic subarachnoid hemorrhage from carotid siphon and bifurcation
**I60.00**	Nontraumatic subarachnoid hemorrhage from unspecified carotid siphon and bifurcation
**I60.01**	Nontraumatic subarachnoid hemorrhage from right carotid siphon and bifurcation
**I60.02**	Nontraumatic subarachnoid hemorrhage from left carotid siphon and bifurcation
**I60.1**	Nontraumatic subarachnoid hemorrhage from middle cerebral artery
**I60.10**	Nontraumatic subarachnoid hemorrhage from unspecified middle cerebral artery
**I60.11**	Nontraumatic subarachnoid hemorrhage from right middle cerebral artery
**I60.12**	Nontraumatic subarachnoid hemorrhage from left middle cerebral artery
**I60.2**	Nontraumatic subarachnoid hemorrhage from anterior communicating artery
**I60.3**	Nontraumatic subarachnoid hemorrhage from posterior communicating artery
**I60.30**	Nontraumatic subarachnoid hemorrhage from unspecified posterior communicating artery
**I60.31**	Nontraumatic subarachnoid hemorrhage from right posterior communicating artery
**I60.32**	Nontraumatic subarachnoid hemorrhage from left posterior communicating artery
**I60.4**	Nontraumatic subarachnoid hemorrhage from basilar artery
**I60.5**	Nontraumatic subarachnoid hemorrhage from vertebral artery

**I60.50**	Nontraumatic subarachnoid hemorrhage from unspecified vertebral artery
**I60.51**	Nontraumatic subarachnoid hemorrhage from right vertebral artery
**I60.52**	Nontraumatic subarachnoid hemorrhage from left vertebral artery
**I60.6**	Nontraumatic subarachnoid hemorrhage from other intracranial arteries
**I60.7**	Nontraumatic subarachnoid hemorrhage from unspecified intracranial artery
**I60.8**	Other nontraumatic subarachnoid hemorrhage
**I60.9**	Nontraumatic subarachnoid hemorrhage, unspecified
**I61.0**	Nontraumatic intracerebral hemorrhage in hemisphere, subcortical
**I61.1**	Nontraumatic intracerebral hemorrhage in hemisphere, cortical
**I61.2**	Nontraumatic intracerebral hemorrhage in hemisphere, unspecified
**I61.3**	Nontraumatic intracerebral hemorrhage in brain stem
**I61.4**	Nontraumatic intracerebral hemorrhage in cerebellum
**I61.5**	Nontraumatic intracerebral hemorrhage, intraventricular
**I61.6**	Nontraumatic intracerebral hemorrhage, multiple localized
**I61.8**	Other nontraumatic intracerebral hemorrhage
**I61.9**	Nontraumatic intracerebral hemorrhage, unspecified
**I62.00**	Nontraumatic subdural hemorrhage unspecified

**I62.01**	Nontraumatic acute subdural hemorrhage
**I62.02**	Nontraumatic subacute subdural hemorrhage
**I62.03**	Nontraumatic chronic subdural hemorrhage
**I62.1**	Nontraumatic extradural hemorrhage
**I62.9**	Nontraumatic intracranial hemorrhage, unspecified
**I63**	Cerebral infarction
**I63.0**	Cerebral infarction due to thrombosis of precerebral arteries
**I63.00**	Cerebral infarction due to thrombosis of unspecified precerebral artery
**I63.01**	Cerebral infarction due to thrombosis of vertebral artery
**I63.011**	Cerebral infarction due to thrombosis of right vertebral artery
**I63.012**	Cerebral infarction due to thrombosis of left vertebral artery
**I63.013**	Cerebral infarction due to thrombosis of bilateral vertebral arteries
**I63.019**	Cerebral infarction due to thrombosis of unspecified vertebral artery
**I63.02**	Cerebral infarction due to thrombosis of basilar artery
**I63.03**	Cerebral infarction due to thrombosis of carotid artery
**I63.031**	Cerebral infarction due to thrombosis of right carotid artery
**I63.032**	Cerebral infarction due to thrombosis of left carotid artery

**I63.033**	Cerebral infarction due to thrombosis of bilateral carotid arteries
**I63.039**	Cerebral infarction due to thrombosis of unspecified carotid artery
**I63.09**	Cerebral infarction due to thrombosis of other precerebral artery
**I63.1**	Cerebral infarction due to embolism of precerebral arteries
**I63.10**	Cerebral infarction due to embolism of unspecified precerebral artery
**I63.11**	Cerebral infarction due to embolism of vertebral artery
**I63.111**	Cerebral infarction due to embolism of right vertebral artery
**I63.112**	Cerebral infarction due to embolism of left vertebral artery
**I63.113**	Cerebral infarction due to embolism of bilateral vertebral arteries
**I63.119**	Cerebral infarction due to embolism of unspecified vertebral artery
**I63.12**	Cerebral infarction due to embolism of basilar artery
**I63.13**	Cerebral infarction due to embolism of carotid artery
**I63.131**	Cerebral infarction due to embolism of right carotid artery
**I63.132**	Cerebral infarction due to embolism of left carotid artery
**I63.133**	Cerebral infarction due to embolism of bilateral carotid arteries
**I63.139**	Cerebral infarction due to embolism of unspecified carotid artery
**I63.19**	Cerebral infarction due to embolism of other precerebral artery

**I63.2**	Cerebral infarction due to unspecified occlusion or stenosis of precerebral arteries
**I63.20**	Cerebral infarction due to unspecified occlusion or stenosis of unspecified precerebral arteries
**I63.211**	Cerebral infarction due to unspecified occlusion or stenosis of right vertebral artery
**I63.212**	Cerebral infarction due to unspecified occlusion or stenosis of left vertebral artery
**I63.213**	Cerebral infarction due to unspecified occlusion or stenosis of bilateral vertebral arteries
**I63.219**	Cerebral infarction due to unspecified occlusion or stenosis of unspecified vertebral artery
**I63.22**	Cerebral infarction due to unspecified occlusion or stenosis of basilar artery
**I63.231**	Cerebral infarction due to unspecified occlusion or stenosis of right carotid arteries
**I63.232**	Cerebral infarction due to unspecified occlusion or stenosis of left carotid arteries
**I63.233**	Cerebral infarction due to unspecified occlusion or stenosis of bilateral carotid arteries
**I63.239**	Cerebral infarction due to unspecified occlusion or stenosis of unspecified carotid artery
**I63.29**	Cerebral infarction due to unspecified occlusion or stenosis of other precerebral arteries
**I63.3**	Cerebral infarction due to thrombosis of cerebral arteries
**I63.30**	Cerebral infarction due to thrombosis of unspecified cerebral artery
**I63.31**	Cerebral infarction due to thrombosis of middle cerebral artery
**I63.311**	Cerebral infarction due to thrombosis of right middle cerebral artery
**I63.312**	Cerebral infarction due to thrombosis of left middle cerebral artery

**I63.313**	Cerebral infarction due to thrombosis of bilateral middle cerebral arteries
**I63.319**	Cerebral infarction due to thrombosis of unspecified middle cerebral artery
**I63.32**	Cerebral infarction due to thrombosis of anterior cerebral artery
**I63.321**	Cerebral infarction due to thrombosis of right anterior cerebral artery
**I63.322**	Cerebral infarction due to thrombosis of left anterior cerebral artery
**I63.323**	Cerebral infarction due to thrombosis of bilateral anterior cerebral arteries
**I63.329**	Cerebral infarction due to thrombosis of unspecified anterior cerebral artery
**I63.33**	Cerebral infarction due to thrombosis of posterior cerebral artery
**I63.331**	Cerebral infarction due to thrombosis of right posterior cerebral artery
**I63.332**	Cerebral infarction due to thrombosis of left posterior cerebral artery
**I63.333**	Cerebral infarction due to thrombosis of bilateral posterior cerebral arteries
**I63.339**	Cerebral infarction due to thrombosis of unspecified posterior cerebral artery
**I63.34**	Cerebral infarction due to thrombosis of cerebellar artery
**I63.341**	Cerebral infarction due to thrombosis of right cerebellar artery
**I63.342**	Cerebral infarction due to thrombosis of left cerebellar artery
**I63.343**	Cerebral infarction due to thrombosis of bilateral cerebellar arteries
**I63.349**	Cerebral infarction due to thrombosis of unspecified cerebellar artery

**I63.39**	Cerebral infarction due to thrombosis of other cerebral artery
**I63.4**	Cerebral infarction due to embolism of cerebral arteries
**I63.40**	Cerebral infarction due to embolism of unspecified cerebral artery
**I63.41**	Cerebral infarction due to embolism of middle cerebral artery
**I63.411**	Cerebral infarction due to embolism of right middle cerebral artery
**I63.412**	Cerebral infarction due to embolism of left middle cerebral artery
**I63.413**	Cerebral infarction due to embolism of bilateral middle cerebral arteries
**I63.419**	Cerebral infarction due to embolism of unspecified middle cerebral artery
**I63.42**	Cerebral infarction due to embolism of anterior cerebral artery
**I63.421**	Cerebral infarction due to embolism of right anterior cerebral artery
**I63.422**	Cerebral infarction due to embolism of left anterior cerebral artery
**I63.423**	Cerebral infarction due to embolism of bilateral anterior cerebral arteries
**I63.429**	Cerebral infarction due to embolism of unspecified anterior cerebral artery
**I63.43**	Cerebral infarction due to embolism of posterior cerebral artery
**I63.431**	Cerebral infarction due to embolism of right posterior cerebral artery
**I63.432**	Cerebral infarction due to embolism of left posterior cerebral artery
**I63.433**	Cerebral infarction due to embolism of bilateral posterior cerebral arteries

**I63.439**	Cerebral infarction due to embolism of unspecified posterior cerebral artery
**I63.44**	Cerebral infarction due to embolism of cerebellar artery
**I63.441**	Cerebral infarction due to embolism of right cerebellar artery
**I63.442**	Cerebral infarction due to embolism of left cerebellar artery
**I63.443**	Cerebral infarction due to embolism of bilateral cerebellar arteries
**I63.449**	Cerebral infarction due to embolism of unspecified cerebellar artery
**I63.49**	Cerebral infarction due to embolism of other cerebral artery
**I63.5**	Cerebral infarction due to unspecified occlusion or stenosis of cerebral arteries
**I63.50**	Cerebral infarction due to unspecified occlusion or stenosis of unspecified cerebral artery
**I63.511**	Cerebral infarction due to unspecified occlusion or stenosis of right middle cerebral artery
**I63.512**	Cerebral infarction due to unspecified occlusion or stenosis of left middle cerebral artery
**I63.513**	Cerebral infarction due to unspecified occlusion or stenosis of bilateral middle cerebral arteries
**I63.519**	Cerebral infarction due to unspecified occlusion or stenosis of unspecified middle cerebral artery
**I63.521**	Cerebral infarction due to unspecified occlusion or stenosis of right anterior cerebral artery
**I63.522**	Cerebral infarction due to unspecified occlusion or stenosis of left anterior cerebral artery
**I63.523**	Cerebral infarction due to unspecified occlusion or stenosis of bilateral anterior cerebral arteries
**I63.529**	Cerebral infarction due to unspecified occlusion or stenosis of unspecified anterior cerebral artery

**I63.531**	Cerebral infarction due to unspecified occlusion or stenosis of right posterior cerebral artery
**I63.532**	Cerebral infarction due to unspecified occlusion or stenosis of left posterior cerebral artery
**I63.533**	Cerebral infarction due to unspecified occlusion or stenosis of bilateral posterior cerebral arteries
**I63.539**	Cerebral infarction due to unspecified occlusion or stenosis of unspecified posterior cerebral artery
**I63.541**	Cerebral infarction due to unspecified occlusion or stenosis of right cerebellar artery
**I63.542**	Cerebral infarction due to unspecified occlusion or stenosis of left cerebellar artery
**I63.543**	Cerebral infarction due to unspecified occlusion or stenosis of bilateral cerebellar arteries
**I63.549**	Cerebral infarction due to unspecified occlusion or stenosis of unspecified cerebellar artery
**I63.59**	Cerebral infarction due to unspecified occlusion or stenosis of other cerebral artery
**I63.6**	Cerebral infarction due to cerebral venous thrombosis, nonpyogenic
**I63.8**	Other cerebral infarction
**I63.81**	Other cerebral infarction due to occlusion or stenosis of small artery
**I63.89**	Other cerebral infarction
**I63.9**	Cerebral infarction, unspecified
**I65.01**	Occlusion and stenosis of right vertebral artery
**I65.02**	Occlusion and stenosis of left vertebral artery
**I65.03**	Occlusion and stenosis of bilateral vertebral arteries

**I65.09**	Occlusion and stenosis of unspecified vertebral artery
**I65.1**	Occlusion and stenosis of basilar artery without mention of cerebral infarction
**I65.21**	Occlusion and stenosis of right carotid artery
**I65.22**	Occlusion and stenosis of left carotid artery
**I65.23**	Occlusion and stenosis of bilateral carotid arteries
**I65.29**	Occlusion and stenosis of unspecified carotid artery
**I65.8**	Occlusion and stenosis of other precerebral arteries
**I65.9**	Occlusion and stenosis of unspecified precerebral artery
**I66.01**	Occlusion and stenosis of right middle cerebral artery
**I66.02**	Occlusion and stenosis of left middle cerebral artery
**I66.03**	Occlusion and stenosis of both middle cerebral arteries
**I66.09**	Occlusion and stenosis of middle cerebral artery
**I66.11**	Occlusion and stenosis of right anterior cerebral artery
**I66.12**	Occlusion and stenosis of left anterior cerebral artery
**I66.13**	Occlusion and stenosis of both anterior cerebral arteries
**I66.19**	Occlusion and stenosis of anterior cerebral artery
**I66.21**	Occlusion and stenosis of right posterior cerebral artery

**I66.22**	Occlusion and stenosis of left posterior cerebral artery
**I66.23**	Occlusion and stenosis of both posterior cerebral arteries
**I66.29**	Occlusion and stenosis of posterior cerebral artery
**I66.3**	Thromboembolism in extracranial artery
**I66.8**	Occlusion and stenosis of other cerebral arteries
**I66.9**	Cerebral thrombosis without mention of cerebral infarction
**I67**	Other cerebrovascular diseases
**I67.2**	Cerebral atherosclerosis
**I67.8**	Other specified cerebrovascular diseases
**I67.848**	Other cerebrovascular vasospasm and vasoconstriction
**I67.850**	Cerebral autosomal dominant arteriopathy with subcortical infarcts and leukoencephalopathy
**I67.858**	Other hereditary cerebrovascular disease
**I67.89**	Other cerebrovascular disease
**I67.9**	Cerebrovascular disease, unspecified
**I68.8**	Other cerebrovascular disorders in diseases classified elsewhere
**I69**	Sequelae of cerebrovascular disease
**I69.3**	Sequelae of cerebral infarction

**I69.30**	Unspecified sequelae of cerebral infarction
**I69.31**	Cognitive deficits following cerebral infarction
**I69.310**	Attention and concentration deficit following cerebral infarction
**I69.311**	Memory deficit following cerebral infarction
**I69.312**	Visuospatial deficit and spatial neglect following cerebral infarction
**I69.313**	Psychomotor deficit following cerebral infarction
**I69.314**	Frontal lobe and executive function deficit following cerebral infarction
**I69.315**	Cognitive social or emotional deficit following cerebral infarction
**I69.318**	Other symptoms and signs involving cognitive functions following cerebral infarction
**I69.319**	Unspecified symptoms and signs involving cognitive functions following cerebral infarction
**I69.320**	Aphasia following cerebral infarction
**I69.321**	Dysphasia following cerebral infarction
**I69.322**	Dysarthria following cerebral infarction
**I69.323**	Fluency disorder following cerebral infarction
**I69.328**	Other speech and language deficits following cerebral infarction
**I69.331**	Monoplegia of upper limb following cerebral infarction affecting right dominant side
**I69.332**	Monoplegia of upper limb following cerebral infarction affecting left dominant side

**I69.333**	Monoplegia of upper limb following cerebral infarction affecting right non-dominant side
**I69.334**	Monoplegia of upper limb following cerebral infarction affecting left non-dominant side
**I69.339**	Monoplegia of upper limb following cerebral infarction affecting unspecified side
**I69.341**	Monoplegia of lower limb following cerebral infarction affecting right dominant side
**I69.342**	Monoplegia of lower limb following cerebral infarction affecting left dominant side
**I69.343**	Monoplegia of lower limb following cerebral infarction affecting right non-dominant side
**I69.344**	Monoplegia of lower limb following cerebral infarction affecting left non-dominant side
**I69.349**	Monoplegia of lower limb following cerebral infarction affecting unspecified side
**I69.35**	Hemiplegia and hemiparesis following cerebral infarction
**I69.351**	Hemiplegia and hemiparesis following cerebral infarction affecting right dominant side
**I69.352**	Hemiplegia and hemiparesis following cerebral infarction affecting left dominant side
**I69.353**	Hemiplegia and hemiparesis following cerebral infarction affecting right non-dominant side
**I69.354**	Hemiplegia and hemiparesis following cerebral infarction affecting left non-dominant side
**I69.359**	Hemiplegia and hemiparesis following cerebral infarction affecting unspecified side
**I69.36**	Other paralytic syndrome following cerebral infarction
**I69.361**	Other paralytic syndrome following cerebral infarction affecting right dominant side
**I69.362**	Other paralytic syndrome following cerebral infarction affecting left dominant side

**I69.363**	Other paralytic syndrome following cerebral infarction affecting right non-dominant side
**I69.364**	Other paralytic syndrome following cerebral infarction affecting left non-dominant side
**I69.365**	Other paralytic syndrome following cerebral infarction, bilateral
**I69.369**	Other paralytic syndrome following cerebral infarction affecting unspecified side
**I69.39**	Other sequelae of cerebral infarction
**I69.390**	Apraxia following cerebral infarction
**I69.391**	Dysphagia following cerebral infarction
**I69.392**	Facial weakness following cerebral infarction
**I69.393**	Ataxia following cerebral infarction
**I69.398**	Other sequelae of cerebral infarction
**I69.80**	Unspecified sequelae of other cerebrovascular disease
**I69.810**	Attention and concentration deficit following other cerebrovascular disease
**I69.811**	Memory deficit following other cerebrovascular disease
**I69.812**	Visuospatial deficit and spatial neglect following other cerebrovascular disease
**I69.813**	Psychomotor deficit following other cerebrovascular disease
**I69.814**	Frontal lobe and executive function deficit following other cerebrovascular disease
**I69.815**	Cognitive social or emotional deficit following other cerebrovascular disease

**I69.818**	Other symptoms and signs involving cognitive functions following other cerebrovascular disease
**I69.819**	Unspecified symptoms and signs involving cognitive functions following other cerebrovascular disease
**I69.820**	Aphasia following other cerebrovascular disease
**I69.821**	Dysphasia following other cerebrovascular disease
**I69.822**	Dysarthria following other cerebrovascular disease
**I69.823**	Fluency disorder following other cerebrovascular disease
**I69.828**	Other speech and language deficits following other cerebrovascular disease
**I69.831**	Monoplegia of upper limb following other cerebrovascular disease affecting right dominant side
**I69.832**	Monoplegia of upper limb following other cerebrovascular disease affecting left dominant side
**I69.833**	Monoplegia of upper limb following other cerebrovascular disease affecting right non-dominant side
**I69.834**	Monoplegia of upper limb following other cerebrovascular disease affecting left non-dominant side
**I69.839**	Monoplegia of upper limb following other cerebrovascular disease affecting unspecified side
**I69.841**	Monoplegia of lower limb following other cerebrovascular disease affecting right dominant side
**I69.842**	Monoplegia of lower limb following other cerebrovascular disease affecting left dominant side
**I69.843**	Monoplegia of lower limb following other cerebrovascular disease affecting right non-dominant side
**I69.844**	Monoplegia of lower limb following other cerebrovascular disease affecting left non-dominant side
**I69.849**	Monoplegia of lower limb following other cerebrovascular disease affecting unspecified side

**I69.85**	Hemiplegia and hemiparesis following other cerebrovascular disease
**I69.851**	Hemiplegia and hemiparesis following other cerebrovascular disease affecting right dominant side
**I69.852**	Hemiplegia and hemiparesis following other cerebrovascular disease affecting left dominant side
**I69.853**	Hemiplegia and hemiparesis following other cerebrovascular disease affecting right non-dominant side
**I69.854**	Hemiplegia and hemiparesis following other cerebrovascular disease affecting left non-dominant side
**I69.859**	Hemiplegia and hemiparesis following other cerebrovascular disease affecting unspecified side
**I69.86**	Other paralytic syndrome following other cerebrovascular disease
**I69.861**	Other paralytic syndrome following other cerebrovascular disease affecting right dominant side
**I69.862**	Other paralytic syndrome following other cerebrovascular disease affecting left dominant side
**I69.863**	Other paralytic syndrome following other cerebrovascular disease affecting right non-dominant side
**I69.864**	Other paralytic syndrome following other cerebrovascular disease affecting left non-dominant side
**I69.865**	Other paralytic syndrome following other cerebrovascular disease, bilateral
**I69.869**	Other paralytic syndrome following other cerebrovascular disease affecting unspecified side
**I69.89**	Other sequelae of other cerebrovascular disease
**I69.890**	Apraxia following other cerebrovascular disease
**I69.891**	Dysphagia following other cerebrovascular disease
**I69.892**	Facial weakness following other cerebrovascular disease

**I69.893**	Ataxia following other cerebrovascular disease
**I69.898**	Other sequelae of other cerebrovascular disease
**I69.90**	Unspecified sequelae of unspecified cerebrovascular disease
**I69.91**	Cognitive deficits following unspecified cerebrovascular disease
**I69.910**	Attention and concentration deficit following unspecified cerebrovascular disease
**I69.911**	Memory deficit following unspecified cerebrovascular disease
**I69.912**	Visuospatial deficit and spatial neglect following unspecified cerebrovascular disease
**I69.913**	Psychomotor deficit following unspecified cerebrovascular disease
**I69.914**	Frontal lobe and executive function deficit following unspecified cerebrovascular disease
**I69.915**	Cognitive social or emotional deficit following unspecified cerebrovascular disease
**I69.918**	Other symptoms and signs involving cognitive functions following unspecified cerebrovascular disease
**I69.919**	Unspecified symptoms and signs involving cognitive functions following unspecified cerebrovascular disease
**I69.920**	Aphasia following unspecified cerebrovascular disease
**I69.921**	Dysphasia following unspecified cerebrovascular disease
**I69.922**	Dysarthria following unspecified cerebrovascular disease
**I69.923**	Fluency disorder following unspecified cerebrovascular disease
**I69.928**	Other speech and language deficits following unspecified cerebrovascular disease

**I69.931**	Monoplegia of upper limb following unspecified cerebrovascular disease affecting right dominant side
**I69.932**	Monoplegia of upper limb following unspecified cerebrovascular disease affecting left dominant side
**I69.933**	Monoplegia of upper limb following unspecified cerebrovascular disease affecting right non-dominant side
**I69.934**	Monoplegia of upper limb following unspecified cerebrovascular disease affecting left non-dominant side
**I69.939**	Monoplegia of upper limb following unspecified cerebrovascular disease affecting unspecified side
**I69.941**	Monoplegia of lower limb following unspecified cerebrovascular disease affecting right dominant side
**I69.942**	Monoplegia of lower limb following unspecified cerebrovascular disease affecting left dominant side
**I69.943**	Monoplegia of lower limb following unspecified cerebrovascular disease affecting right non-dominant side
**I69.944**	Monoplegia of lower limb following unspecified cerebrovascular disease affecting left non-dominant side
**I69.949**	Monoplegia of lower limb following unspecified cerebrovascular disease affecting unspecified side
**I69.951**	Hemiplegia and hemiparesis following unspecified cerebrovascular disease affecting right dominant side
**I69.952**	Hemiplegia and hemiparesis following unspecified cerebrovascular disease affecting left dominant side
**I69.953**	Hemiplegia and hemiparesis following unspecified cerebrovascular disease affecting right non-dominant side
**I69.954**	Hemiplegia and hemiparesis following unspecified cerebrovascular disease affecting left non-dominant side
**I69.959**	Hemiplegia and hemiparesis following unspecified cerebrovascular disease affecting unspecified side
**I69.961**	Other paralytic syndrome following unspecified cerebrovascular disease affecting right dominant side
**I69.962**	Other paralytic syndrome following unspecified cerebrovascular disease affecting left dominant side

**I69.963**	Other paralytic syndrome following unspecified cerebrovascular disease affecting right non-dominant side
**I69.964**	Other paralytic syndrome following unspecified cerebrovascular disease affecting left non-dominant side
**I69.969**	Other paralytic syndrome following unspecified cerebrovascular disease affecting unspecified side
**I69.990**	Apraxia following unspecified cerebrovascular disease
**I69.991**	Dysphagia following unspecified cerebrovascular disease
**I69.992**	Facial weakness following unspecified cerebrovascular disease
**I69.993**	Ataxia following unspecified cerebrovascular disease
**I69.998**	Other sequelae following unspecified cerebrovascular disease
**I97.81**	Intraoperative cerebrovascular infarction
**I97.810**	Intraoperative cerebrovascular infarction during cardiac surgery
**I97.811**	Intraoperative cerebrovascular infarction during other surgery
**I97.820**	Postprocedural cerebrovascular infarction following cardiac surgery
**I97.821**	Postprocedural cerebrovascular infarction following other surgery
**Z86.73**	Personal history of transient ischemic attack (TIA), and cerebral infarction without residual deficits

Table S9. Demographics and prevalence of cardiovascular risk factors, stratified by ASCVD status.

Characteristics	Total	Nature Deficient / Nature Light (0-39)	Nature Adequate (40-59)	Nature Rich (60-79)	Nature Utopia (80-100)	p-value#
**No. of patients**	1,077,181	160,818 (15%)	224,163 (21%)	351,942 (33%)	340,258 (32%)	
**ASCVD population**						
**No. of patients**	102,238	12,544 (12%)	20,456 (20%)	33,638 (33%)	35,600 (35%)	
**NatureScore, Median (IQR)**	72 (54-85)	28 (19-35)	51 (46-56)	71 (66-75)	89 (84-93)	<0.001
**Sex**						<0.001
**Male**	55,725 (55%)	6,616 (53%)	10,885 (53%)	18,177 (54%)	20,047 (56%)	
**Female**	46,513 (45%)	5,928 (47%)	9,571 (47%)	15,461 (46%)	15,553 (44%)	
**Age, median (IQR)**	70 (62-78)	70 (61-78)	70 (61-77)	71 (62-78)	71 (63-78)	<0.001
**Age by group**						<0.001
**18-39**	1,919 (2%)	324 (3%)	429 (2%)	623 (2%)	543 (2%)	

**40-64**	29,291 (29%)	3,866 (31%)	6,201 (30%)	9,397 (28%)	9,827 (28%)	
**65-79**	50,851 (50%)	5,843 (47%)	9,837 (48%)	16,983 (50%)	18,188 (51%)	
**80+**	20,177 (20%)	2,511 (20%)	3,989 (20%)	6,635 (20%)	7,042 (20%)	
**Race/Ethnicity**						<0.001
**Hispanic (H)**	12,034 (12%)	1,892 (15%)	2,859 (14%)	4,134 (12%)	3,149 (9%)	
**Non-H White**	63,928 (63%)	6,897 (55%)	11,076 (54%)	19,734 (59%)	26,221 (74%)	
**Non-H Black**	16,239 (16%)	2,335 (19%)	4,088 (20%)	6,391 (19%)	3,425 (10%)	
**Non-H Asian**	6,452 (6%)	917 (7%)	1,683 (8%)	2,241 (7%)	1,611 (5%)	
**Others**	3,585 (4%)	503 (4%)	750 (4%)	1,138 (3%)	1,194 (3%)	
**ADI National**						<0.001
**1st Quintile (Least deprived)**	20,319 (20%)	3,470 (28%)	4,291 (21%)	5,905 (18%)	6,653 (19%)	
**2nd Quintile**	29,073 (28%)	2,500 (20%)	4,950 (24%)	10,272 (31%)	11,351 (32%)	
**3rd Quintile**	24,633 (24%)	2,411 (19%)	4,704 (23%)	8,049 (24%)	9,469 (27%)	
**4th Quintile**	18,106 (18%)	2,476 (20%)	4,054 (20%)	6,089 (18%)	5,487 (15%)	

**5th Quintile (Most deprived)**	9,867 (10%)	1,615 (13%)	2,410 (12%)	3,256 (10%)	2,586 (7%)	
**Unknown**	240 (0%)	72 (1%)	47 (0%)	67 (0%)	54 (0%)	
**BMI**						<0.001
**Underweight (<18.5)**	2,100 (2%)	301 (2%)	413 (2%)	681 (2%)	705 (2%)	
**Normal (18.5-24.9)**	25,345 (25%)	3,331 (27%)	5,223 (26%)	8,211 (24%)	8,580 (24%)	
**Overweight (25-29.9)**	34,012 (33%)	4,133 (33%)	6,698 (33%)	11,179 (33%)	12,002 (34%)	
**Obesity class I (30-34.9)**	22,007 (22%)	2,521 (20%)	4,340 (21%)	7,268 (22%)	7,878 (22%)	
**Obesity Class II (35-39.9)**	9,660 (9%)	1,069 (9%)	1,850 (9%)	3,200 (10%)	3,541 (10%)	
**Obesity Class III (40+)**	5,926 (6%)	694 (6%)	1,240 (6%)	2,049 (6%)	1,943 (5%)	
**Unknown**	3,188 (3%)	495 (4%)	692 (3%)	1,050 (3%)	951 (3%)	
**CV Risk Factors**						

**Any CV Risk**	98,886 (97%)	12,060 (96%)	19,791 (97%)	32,584 (97%)	34,451 (97%)	0.001
**Hypertension**	91,373 (89%)	11,011 (88%)	18,289 (89%)	30,180 (90%)	31,893 (90%)	<0.001
**Diabetes Mellitus**	44,752 (44%)	5,467 (44%)	9,285 (45%)	15,099 (45%)	14,901 (42%)	<0.001
**Dyslipidemia**	57,751 (56%)	6,937 (55%)	11,422 (56%)	19,142 (57%)	20,250 (57%)	0.002
**Obesity**	37,593 (37%)	4,284 (34%)	7,430 (36%)	12,517 (37%)	13,362 (38%)	<0.001
**Smoking Ever**	64,809 (63%)	7,796 (62%)	12,992 (64%)	21,449 (64%)	22,572 (63%)	0.015
**Non-ASCVD population**						
**No. of patients**	974,943	148,274 (15%)	203,707 (21%)	318,304 (33%)	304,658 (31%)	
**NatureScore, Median (IQR)**	69 (51-83)	27 (17-34)	51 (46-56)	71 (66-75)	88 (84-93)	<0.001
**Sex**						<0.001

**Male**	386,792 (40%)	60,053 (41%)	79,903 (39%)	124,519 (39%)	122,317 (40%)	
**Female**	588,151 (60%)	88,221 (59%)	123,804 (61%)	193,785 (61%)	182,341 (60%)	
**Age, median (IQR)**	50 (36-64)	44 (32-61)	48 (35-63)	50 (36-65)	53 (38-66)	<0.001
**Age by group**						<0.001
**18-39**	312,857 (32%)	61,991 (42%)	69,159 (34%)	98,663 (31%)	83,044 (27%)	
**40-64**	421,000 (43%)	56,050 (38%)	87,516 (43%)	140,029 (44%)	137,405 (45%)	
**65-79**	192,909 (20%)	23,772 (16%)	37,634 (18%)	63,490 (20%)	68,013 (22%)	
**80+**	48,177 (5%)	6,461 (4%)	9,398 (5%)	16,122 (5%)	16,196 (5%)	
**Race/Ethnicity**						<0.001
**Hispanic (H)**	160,032 (16%)	26,753 (18%)	37,825 (19%)	55,008 (17%)	40,446 (13%)	
**Non-H White**	518,626 (53%)	67,742 (46%)	92,628 (45%)	161,367 (51%)	196,889 (65%)	

**Non-H Black**	139,527 (14%)	24,236 (16%)	36,331 (18%)	51,454 (16%)	27,506 (9%)	
**Non-H Asian**	74,002 (8%)	13,940 (9%)	19,112 (9%)	24,129 (8%)	16,821 (6%)	
**Others**	82,756 (8%)	15,603 (11%)	17,811 (9%)	26,346 (8%)	22,996 (8%)	
**ADI National**						<0.001
**1st Quintile (Least deprived)**	232,849 (24%)	48,644 (33%)	49,695 (24%)	66,758 (21%)	67,752 (22%)	
**2nd Quintile**	290,652 (30%)	30,829 (21%)	55,081 (27%)	103,004 (32%)	101,738 (33%)	
**3rd Quintile**	234,882 (24%)	29,520 (20%)	48,386 (24%)	77,885 (24%)	79,091 (26%)	
**4th Quintile**	147,304 (15%)	25,822 (17%)	33,619 (17%)	49,083 (15%)	38,780 (13%)	
**5th Quintile (Most deprived)**	66,373 (7%)	12,381 (8%)	16,565 (8%)	20,762 (7%)	16,665 (5%)	
**Unknown**	2,883 (0%)	1,078 (1%)	361 (0%)	812 (0%)	632 (0%)	
**BMI**						<0.001

**Underweight (<18.5)**	15,121 (2%)	2,578 (2%)	3,100 (2%)	4,777 (2%)	4,666 (2%)	
**Normal (18.5-24.9)**	245,189 (25%)	41,099 (28%)	50,537 (25%)	77,946 (24%)	75,607 (25%)	
**Overweight (25-29.9)**	280,418 (29%)	40,915 (28%)	57,630 (28%)	91,593 (29%)	90,280 (30%)	
**Obesity class I (30-34.9)**	177,772 (18%)	22,813 (15%)	37,221 (18%)	59,642 (19%)	58,096 (19%)	
**Obesity Class II (35-39.9)**	84,951 (9%)	10,616 (7%)	17,775 (9%)	28,924 (9%)	27,636 (9%)	
**Obesity Class III (40+)**	61,852 (6%)	8,383 (6%)	13,237 (6%)	21,067 (7%)	19,165 (6%)	
**Unknown**	109,640 (11%)	21,870 (15%)	24,207 (12%)	34,355 (11%)	29,208 (10%)	
**CV Risk Factors**						
**Any CV Risk**	634,328 (65%)	86,280 (58%)	131,710 (65%)	210,825 (66%)	205,513 (67%)	<0.001
**Hypertension**	357,979 (37%)	45,622 (31%)	73,104 (36%)	120,034 (38%)	119,219 (39%)	<0.001

**Diabetes Mellitus**	134,497 (14%)	17,300 (12%)	28,721 (14%)	46,106 (14%)	42,370 (14%)	<0.001
**Dyslipidemia**	258,353 (26%)	35,350 (24%)	53,270 (26%)	85,187 (27%)	84,546 (28%)	<0.001
**Obesity**	324,575 (33%)	41,812 (28%)	68,233 (33%)	109,633 (34%)	104,897 (34%)	<0.001
**Smoking Ever**	229,219 (24%)	29,719 (20%)	47,377 (23%)	76,624 (24%)	75,499 (25%)	<0.001

ADI: area deprivation index; ASCVD: atherosclerotic cardiovascular disease; BMI: body mass index; CV: cardiovascular; CAD: coronary artery disease; IQR: inter-quartile range; PAD: peripheral artery disease; [#]Chi-square test and Kruskal-Wallis test conducted to compare the four categories of NatureScore for categorical and continuous non-normally distributed variables, respectively.

Table S10. Prevalence of cardiovascular diseases and cardiovascular risk factors stratified by walkability (n=1,077,181).

Characteristics	Total Population	NatureScore Groups				
		Nature Deficient / Nature Light (0-39)	Nature Adequate (40-59)	Nature Rich (60-79)	Nature Utopia (80-100)	p-value[#]
**Car Dependent (All Errands)**						
**Overall Population**	436,293	19,605 (4%)	77,048 (18%)	154,279 (35%)	185,361 (42%)	
**CV Risk Factors**						
**Any CV Risk**	304,316 (70%)	13,558 (69%)	53,367 (69%)	107,275 (70%)	130,116 (70%)	<0.001
**Hypertension**	184,522 (42%)	7,875 (40%)	31,218 (41%)	64,651 (42%)	80,778 (44%)	<0.001
**Diabetes Mellitus**	71,516 (16%)	3,284 (17%)	12,473 (16%)	25,530 (17%)	30,229 (16%)	0.049
**Dyslipidemia**	136,358 (31%)	5,679 (29%)	23,762 (31%)	48,272 (31%)	58,645 (32%)	<0.001
**Obesity**	154,786 (35%)	7,364 (38%)	28,058 (36%)	55,017 (36%)	64,347 (35%)	<0.001
**Smoking Ever**	118,428 (27%)	5,204 (27%)	20,145 (26%)	41,451 (27%)	51,628 (28%)	<0.001
**ASCVD**						
**Any ASCVD**	42,232 (10%)	1,611 (8%)	6,826 (9%)	14,529 (9%)	19,266 (10%)	<0.001

**CAD**	28,461 (7%)	1,081 (6%)	4,523 (6%)	9,746 (6%)	13,111 (7%)	<0.001
**PAD**	8,490 (2%)	332 (2%)	1,341 (2%)	2,964 (2%)	3,853 (2%)	<0.001
**Stroke**	15,224 (3%)	572 (3%)	2,487 (3%)	5,319 (3%)	6,846 (4%)	<0.001
**Car Dependent (Most Errands)**						
**Overall Population**	377,466	54,162 (14%)	89,212 (24%)	142,508 (38%)	91,584 (24%)	
**CV Risk Factors**						
**Any CV Risk**	261,815 (69%)	35,983 (66%)	62,068 (70%)	100,535 (71%)	63,229 (69%)	<0.001
**Hypertension**	163,429 (43%)	21,470 (40%)	38,790 (43%)	63,576 (45%)	39,593 (43%)	<0.001
**Diabetes Mellitus**	69,912 (19%)	9,644 (18%)	17,350 (19%)	27,486 (19%)	15,432 (17%)	<0.001
**Dyslipidemia**	104,316 (28%)	13,687 (25%)	24,275 (27%)	39,725 (28%)	26,629 (29%)	<0.001
**Obesity**	132,979 (35%)	18,945 (35%)	32,047 (36%)	51,591 (36%)	30,396 (33%)	<0.001
**Smoking Ever**	109,853 (29%)	14,781 (27%)	26,512 (30%)	42,951 (30%)	25,609 (28%)	<0.001
**ASCVD**						
**Any ASCVD**	35,469 (9%)	4,529 (8%)	8,416 (9%)	13,729 (10%)	8,795 (10%)	<0.001
**CAD**	22,731 (6%)	2,843 (5%)	5,285 (6%)	8,845 (6%)	5,758 (6%)	<0.001
**PAD**	7,754 (2%)	1,056 (2%)	1,891 (2%)	3,057 (2%)	1,750 (2%)	<0.001

**Stroke**	13,305 (4%)	1,724 (3%)	3,189 (4%)	5,119 (4%)	3,273 (4%)	<0.001
			**Somewhat Walkable**			
**Overall Population**	110,652	49,212 (44%)	30,776 (28%)	24,915 (23%)	5,749 (5%)	
**CV Risk Factors**						
**Any CV Risk**	65,353 (59%)	28,593 (58%)	18,535 (60%)	14,872 (60%)	3,353 (58%)	<0.001
**Hypertension**	38,373 (35%)	16,371 (33%)	10,848 (35%)	9,114 (37%)	2,040 (35%)	<0.001
**Diabetes Mellitus**	14,008 (13%)	6,178 (13%)	4,064 (13%)	3,119 (13%)	647 (11%)	<0.001
**Dyslipidemia**	30,904 (28%)	13,192 (27%)	8,823 (29%)	7,234 (29%)	1,655 (29%)	<0.001
**Obesity**	25,264 (23%)	11,600 (24%)	7,221 (23%)	5,301 (21%)	1,142 (20%)	<0.001
**Smoking Ever**	24,334 (22%)	10,692 (22%)	7,003 (23%)	5,443 (22%)	1,196 (21%)	<0.001
**ASCVD**						
**Any ASCVD**	9,689 (9%)	3,912 (8%)	2,820 (9%)	2,387 (10%)	570 (10%)	<0.001
**CAD**	6,204 (6%)	2,485 (5%)	1,831 (6%)	1,538 (6%)	350 (6%)	<0.001
**PAD**	1,720 (2%)	716 (1%)	490 (2%)	421 (2%)	93 (2%)	0.086
**Stroke**	3,921 (4%)	1,577 (3%)	1,125 (4%)	963 (4%)	256 (4%)	<0.001
			**Very Walkable / Walker's Paradise**			

**Overall Population**	26,195	19,661 (75%)	3,720 (14%)	2,263 (9%)	551 (2%)	
**CV Risk Factors**						
**Any CV Risk**	12,836 (49%)	9,479 (48%)	1,890 (51%)	1,162 (51%)	305 (55%)	<0.001
**Hypertension**	6,882 (26%)	4,895 (25%)	1,084 (29%)	702 (31%)	201 (36%)	<0.001
**Diabetes Mellitus**	1,747 (7%)	1,262 (6%)	261 (7%)	176 (8%)	48 (9%)	0.013
**Dyslipidemia**	6,547 (25%)	4,820 (25%)	996 (27%)	575 (25%)	156 (28%)	0.007
**Obesity**	4,081 (16%)	3,182 (16%)	541 (15%)	290 (13%)	68 (12%)	<0.001
**Smoking Ever**	4,129 (16%)	2,997 (15%)	626 (17%)	392 (17%)	114 (21%)	<0.001
**ASCVD**						
**Any ASCVD**	1,806 (7%)	1,216 (6%)	323 (9%)	204 (9%)	63 (11%)	<0.001
**CAD**	1,191 (5%)	813 (4%)	208 (6%)	127 (6%)	43 (8%)	<0.001
**PAD**	219 (1%)	140 (1%)	52 (1%)	23 (1%)	4 (1%)	<0.001
**Stroke**	761 (3%)	498 (3%)	142 (4%)	95 (4%)	26 (5%)	<0.001

ASCVD: atherosclerotic cardiovascular disease; CV: cardiovascular; CAD: coronary artery disease; PAD: peripheral artery disease; #Chi-square test conducted to compare the four categories of NatureScore for categorical variables.

Table S11. Univariable and multivariable logistic regression models 1-3 for all cardiovascular risk factors and diseases.

Outcomes	Exposure categories	Univariable Analysis (Model 1)	Multivariable Analysis		
			Model 2	Model 3	Model 4
**Sample size**		1,077,181	1,077,181	1,077,181	1,077,181
		OR (95% CI, p-value)	aOR (95% CI, p-value)		
**Any CV risk factor**	Nature Deficient / Nature Light	Reference	Reference		
	Nature Adequate	**1.32 (1.31- 1.34, p<0.001)**	**1.21 (1.19- 1.23, p<0.001)**	**1.17 (1.15- 1.19, p<0.001)**	0.99 (0.97- 1.00, p=0.092)
	Nature Rich	**1.42 (1.41- 1.44, p<0.001)**	**1.25 (1.23- 1.26, p<0.001)**	**1.20 (1.18- 1.21, p<0.001)**	**0.96 (0.94- 0.97, p<0.001)**
	Nature Utopia	**1.52 (1.50- 1.54, p<0.001)**	**1.30 (1.28- 1.32, p<0.001)**	**1.24 (1.22- 1.26, p<0.001)**	**0.91 (0.90- 0.93, p<0.001)**
**Hypertension**	Nature Deficient / Nature Light	Reference	Reference		

	Nature Adequate	**1.27 (1.25- 1.28, p<0.001)**	**1.14 (1.12- 1.16, p<0.001)**	**1.11 (1.10- 1.13, p<0.001)**	**0.97 (0.96- 0.99, p=0.002)**
	Nature Rich	**1.37 (1.35- 1.39, p<0.001)**	**1.17 (1.15- 1.18, p<0.001)**	**1.14 (1.12- 1.15, p<0.001)**	**0.95 (0.94- 0.97, p<0.001)**
	Nature Utopia	**1.47 (1.45- 1.49, p<0.001)**	**1.21 (1.19- 1.22, p<0.001)**	**1.17 (1.15- 1.19, p<0.001)**	**0.91 (0.90- 0.93, p<0.001)**
**Diabetes Mellitus**	Nature Deficient / Nature Light	Reference	Reference		
	Nature Adequate	**1.24 (1.22- 1.26, p<0.001)**	**1.14 (1.11- 1.16, p<0.001)**	**1.11 (1.09- 1.13, p<0.001)**	1.00 (0.98- 1.02, p=0.942)
	Nature Rich	**1.28 (1.26- 1.30, p<0.001)**	**1.15 (1.13- 1.17, p<0.001)**	**1.13 (1.11- 1.15, p<0.001)**	**0.98 (0.96- 1.00, p=0.030)**
	Nature Utopia	**1.23 (1.21- 1.25, p<0.001)**	**1.15 (1.12- 1.17, p<0.001)**	**1.13 (1.11- 1.15, p<0.001)**	**0.92 (0.90- 0.94, p<0.001)**

**Dyslipidemia**	Nature Deficient / Nature Light	Reference	Reference		
	Nature Adequate	**1.14 (1.12- 1.15, p<0.001)**	**1.08 (1.06- 1.09, p<0.001)**	**1.07 (1.06- 1.09, p<0.001)**	1.00 (0.98- 1.01, p=0.717)
	Nature Rich	**1.18 (1.17- 1.20, p<0.001)**	**1.09 (1.07- 1.10, p<0.001)**	**1.08 (1.06- 1.09, p<0.001)**	**0.98 (0.96- 0.99, p=0.009)**
	Nature Utopia	**1.25 (1.23- 1.26, p<0.001)**	**1.10 (1.09- 1.12, p<0.001)**	**1.09 (1.07- 1.10, p<0.001)**	**0.96 (0.95- 0.98, p<0.001)**
**Obesity**	Nature Deficient / Nature Light	Reference	Reference		
	Nature Adequate	**1.27 (1.25- 1.29, p<0.001)**	**1.26 (1.24- 1.28, p<0.001)**	**1.22 (1.20- 1.23, p<0.001)**	**1.02 (1.00- 1.04, p=0.016)**
	Nature Rich	**1.32 (1.31- 1.34, p<0.001)**	**1.32 (1.30- 1.33, p<0.001)**	**1.26 (1.24- 1.28, p<0.001)**	1.00 (0.99- 1.02, p=0.795)
	Nature Utopia	**1.33 (1.31- 1.34, p<0.001)**	**1.39 (1.37- 1.41, p<0.001)**	**1.32 (1.30- 1.34, p<0.001)**	**0.96 (0.94- 0.97, p<0.001)**

**Smoking**	Nature Deficient / Nature Light	Reference	Reference		
	Nature Adequate	**1.21 (1.19- 1.23, p<0.001)**	**1.12 (1.10- 1.14, p<0.001)**	**1.09 (1.07- 1.11, p<0.001)**	1.00 (0.98- 1.01, p=0.710)
	Nature Rich	**1.27 (1.25- 1.29, p<0.001)**	**1.12 (1.11- 1.14, p<0.001)**	**1.10 (1.08- 1.11, p<0.001)**	**0.98 (0.96- 0.99, p=0.003)**
	Nature Utopia	**1.33 (1.31- 1.35, p<0.001)**	**1.15 (1.13- 1.17, p<0.001)**	**1.12 (1.10- 1.14, p<0.001)**	**0.94 (0.92- 0.95, p<0.001)**
**Any ASCVD**	Nature Deficient / Nature Light	Reference	Reference		
	Nature Adequate	**1.19 (1.16- 1.21, p<0.001)**	**1.08 (1.05- 1.11, p<0.001)**	**1.07 (1.04- 1.10, p<0.001)**	0.99 (0.96- 1.02, p=0.491)
	Nature Rich	**1.25 (1.22- 1.28, p<0.001)**	**1.06 (1.04- 1.09, p<0.001)**	**1.06 (1.03- 1.08, p<0.001)**	**0.95 (0.93- 0.98, p<0.001)**

	Nature Utopia	**1.38 (1.35- 1.41, p<0.001)**	**1.11 (1.09- 1.14, p<0.001)**	**1.11 (1.08- 1.14, p<0.001)**	**0.96 (0.93- 0.98, p=0.002)**
**CAD**	Nature Deficient / Nature Light	Reference	Reference		
	Nature Adequate	**1.19 (1.16- 1.22, p<0.001)**	**1.09 (1.05- 1.12, p<0.001)**	**1.08 (1.04- 1.11, p<0.001)**	0.98 (0.95- 1.01, p=0.154)
	Nature Rich	**1.27 (1.24- 1.31, p<0.001)**	**1.09 (1.06- 1.12, p<0.001)**	**1.08 (1.05- 1.11, p<0.001)**	**0.95 (0.92- 0.98, p=0.002)**
	Nature Utopia	**1.43 (1.39- 1.47, p<0.001)**	**1.15 (1.11- 1.18, p<0.001)**	**1.14 (1.11- 1.17, p<0.001)**	**0.95 (0.92- 0.98, p=0.003)**
**PAD**	Nature Deficient / Nature Light	Reference	Reference		
	Nature Adequate	**1.22 (1.16- 1.29, p<0.001)**	**1.11 (1.05- 1.17, p<0.001)**	**1.09 (1.03- 1.15, p=0.001)**	0.97 (0.92- 1.03, p=0.293)

	Nature Rich	**1.32 (1.26- 1.38, p<0.001)**	**1.13 (1.08- 1.18, p<0.001)**	**1.11 (1.06- 1.16, p<0.001)**	**0.95 (0.90- 1.00, p=0.046)**
	Nature Utopia	**1.36 (1.30- 1.43, p<0.001)**	**1.15 (1.10- 1.21, p<0.001)**	**1.14 (1.08- 1.19, p<0.001)**	**0.93 (0.88- 0.98, p=0.012)**
**Stroke**	Nature Deficient / Nature Light	Reference		Reference	
	Nature Adequate	**1.14 (1.10- 1.19, p<0.001)**	1.04 (1.00- 1.07, p=0.069)	1.03 (1.00- 1.07, p=0.087)	1.01 (0.97- 1.05, p=0.669)
	Nature Rich	**1.18 (1.14- 1.22, p<0.001)**	1.01 (0.98- 1.04, p=0.596)	1.01 (0.98- 1.05, p=0.559)	0.97 (0.94- 1.01, p=0.165)
	Nature Utopia	**1.25 (1.21-1.30, p<0.001)**	1.03 (1.00- 1.07, p=0.056)	1.04 (1.00- 1.07, p=0.041)	0.98 (0.94- 1.03, p=0.426)

aOR: Adjusted odds ratio; ASCVD: Atherosclerotic cardiovascular disease; CAD: Coronary artery disease; CI: Confidence intervals; CV: Cardiovascular; PAD: Peripheral artery disease

Model 2: Adjusted for age, sex, and race/ethnicity.

Model 3: Adjusted for Model 2 variables plus socioeconomic status (Area Deprivation Index -ADI-).

Model 4: Adjusted for Model 3 variables plus WalkScore.

Table S12. Prevalence of cardiovascular diseases and cardiovascular risk factors stratified by sex (n=1,077,181).

Characteristics	Total Population	NatureScore Groups				
		Nature Deficient / Nature Light (0-39)	Nature Adequate (40-59)	Nature Rich (60-79)	Nature Utopia (80-100)	p-value#
**Male**						
**Overall Population**	442,517	66,669 (15%)	90,788 (21%)	142,696 (32%)	142,364 (32%)	
**CV Risk Factors**						
**Any CV Risk**	316,498 (72%)	42,466 (64%)	64,216 (71%)	103,427 (72%)	106,389 (75%)	<0.001
**Hypertension**	205,868 (47%)	25,746 (39%)	40,801 (45%)	67,662 (47%)	71,659 (50%)	<0.001
**Diabetes Mellitus**	85,618 (19%)	10,465 (16%)	17,655 (19%)	28,935 (20%)	28,563 (20%)	<0.001
**Dyslipidemia**	142,365 (32%)	19,391 (29%)	28,806 (32%)	46,298 (32%)	47,870 (34%)	<0.001
**Obesity**	148,654 (34%)	18,039 (27%)	29,902 (33%)	49,226 (34%)	51,487 (36%)	<0.001
**Smoking Ever**	143,273 (32%)	17,810 (27%)	28,811 (32%)	47,407 (33%)	49,245 (35%)	<0.001
**ASCVD**						
**Any ASCVD**	55,725 (13%)	6,616 (10%)	10,885 (12%)	18,177 (13%)	20,047 (14%)	<0.001

**CAD**	40,906 (9%)	4,767 (7%)	7,866 (9%)	13,287 (9%)	14,986 (11%)	<0.001
**PAD**	10,956 (2%)	1,217 (2%)	2,211 (2%)	3,672 (3%)	3,856 (3%)	<0.001
**Stroke**	17,291 (4%)	2,186 (3%)	3,416 (4%)	5,693 (4%)	5,996 (4%)	<0.001
			**Female**			
**Overall Population**	634,664	94,149 (15%)	133,375 (21%)	209,246 (33%)	197,894 (31%)	
**CV Risk Factors**						
**Any CV Risk**	416,716 (66%)	55,874 (59%)	87,285 (65%)	139,982 (67%)	133,575 (67%)	<0.001
**Hypertension**	243,484 (38%)	30,887 (33%)	50,592 (38%)	82,552 (39%)	79,453 (40%)	<0.001
**Diabetes Mellitus**	93,631 (15%)	12,302 (13%)	20,351 (15%)	32,270 (15%)	28,708 (15%)	<0.001
**Dyslipidemia**	173,739 (27%)	22,896 (24%)	35,886 (27%)	58,031 (28%)	56,926 (29%)	<0.001
**Obesity**	213,514 (34%)	28,057 (30%)	45,761 (34%)	72,924 (35%)	66,772 (34%)	<0.001
**Smoking Ever**	150,755 (24%)	19,705 (21%)	31,558 (24%)	50,666 (24%)	48,826 (25%)	<0.001
**ASCVD**						
**Any ASCVD**	46,513 (7%)	5,928 (6%)	9,571 (7%)	15,461 (7%)	15,553 (8%)	<0.001
**CAD**	26,486 (4%)	3,299 (4%)	5,371 (4%)	8,893 (4%)	8,923 (5%)	<0.001
**PAD**	9,694 (2%)	1,230 (1%)	1,952 (1%)	3,359 (2%)	3,153 (2%)	<0.001

**Stroke**	20,575 (3%)	2,673 (3%)	4,293 (3%)	6,808 (3%)	6,801 (3%)	<0.001

ASCVD: atherosclerotic cardiovascular disease; CV: cardiovascular; CAD: coronary artery disease; PAD: peripheral artery disease; #Chi-square test conducted to compare the four categories of NatureScore for categorical variables.

Table S13. Prevalence of cardiovascular diseases and cardiovascular risk factors stratified by age group (n=1,077,181).

Characteristics	Total Population	NatureScore Groups: Nature Deficient / Nature Light (0-39)	Nature Adequate (40-59)	Nature Rich (60-79)	Nature Utopia (80-100)	p-value#
**Age Group 18-39**						
**Overall Population**	314,776	62,315 (20%)	69,588 (22%)	99,286 (32%)	83,587 (27%)	
**CV Risk Factors**						
**Any CV Risk**	137,769 (44%)	24,489 (39%)	31,145 (45%)	44,781 (45%)	37,354 (45%)	<0.001
**Hypertension**	30,391 (10%)	5,309 (9%)	6,830 (10%)	10,079 (10%)	8,173 (10%)	<0.001
**Diabetes Mellitus**	10,684 (3%)	1,792 (3%)	2,465 (4%)	3,613 (4%)	2,814 (3%)	<0.001
**Dyslipidemia**	47,437 (15%)	9,483 (15%)	10,891 (16%)	14,858 (15%)	12,205 (15%)	<0.001
**Obesity**	92,552 (29%)	15,057 (24%)	21,080 (30%)	30,795 (31%)	25,620 (31%)	<0.001
**Smoking Ever**	30,073 (10%)	4,973 (8%)	6,619 (10%)	9,895 (10%)	8,586 (10%)	<0.001
**ASCVD**						
**Any ASCVD**	1,919 (1%)	324 (1%)	429 (1%)	623 (1%)	543 (1%)	0.011

**CAD**	742 (0%)	108 (0%)	201 (0%)	214 (0%)	219 (0%)	<0.001
**PAD**	265 (0%)	55 (0%)	42 (0%)	93 (0%)	75 (0%)	0.10
**Stroke**	982 (0%)	171 (0%)	201 (0%)	341 (0%)	269 (0%)	0.059
**Age Group 40-64**						
**Overall Population**	450,291	59,916 (13%)	93,717 (21%)	149,426 (33%)	147,232 (33%)	
**CV Risk Factors**						
**Any CV Risk**	322,089 (72%)	40,791 (68%)	67,085 (72%)	107,684 (72%)	106,529 (72%)	<0.001
**Hypertension**	186,809 (41%)	23,410 (39%)	39,136 (42%)	62,678 (42%)	61,585 (42%)	<0.001
**Diabetes Mellitus**	73,710 (16%)	9,478 (16%)	16,266 (17%)	25,090 (17%)	22,876 (16%)	<0.001
**Dyslipidemia**	144,794 (32%)	18,210 (30%)	30,094 (32%)	48,069 (32%)	48,421 (33%)	<0.001
**Obesity**	176,655 (39%)	20,964 (35%)	36,808 (39%)	60,071 (40%)	58,812 (40%)	<0.001
**Smoking Ever**	119,806 (27%)	15,028 (25%)	25,285 (27%)	39,867 (27%)	39,626 (27%)	<0.001
**ASCVD**						
**Any ASCVD**	29,291 (7%)	3,866 (6%)	6,201 (7%)	9,397 (6%)	9,827 (7%)	<0.001
**CAD**	18,878 (4%)	2,468 (4%)	3,914 (4%)	6,040 (4%)	6,456 (4%)	<0.001
**PAD**	4,742 (1%)	641 (1%)	1,047 (1%)	1,542 (1%)	1,512 (1%)	0.14

**Stroke**	9,876 (2%)	1,352 (2%)	2,158 (2%)	3,173 (2%)	3,193 (2%)	0.017
			**Age Group 65-79**			
**Overall Population**	243,760	29,615 (12%)	47,471 (19%)	80,473 (33%)	86,201 (35%)	
**CV Risk Factors**						
**Any CV Risk**	211,323 (87%)	25,029 (85%)	41,147 (87%)	70,223 (87%)	74,924 (87%)	<0.001
**Hypertension**	174,931 (72%)	20,565 (69%)	34,257 (72%)	58,302 (72%)	61,807 (72%)	<0.001
**Diabetes Mellitus**	74,662 (31%)	8,995 (30%)	15,297 (32%)	25,562 (32%)	24,808 (29%)	<0.001
**Dyslipidemia**	98,821 (41%)	11,383 (38%)	18,888 (40%)	33,082 (41%)	35,468 (41%)	<0.001
**Obesity**	80,928 (33%)	8,650 (29%)	15,487 (33%)	27,178 (34%)	29,613 (34%)	<0.001
**Smoking Ever**	111,921 (46%)	13,365 (45%)	22,112 (47%)	37,526 (47%)	38,918 (45%)	<0.001
**ASCVD**						
**Any ASCVD**	50,851 (21%)	5,843 (20%)	9,837 (21%)	16,983 (21%)	18,188 (21%)	<0.001
**CAD**	34,705 (14%)	3,896 (13%)	6,623 (14%)	11,638 (14%)	12,548 (15%)	<0.001
**PAD**	10,768 (4%)	1,188 (4%)	2,088 (4%)	3,731 (5%)	3,761 (4%)	<0.001
**Stroke**	18,604 (8%)	2,213 (7%)	3,647 (8%)	6,267 (8%)	6,477 (8%)	0.13
			**Age Group 80+**			

**Overall Population**	68,354	8,972 (13%)	13,387 (20%)	22,757 (33%)	23,238 (34%)	
**CV Risk Factors**						
**Any CV Risk**	62,033 (91%)	8,031 (90%)	12,124 (91%)	20,721 (91%)	21,157 (91%)	<0.001
**Hypertension**	57,221 (84%)	7,349 (82%)	11,170 (83%)	19,155 (84%)	19,547 (84%)	<0.001
**Diabetes Mellitus**	20,193 (30%)	2,502 (28%)	3,978 (30%)	6,940 (30%)	6,773 (29%)	<0.001
**Dyslipidemia**	25,052 (37%)	3,211 (36%)	4,819 (36%)	8,320 (37%)	8,702 (37%)	0.008
**Obesity**	12,033 (18%)	1,425 (16%)	2,288 (17%)	4,106 (18%)	4,214 (18%)	<0.001
**Smoking Ever**	32,228 (47%)	4,149 (46%)	6,353 (47%)	10,785 (47%)	10,941 (47%)	0.26
**ASCVD**						
**Any ASCVD**	20,177 (30%)	2,511 (28%)	3,989 (30%)	6,635 (29%)	7,042 (30%)	<0.001
**CAD**	13,067 (19%)	1,594 (18%)	2,499 (19%)	4,288 (19%)	4,686 (20%)	<0.001
**PAD**	4,875 (7%)	563 (6%)	986 (7%)	1,665 (7%)	1,661 (7%)	0.007
**Stroke**	8,404 (12%)	1,123 (13%)	1,703 (13%)	2,720 (12%)	2,858 (12%)	0.16

ASCVD: atherosclerotic cardiovascular disease; CV: cardiovascular; CAD: coronary artery disease; PAD: peripheral artery disease; [#]Chi-square test conducted to compare the four categories of NatureScore for categorical variables.

Table S14. Prevalence of cardiovascular diseases and cardiovascular risk factors stratified by race/ethnicity (n=1,077,181).

Characteristics	Total Population	NatureScore Groups				
		Nature Deficient / Nature Light (0-39)	Nature Adequate (40-59)	Nature Rich (60-79)	Nature Utopia (80-100)	p-value[#]
**Hispanic**						
**Overall Population**	172,066	28,645 (17%)	40,684 (24%)	59,142 (34%)	43,595 (25%)	
**CV Risk Factors**						
**Any CV Risk**	116,396 (68%)	18,308 (64%)	27,628 (68%)	40,371 (68%)	30,089 (69%)	<0.001
**Hypertension**	60,219 (35%)	9,449 (33%)	14,287 (35%)	20,860 (35%)	15,623 (36%)	<0.001
**Diabetes Mellitus**	33,645 (20%)	5,352 (19%)	8,149 (20%)	11,618 (20%)	8,526 (20%)	<0.001
**Dyslipidemia**	48,660 (28%)	7,578 (26%)	11,364 (28%)	16,854 (28%)	12,864 (30%)	<0.001
**Obesity**	68,366 (40%)	10,100 (35%)	16,343 (40%)	24,066 (41%)	17,857 (41%)	<0.001
**Smoking Ever**	45,794 (27%)	7,295 (25%)	10,948 (27%)	15,891 (27%)	11,660 (27%)	<0.001
**ASCVD**						
**Any ASCVD**	12,034 (7%)	1,892 (7%)	2,859 (7%)	4,134 (7%)	3,149 (7%)	0.016

**CAD**	7,558 (4%)	1,158 (4%)	1,763 (4%)	2,626 (4%)	2,011 (5%)	0.003
**PAD**	2,791 (2%)	491 (2%)	703 (2%)	907 (2%)	690 (2%)	0.053
**Stroke**	4,387 (3%)	706 (2%)	998 (2%)	1,504 (3%)	1,179 (3%)	0.087
			**Non-Hispanic White**			
**Overall Population**	582,554	74,639 (13%)	103,704 (18%)	181,101 (31%)	223,110 (38%)	
**CV Risk Factors**						
**Any CV Risk**	404,415 (69%)	45,978 (62%)	70,666 (68%)	126,851 (70%)	160,920 (72%)	<0.001
**Hypertension**	256,728 (44%)	27,448 (37%)	44,026 (42%)	80,435 (44%)	104,819 (47%)	<0.001
**Diabetes Mellitus**	86,154 (15%)	8,615 (12%)	14,878 (14%)	27,513 (15%)	35,148 (16%)	<0.001
**Dyslipidemia**	182,239 (31%)	21,134 (28%)	31,813 (31%)	57,364 (32%)	71,928 (32%)	<0.001
**Obesity**	186,426 (32%)	18,814 (25%)	32,178 (31%)	59,169 (33%)	76,265 (34%)	<0.001
**Smoking Ever**	166,598 (29%)	17,850 (24%)	29,008 (28%)	52,063 (29%)	67,677 (30%)	<0.001
**ASCVD**						
**Any ASCVD**	63,928 (11%)	6,897 (9%)	11,076 (11%)	19,734 (11%)	26,221 (12%)	<0.001
**CAD**	43,419 (7%)	4,594 (6%)	7,417 (7%)	13,445 (7%)	17,963 (8%)	<0.001
**PAD**	12,006 (2%)	1,137 (2%)	2,026 (2%)	3,831 (2%)	5,012 (2%)	<0.001

**Stroke**	23,166 (4%)	2,698 (4%)	4,143 (4%)	7,163 (4%)	9,162 (4%)	<0.001
			**Non-Hispanic Black**			
**Overall Population**	155,766	26,571 (17%)	40,419 (26%)	57,845 (37%)	30,931 (20%)	
**CV Risk Factors**						
**Any CV Risk**	120,651 (77%)	19,366 (73%)	31,251 (77%)	45,664 (79%)	24,370 (79%)	<0.001
**Hypertension**	83,352 (54%)	12,694 (48%)	21,309 (53%)	32,238 (56%)	17,111 (55%)	<0.001
**Diabetes Mellitus**	36,292 (23%)	5,526 (21%)	9,218 (23%)	14,114 (24%)	7,434 (24%)	<0.001
**Dyslipidemia**	40,522 (26%)	6,220 (23%)	10,601 (26%)	15,326 (26%)	8,375 (27%)	<0.001
**Obesity**	73,444 (47%)	11,989 (45%)	19,336 (48%)	27,483 (48%)	14,636 (47%)	<0.001
**Smoking Ever**	51,042 (33%)	7,888 (30%)	12,971 (32%)	19,733 (34%)	10,450 (34%)	<0.001
**ASCVD**						
**Any ASCVD**	16,239 (10%)	2,335 (9%)	4,088 (10%)	6,391 (11%)	3,425 (11%)	<0.001
**CAD**	9,433 (6%)	1,367 (5%)	2,344 (6%)	3,779 (7%)	1,943 (6%)	<0.001
**PAD**	4,211 (3%)	598 (2%)	1,037 (3%)	1,718 (3%)	858 (3%)	<0.001
**Stroke**	6,541 (4%)	914 (3%)	1,662 (4%)	2,570 (4%)	1,395 (5%)	<0.001
			**Non-Hispanic Asian**			

**Overall Population**	80,454	14,857 (18%)	20,795 (26%)	26,370 (33%)	18,432 (23%)	
**CV Risk Factors**						
**Any CV Risk**	47,038 (58%)	7,734 (52%)	12,382 (60%)	15,822 (60%)	11,100 (60%)	<0.001
**Hypertension**	26,590 (33%)	3,936 (26%)	7,026 (34%)	9,211 (35%)	6,417 (35%)	<0.001
**Diabetes Mellitus**	14,283 (18%)	2,020 (14%)	3,793 (18%)	5,001 (19%)	3,469 (19%)	<0.001
**Dyslipidemia**	27,089 (34%)	4,491 (30%)	7,117 (34%)	9,065 (34%)	6,416 (35%)	<0.001
**Obesity**	11,151 (14%)	1,876 (13%)	2,995 (14%)	3,706 (14%)	2,574 (14%)	<0.001
**Smoking Ever**	17,604 (22%)	2,628 (18%)	4,652 (22%)	6,107 (23%)	4,217 (23%)	<0.001
**ASCVD**						
**Any ASCVD**	6,452 (8%)	917 (6%)	1,683 (8%)	2,241 (8%)	1,611 (9%)	<0.001
**CAD**	4,596 (6%)	626 (4%)	1,216 (6%)	1,581 (6%)	1,173 (6%)	<0.001
**PAD**	1,003 (1%)	132 (1%)	255 (1%)	361 (1%)	255 (1%)	<0.001
**Stroke**	2,556 (3%)	366 (2%)	660 (3%)	884 (3%)	646 (4%)	<0.001
**Non-Hispanic Others**						
**Overall Population**	86,341	16,106 (19%)	18,561 (21%)	27,484 (32%)	24,190 (28%)	

**CV Risk Factors**						
**Any CV Risk**	44,714 (52%)	6,954 (43%)	9,574 (52%)	14,701 (53%)	13,485 (56%)	<0.001
**Hypertension**	22,463 (26%)	3,106 (19%)	4,745 (26%)	7,470 (27%)	7,142 (30%)	<0.001
**Diabetes Mellitus**	8,875 (10%)	1,254 (8%)	1,968 (11%)	2,959 (11%)	2,694 (11%)	<0.001
**Dyslipidemia**	17,594 (20%)	2,864 (18%)	3,797 (20%)	5,720 (21%)	5,213 (22%)	<0.001
**Obesity**	22,781 (26%)	3,317 (21%)	4,811 (26%)	7,726 (28%)	6,927 (29%)	<0.001
**Smoking Ever**	12,990 (15%)	1,854 (12%)	2,790 (15%)	4,279 (16%)	4,067 (17%)	<0.001
**ASCVD**						
**Any ASCVD**	3,585 (4%)	503 (3%)	750 (4%)	1,138 (4%)	1,194 (5%)	<0.001
**CAD**	2,386 (3%)	321 (2%)	497 (3%)	749 (3%)	819 (3%)	<0.001
**PAD**	639 (1%)	89 (1%)	142 (1%)	214 (1%)	194 (1%)	0.021
**Stroke**	1,216 (1%)	175 (1%)	246 (1%)	380 (1%)	415 (2%)	<0.001

ASCVD: atherosclerotic cardiovascular disease; CV: cardiovascular; CAD: coronary artery disease; PAD: peripheral artery disease; [#]Chi-square test conducted to compare the four categories of NatureScore for categorical variables.

Table S15. Prevalence of cardiovascular diseases and cardiovascular risk factors stratified by area deprivation index (n=1,077,181).

Characteristics	Total Population	NatureScore Groups				
		Nature Deficient / Nature Light (0-39)	Nature Adequate (40-59)	Nature Rich (60-79)	Nature Utopia (80-100)	p-value#
**1st Quintile (Least Deprived)**						
**Overall Population**	253,168	52,114 (21%)	53,986 (21%)	72,663 (29%)	74,405 (29%)	
**CV Risk Factors**						
**Any CV Risk**	149,074 (59%)	27,829 (53%)	31,263 (58%)	43,530 (60%)	46,452 (62%)	<0.001
**Hypertension**	85,240 (34%)	14,773 (28%)	17,419 (32%)	25,213 (35%)	27,835 (37%)	<0.001
**Diabetes Mellitus**	26,352 (10%)	4,248 (8%)	5,477 (10%)	7,969 (11%)	8,658 (12%)	<0.001
**Dyslipidemia**	74,929 (30%)	14,012 (27%)	15,937 (30%)	22,044 (30%)	22,936 (31%)	<0.001
**Obesity**	56,709 (22%)	10,005 (19%)	11,596 (21%)	16,641 (23%)	18,467 (25%)	<0.001
**Smoking Ever**	48,320 (19%)	8,726 (17%)	10,058 (19%)	14,019 (19%)	15,517 (21%)	<0.001
**ASCVD**						
**Any ASCVD**	20,319 (8%)	3,470 (7%)	4,291 (8%)	5,905 (8%)	6,653 (9%)	<0.001

**CAD**	13,733 (5%)	2,274 (4%)	2,906 (5%)	4,037 (6%)	4,516 (6%)	<0.001
**PAD**	2,911 (1%)	450 (1%)	591 (1%)	838 (1%)	1,032 (1%)	<0.001
**Stroke**	7,791 (3%)	1,421 (3%)	1,688 (3%)	2,247 (3%)	2,435 (3%)	<0.001
			**2nd Quintile**			
**Overall Population**	319,725	33,329 (10%)	60,031 (19%)	113,276 (35%)	113,089 (35%)	
**CV Risk Factors**						
**Any CV Risk**	215,063 (67%)	19,914 (60%)	40,404 (67%)	76,640 (68%)	78,105 (69%)	<0.001
**Hypertension**	129,320 (40%)	11,180 (34%)	23,606 (39%)	46,078 (41%)	48,456 (43%)	<0.001
**Diabetes Mellitus**	47,493 (15%)	4,214 (13%)	8,916 (15%)	17,043 (15%)	17,320 (15%)	<0.001
**Dyslipidemia**	97,820 (31%)	8,929 (27%)	18,204 (30%)	35,101 (31%)	35,586 (31%)	<0.001
**Obesity**	104,235 (33%)	9,117 (27%)	20,301 (34%)	37,340 (33%)	37,477 (33%)	<0.001
**Smoking Ever**	80,268 (25%)	7,218 (22%)	14,768 (25%)	28,354 (25%)	29,928 (26%)	<0.001
**ASCVD**						
**Any ASCVD**	29,073 (9%)	2,500 (8%)	4,950 (8%)	10,272 (9%)	11,351 (10%)	<0.001
**CAD**	19,609 (6%)	1,653 (5%)	3,234 (5%)	6,975 (6%)	7,747 (7%)	<0.001
**PAD**	5,427 (2%)	433 (1%)	935 (2%)	1,982 (2%)	2,077 (2%)	<0.001

**Stroke**	10,701 (3%)	974 (3%)	1,866 (3%)	3,796 (3%)	4,065 (4%)	<0.001
			**3rd Quintile**			
**Overall Population**	259,515	31,931 (12%)	53,090 (20%)	85,934 (33%)	88,560 (34%)	
**CV Risk Factors**						
**Any CV Risk**	185,133 (71%)	20,516 (64%)	37,140 (70%)	61,909 (72%)	65,568 (74%)	<0.001
**Hypertension**	113,558 (44%)	11,907 (37%)	22,544 (42%)	37,880 (44%)	41,227 (47%)	<0.001
**Diabetes Mellitus**	47,245 (18%)	4,861 (15%)	9,744 (18%)	16,244 (19%)	16,396 (19%)	<0.001
**Dyslipidemia**	75,315 (29%)	8,260 (26%)	14,861 (28%)	24,908 (29%)	27,286 (31%)	<0.001
**Obesity**	99,616 (38%)	10,758 (34%)	20,135 (38%)	33,786 (39%)	34,937 (39%)	<0.001
**Smoking Ever**	78,028 (30%)	7,961 (25%)	15,371 (29%)	26,200 (30%)	28,496 (32%)	<0.001
**ASCVD**						
**Any ASCVD**	24,633 (9%)	2,411 (8%)	4,704 (9%)	8,049 (9%)	9,469 (11%)	<0.001
**CAD**	16,212 (6%)	1,562 (5%)	3,034 (6%)	5,265 (6%)	6,351 (7%)	<0.001
**PAD**	5,275 (2%)	512 (2%)	981 (2%)	1,776 (2%)	2,006 (2%)	<0.001
**Stroke**	8,931 (3%)	888 (3%)	1,768 (3%)	2,934 (3%)	3,341 (4%)	<0.001
			**4th Quintile**			

**Overall Population**	165,410	28,298 (17%)	37,673 (23%)	55,172 (33%)	44,267 (27%)	
**CV Risk Factors**						
**Any CV Risk**	122,902 (74%)	19,234 (68%)	27,769 (74%)	41,840 (76%)	34,059 (77%)	<0.001
**Hypertension**	79,319 (48%)	11,690 (41%)	17,714 (47%)	27,276 (49%)	22,639 (51%)	<0.001
**Diabetes Mellitus**	37,306 (23%)	5,701 (20%)	8,688 (23%)	13,025 (24%)	9,892 (22%)	<0.001
**Dyslipidemia**	46,068 (28%)	7,182 (25%)	10,367 (28%)	15,305 (28%)	13,214 (30%)	<0.001
**Obesity**	67,870 (41%)	10,363 (37%)	15,382 (41%)	23,498 (43%)	18,627 (42%)	<0.001
**Smoking Ever**	56,834 (34%)	8,359 (30%)	12,777 (34%)	19,631 (36%)	16,067 (36%)	<0.001
**ASCVD**						
**Any ASCVD**	18,106 (11%)	2,476 (9%)	4,054 (11%)	6,089 (11%)	5,487 (12%)	<0.001
**CAD**	11,576 (7%)	1,557 (6%)	2,556 (7%)	3,857 (7%)	3,606 (8%)	<0.001
**PAD**	4,336 (3%)	604 (2%)	985 (3%)	1,519 (3%)	1,228 (3%)	<0.001
**Stroke**	6,750 (4%)	945 (3%)	1,500 (4%)	2,286 (4%)	2,019 (5%)	<0.001
**5th Quintile (Most Deprived)**						
**Overall Population**	76,240	13,996 (18%)	18,975 (25%)	24,018 (32%)	19,251 (25%)	

**CV Risk Factors**						
**Any CV Risk**	59,344 (78%)	10,234 (73%)	14,648 (77%)	19,010 (79%)	15,452 (80%)	<0.001
**Hypertension**	40,864 (54%)	6,747 (48%)	9,935 (52%)	13,453 (56%)	10,729 (56%)	<0.001
**Diabetes Mellitus**	20,484 (27%)	3,647 (26%)	5,111 (27%)	6,803 (28%)	4,923 (26%)	<0.001
**Dyslipidemia**	21,260 (28%)	3,621 (26%)	5,206 (27%)	6,793 (28%)	5,640 (29%)	<0.001
**Obesity**	33,002 (43%)	5,617 (40%)	8,126 (43%)	10,661 (44%)	8,598 (45%)	<0.001
**Smoking Ever**	29,939 (39%)	5,047 (36%)	7,277 (38%)	9,679 (40%)	7,936 (41%)	<0.001
**ASCVD**						
**Any ASCVD**	9,867 (13%)	1,615 (12%)	2,410 (13%)	3,256 (14%)	2,586 (13%)	<0.001
**CAD**	6,120 (8%)	976 (7%)	1,484 (8%)	2,008 (8%)	1,652 (9%)	<0.001
**PAD**	2,659 (3%)	433 (3%)	662 (3%)	909 (4%)	655 (3%)	0.004
**Stroke**	3,598 (5%)	605 (4%)	867 (5%)	1,206 (5%)	920 (5%)	0.012

ASCVD: atherosclerotic cardiovascular disease; CV: cardiovascular; CAD: coronary artery disease; PAD: peripheral artery disease; [#]Chi-square test conducted to compare the four categories of NatureScore for categorical variables.

Table S16. Multivariable logistic regression for cardiovascular risk factors across various NatureScore groups, stratified by neighborhood walkability using subgroup analysis of ASCVD population (n=1,077,181).

		Multivariable Analysis after Stratification using Model 4			
**Outcome**	**Exposure Categories**	**Car-Dependent (All Errands) (0-19)**	**Car-Dependent (Most Errands) (20-39)**	**Somewhat Walkable (40-59)**	**Very Walkable / Walker's Paradise (60-100)**
		**aOR (95% CI, p-value)**			
**ASCVD Population (n=102,238)**					
**Any CV risk factor**	Nature Deficient / Nature Light	Reference			
	Nature Adequate	1.15 (0.85-1.54, p=0.360)	0.91 (0.72-1.14, p=0.403)	0.94 (0.74-1.20, p=0.630)	0.93 (0.56-1.54, p=0.777)
	Nature Rich	1.12 (0.85-1.49, p=0.415)	0.83 (0.67-1.03, p=0.084)	1.03 (0.79-1.33, p=0.834)	1.18 (0.59-2.35, p=0.640)
	Nature Utopia	1.07 (0.81-1.41, p=0.619)	**0.78 (0.62-0.98, p=0.033)**	**0.67 (0.46-0.99, p=0.043)**	1.28 (0.39-4.24, p=0.683)
**Hypertension**	Nature Deficient / Nature Light	Reference			

	Nature Adequate	1.02 (0.86-1.22, p=0.815)	0.99 (0.86-1.13, p=0.848)	0.98 (0.85-1.14, p=0.825)	1.06 (0.76-1.49, p=0.713)
	Nature Rich	1.08 (0.91-1.27, p=0.391)	0.90 (0.80-1.02, p=0.107)	0.92 (0.78-1.07, p=0.264)	0.94 (0.63-1.41, p=0.764)
	Nature Utopia	1.09 (0.93-1.29, p=0.294)	0.88 (0.78-1.01, p=0.064)	**0.74 (0.58-0.96, p=0.020)**	0.71 (0.38-1.33, p=0.268)
**Diabetes Mellitus**	Nature Deficient / Nature Light	Reference			
	Nature Adequate	1.00 (0.90-0.90, p=0.957)	0.94 (0.87-1.01, p=0.089)	0.98 (0.89-1.09, p=0.745)	1.26 (0.96-1.67, p=0.101)
	Nature Rich	0.99 (0.89-1.10, p=0.869)	**0.92 (0.86-0.98, p=0.014)**	**0.88 (0.79-0.98, p=0.021)**	1.27 (0.91-1.77, p=0.164)
	Nature Utopia	0.94 (0.85-1.05, p=0.275)	**0.91 (0.85-0.98, p=0.013)**	**0.75 (0.62-0.91, p=0.004)**	1.33 (0.75-2.36, p=0.335)
**Dyslipidemia**	Nature Deficient / Nature Light	Reference			

	Nature Adequate	1.00 (0.89-1.11, p=0.932)	1.03 (0.96-1.11, p=0.450)	1.04 (0.94-1.15, p=0.419)	1.14 (0.89-1.46, p=0.314)
	Nature Rich	1.03 (0.93-1.14, p=0.564)	**1.08 (1.01-1.16, p=0.023)**	1.07 (0.96-1.18, p=0.215)	1.01 (0.75-1.36, p=0.953)
	Nature Utopia	0.97 (0.87-1.07, p=0.501)	1.06 (0.99-1.14, p=0.093)	0.97 (0.81-1.15, p=0.709)	0.95 (0.57-1.59, p=0.852)
**Obesity**	Nature Deficient / Nature Light	Reference			
	Nature Adequate	0.98 (0.87-1.10, p=0.682)	1.00 (0.92-1.08, p=0.961)	1.06 (0.95-1.18, p=0.319)	**0.71 (0.52-0.98, p=0.040)**
	Nature Rich	1.01 (0.90-1.12, p=0.898)	1.00 (0.93-1.07, p=0.930)	1.04 (0.92-1.17, p=0.555)	0.95 (0.66-1.37, p=0.777)
	Nature Utopia	0.97 (0.87-1.08, p=0.545)	1.00 (0.92-1.08, p=0.950)	1.05 (0.86-1.29, p=0.629)	0.87 (0.45-1.67, p=0.673)
**Smoking**	Nature Deficient / Nature Light	Reference			

	Nature Adequate	0.99 (0.88-1.11, p=0.810)	0.93 (0.86-1.01, p=0.068)	0.94 (0.85-1.04, p=0.197)	1.05 (0.82-1.35, p=0.700)
	Nature Rich	0.97 (0.87-1.08, p=0.590)	**0.93 (0.86-1.00, p=0.049)**	**0.85 (0.76-0.94, p=0.002)**	1.00 (0.74-1.35, p=0.976)
	Nature Utopia	0.96 (0.86-1.06, p=0.411)	0.93 (0.86-1.00, p=0.053)	**0.78 (0.65-0.93, p=0.006)**	0.97 (0.58-1.62, p=0.913)
		**Non-ASCVD Population (n=974,943)**			
**Any CV risk factor**	Nature Deficient / Nature Light	Reference			
	Nature Adequate	0.99 (0.95-1.03, p=0.614)	0.99 (0.95-1.03, p=0.614)	0.99 (0.95-1.03, p=0.614)	0.99 (0.95-1.03, p=0.614)
	Nature Rich	**0.96 (0.92-0.99, p=0.017)**	**0.96 (0.92-0.99, p=0.017)**	**0.96 (0.92-0.99, p=0.017)**	**0.96 (0.92-0.99, p=0.017)**
	Nature Utopia	**0.92 (0.89-0.95, p<0.001)**	**0.92 (0.89-0.95, p<0.001)**	**0.92 (0.89-0.95, p<0.001)**	**0.92 (0.89-0.95, p<0.001)**
**Hypertension**	Nature Deficient / Nature Light	Reference			

	Nature Adequate	0.97 (0.93-1.01, p=0.133)	**1.05 (1.02-1.08, p=0.001)**	**0.93 (0.90-0.97, p=0.001)**	**0.89 (0.81-0.99, p=0.029)**
	Nature Rich	**0.95 (0.92-0.99, p=0.010)**	**1.05 (1.02-1.08, p<0.001)**	**0.93 (0.89-0.97, p<0.001)**	**0.82 (0.73-0.93, p=0.002)**
	Nature Utopia	**0.93 (0.89-0.96, p<0.001)**	1.01 (0.98-1.04, p=0.515)	**0.88 (0.81-0.94, p=0.001)**	0.91 (0.73-1.13, p=0.393)
**Diabetes Mellitus**	Nature Deficient / Nature Light	Reference			
	Nature Adequate	**0.93 (0.89-0.98, p=0.005)**	**1.05 (1.02-1.09, p=0.004)**	1.02 (0.97-1.07, p=0.473)	**0.83 (0.70-0.99, p=0.035)**
	Nature Rich	**0.93 (0.89-0.98, p=0.003)**	**1.04 (1.00-1.07, p=0.025)**	0.97 (0.91-1.02, p=0.217)	0.88 (0.72-1.08, p=0.230)
	Nature Utopia	**0.90 (0.86-0.94, p<0.001)**	0.97 (0.94-1.00, p=0.089)	**0.88 (0.79-0.98, p=0.018)**	0.95 (0.65-1.39, p=0.799)
**Dyslipidemia**	Nature Deficient / Nature Light	Reference			

	Nature Adequate	**1.07 (1.03-1.11, p<0.001)**	**1.03 (1.00-1.06, p=0.026)**	1.02 (0.98-1.05, p=0.353)	0.95 (0.87-1.04, p=0.256)
	Nature Rich	**1.04 (1.01-1.08, p=0.024)**	**1.04 (1.01-1.06, p=0.005)**	0.99 (0.95-1.02, p=0.434)	**0.82 (0.73-0.91, p<0.001)**
	Nature Utopia	1.01 (0.98-1.05, p=0.536)	**1.05 (1.03-1.08, p<0.001)**	1.00 (0.93-1.07, p=0.904)	0.86 (0.69-1.06, p=0.155)
**Obesity**	Nature Deficient / Nature Light	Reference			
	Nature Adequate	1.00 (0.97-1.04, p=0.982)	**1.06 (1.03-1.08, p<0.001)**	1.03 (1.00-1.07, p=0.082)	0.98 (0.88-1.09, p=0.717)
	Nature Rich	0.98 (0.94-1.01, p=0.134)	**1.07 (1.05-1.10, p<0.001)**	0.95 (0.92-0.99, p=0.018)	**0.79 (0.69-0.91, p=0.001)**
	Nature Utopia	**0.95 (0.92-0.98, p=0.004)**	**1.05 (1.03-1.08, p<0.001)**	**0.87 (0.81-0.94, p<0.001)**	0.83 (0.63-1.10, p=0.194)
**Smoking**	Nature Deficient / Nature Light	Reference			

	Nature Adequate	**0.95 (0.92-0.99, p=0.025)**	**1.05 (1.02-1.08, p<0.001)**	1.01 (0.97-1.05, p=0.804)	0.95 (0.85-1.06, p=0.322)
	Nature Rich	**0.94 (0.90-0.97, p=0.001)**	**1.05 (1.02-1.08, p<0.001)**	**0.93 (0.89-0.97, p=0.002)**	**0.87 (0.76-1.00, p=0.045)**
	Nature Utopia	**0.91 (0.88-0.95, p<0.001)**	0.99 (0.97-1.02, p=0.665)	**0.89 (0.82-0.96, p=0.003)**	1.04 (0.81-1.33, p=0.758)

aOR: Adjusted odds ratio; ASCVD: Atherosclerotic cardiovascular disease; CI: Confidence intervals; CV: Cardiovascular.

Model 4: Adjusted for age, sex, race/ethnicity, Area Deprivation Index (ADI), and WalkScore.

Table S17. Multivariable logistic regression for cardiovascular risk factors and diseases across the various NatureScore groups, stratified by neighborhood walkability using subgroup analysis of various age groups (n=1,077,181).

		**Multivariable Analysis after Stratification using Model 4**			
**Outcome**	**Exposure Categories**	**Car-Dependent (All Errands) (0-19)**	**Car-Dependent (Most Errands) (20-39)**	**Somewhat Walkable (40-59)**	**Very Walkable / Walker's Paradise (60-100)**
		**aOR (95% CI, p-value)**			
		**Age group 18-39 years (n=314,776)**			
**Any CV risk factor**	Nature Deficient / Nature Light	Reference			
	Nature Adequate	1.15 (0.85-1.54, p=0.360)	0.91 (0.72-1.14, p=0.403)	0.94 (0.74-1.20, p=0.630)	0.93 (0.56-1.54, p=0.777)
	Nature Rich	1.12 (0.85-1.49, p=0.415)	0.83 (0.67-1.03, p=0.084)	1.03 (0.79-1.33, p=0.834)	1.18 (0.59-2.35, p=0.640)
	Nature Utopia	1.07 (0.81-1.41, p=0.619)	**0.78 (0.62-0.98, p=0.033)**	**0.67 (0.46-0.99, p=0.043)**	1.28 (0.39-4.24, p=0.683)
**Hypertension**	Nature Deficient / Nature Light	Reference			

	Nature Adequate	1.02 (0.86-1.22, p=0.815)	0.99 (0.86-1.13, p=0.848)	0.98 (0.85-1.14, p=0.825)	1.06 (0.76-1.49, p=0.713)
	Nature Rich	1.08 (0.91-1.27, p=0.391)	0.90 (0.80-1.02, p=0.107)	0.92 (0.78-1.07, p=0.264)	0.94 (0.63-1.41, p=0.764)
	Nature Utopia	1.09 (0.93-1.29, p=0.294)	0.88 (0.78-1.01, p=0.064)	**0.74 (0.58-0.96, p=0.020)**	0.71 (0.38-1.33, p=0.268)
**Diabetes Mellitus**	Nature Deficient / Nature Light	Reference			
	Nature Adequate	1.00 (0.90-0.90, p=0.957)	0.94 (0.87-1.01, p=0.089)	0.98 (0.89-1.09, p=0.745)	1.26 (0.96-1.67, p=0.101)
	Nature Rich	0.99 (0.89-1.10, p=0.869)	**0.92 (0.86-0.98, p=0.014)**	**0.88 (0.79-0.98, p=0.021)**	1.27 (0.91-1.77, p=0.164)
	Nature Utopia	0.94 (0.85-1.05, p=0.275)	**0.91 (0.85-0.98, p=0.013)**	**0.75 (0.62-0.91, p=0.004)**	1.33 (0.75-2.36, p=0.335)
**Dyslipidemia**	Nature Deficient / Nature Light	Reference			

	Nature Adequate	1.00 (0.89-1.11, p=0.932)	1.03 (0.96-1.11, p=0.450)	1.04 (0.94-1.15, p=0.419)	1.14 (0.89-1.46, p=0.314)
	Nature Rich	1.03 (0.93-1.14, p=0.564)	**1.08 (1.01-1.16, p=0.023)**	1.07 (0.96-1.18, p=0.215)	1.01 (0.75-1.36, p=0.953)
	Nature Utopia	0.97 (0.87-1.07, p=0.501)	1.06 (0.99-1.14, p=0.093)	0.97 (0.81-1.15, p=0.709)	0.95 (0.57-1.59, p=0.852)
**Obesity**	Nature Deficient / Nature Light	Reference			
	Nature Adequate	0.98 (0.87-1.10, p=0.682)	1.00 (0.92-1.08, p=0.961)	1.06 (0.95-1.18, p=0.319)	**0.71 (0.52-0.98, p=0.040)**
	Nature Rich	1.01 (0.90-1.12, p=0.898)	1.00 (0.93-1.07, p=0.930)	1.04 (0.92-1.17, p=0.555)	0.95 (0.66-1.37, p=0.777)
	Nature Utopia	0.97 (0.87-1.08, p=0.545)	1.00 (0.92-1.08, p=0.950)	1.05 (0.86-1.29, p=0.629)	0.87 (0.45-1.67, p=0.673)
**Smoking**	Nature Deficient / Nature Light	Reference			

	Nature Adequate	0.99 (0.88-1.11, p=0.810)	0.93 (0.86-1.01, p=0.068)	0.94 (0.85-1.04, p=0.197)	1.05 (0.82-1.35, p=0.700)
	Nature Rich	0.97 (0.87-1.08, p=0.590)	**0.93 (0.86-1.00, p=0.049)**	**0.85 (0.76-0.94, p=0.002)**	1.00 (0.74-1.35, p=0.976)
	Nature Utopia	0.96 (0.86-1.06, p=0.411)	0.93 (0.86-1.00, p=0.053)	**0.78 (0.65-0.93, p=0.006)**	0.97 (0.58-1.62, p=0.913)
		**Age group 40-64 years (n=450,291)**			
**Any CV risk factor**	Nature Deficient / Nature Light	Reference			
	Nature Adequate	0.99 (0.95-1.03, p=0.614)	0.99 (0.95-1.03, p=0.614)	0.99 (0.95-1.03, p=0.614)	0.99 (0.95-1.03, p=0.614)
	Nature Rich	**0.96 (0.92-0.99, p=0.017)**	**0.96 (0.92-0.99, p=0.017)**	**0.96 (0.92-0.99, p=0.017)**	**0.96 (0.92-0.99, p=0.017)**
	Nature Utopia	**0.92 (0.89-0.95, p<0.001)**	**0.92 (0.89-0.95, p<0.001)**	**0.92 (0.89-0.95, p<0.001)**	**0.92 (0.89-0.95, p<0.001)**
**Hypertension**	Nature Deficient / Nature Light	Reference			

	Nature Adequate	0.97 (0.93-1.01, p=0.133)	**1.05 (1.02-1.08, p=0.001)**	**0.93 (0.90-0.97, p=0.001)**	**0.89 (0.81-0.99, p=0.029)**
	Nature Rich	**0.95 (0.92-0.99, p=0.010)**	**1.05 (1.02-1.08, p<0.001)**	**0.93 (0.89-0.97, p<0.001)**	**0.82 (0.73-0.93, p=0.002)**
	Nature Utopia	**0.93 (0.89-0.96, p<0.001)**	1.01 (0.98-1.04, p=0.515)	**0.88 (0.81-0.94, p=0.001)**	0.91 (0.73-1.13, p=0.393)
**Diabetes Mellitus**	Nature Deficient / Nature Light	Reference			
	Nature Adequate	**0.93 (0.89-0.98, p=0.005)**	**1.05 (1.02-1.09, p=0.004)**	1.02 (0.97-1.07, p=0.473)	**0.83 (0.70-0.99, p=0.035)**
	Nature Rich	**0.93 (0.89-0.98, p=0.003)**	**1.04 (1.00-1.07, p=0.025)**	0.97 (0.91-1.02, p=0.217)	0.88 (0.72-1.08, p=0.230)
	Nature Utopia	**0.90 (0.86-0.94, p<0.001)**	0.97 (0.94-1.00, p=0.089)	**0.88 (0.79-0.98, p=0.018)**	0.95 (0.65-1.39, p=0.799)
**Dyslipidemia**	Nature Deficient / Nature Light	Reference			

	Nature Adequate	**1.07 (1.03-1.11, p<0.001)**	**1.03 (1.00-1.06, p=0.026)**	1.02 (0.98-1.05, p=0.353)	0.95 (0.87-1.04, p=0.256)
	Nature Rich	**1.04 (1.01-1.08, p=0.024)**	**1.04 (1.01-1.06, p=0.005)**	0.99 (0.95-1.02, p=0.434)	**0.82 (0.73-0.91, p<0.001)**
	Nature Utopia	1.01 (0.98-1.05, p=0.536)	**1.05 (1.03-1.08, p<0.001)**	1.00 (0.93-1.07, p=0.904)	0.86 (0.69-1.06, p=0.155)
**Obesity**	Nature Deficient / Nature Light	Reference			
	Nature Adequate	1.00 (0.97-1.04, p=0.982)	**1.06 (1.03-1.08, p<0.001)**	1.03 (1.00-1.07, p=0.082)	0.98 (0.88-1.09, p=0.717)
	Nature Rich	0.98 (0.94-1.01, p=0.134)	**1.07 (1.05-1.10, p<0.001)**	0.95 (0.92-0.99, p=0.018)	**0.79 (0.69-0.91, p=0.001)**
	Nature Utopia	**0.95 (0.92-0.98, p=0.004)**	**1.05 (1.03-1.08, p<0.001)**	**0.87 (0.81-0.94, p<0.001)**	0.83 (0.63-1.10, p=0.194)
**Smoking**	Nature Deficient / Nature Light	Reference			

	Nature Adequate	**0.95 (0.92-0.99, p=0.025)**	**1.05 (1.02-1.08, p<0.001)**	1.01 (0.97-1.05, p=0.804)	0.95 (0.85-1.06, p=0.322)
	Nature Rich	**0.94 (0.90-0.97, p=0.001)**	**1.05 (1.02-1.08, p<0.001)**	**0.93 (0.89-0.97, p=0.002)**	**0.87 (0.76-1.00, p=0.045)**
	Nature Utopia	**0.91 (0.88-0.95, p<0.001)**	0.99 (0.97-1.02, p=0.665)	**0.89 (0.82-0.96, p=0.003)**	1.04 (0.81-1.33, p=0.758)
**Age group 65-79 years (n=243,760)**					
**Any CV risk factor**	Nature Deficient / Nature Light	Reference			
	Nature Adequate	1.15 (0.85-1.54, p=0.360)	0.91 (0.72-1.14, p=0.403)	0.94 (0.74-1.20, p=0.630)	0.93 (0.56-1.54, p=0.777)
	Nature Rich	1.12 (0.85-1.49, p=0.415)	0.83 (0.67-1.03, p=0.084)	1.03 (0.79-1.33, p=0.834)	1.18 (0.59-2.35, p=0.640)
	Nature Utopia	1.07 (0.81-1.41, p=0.619)	**0.78 (0.62-0.98, p=0.033)**	**0.67 (0.46-0.99, p=0.043)**	1.28 (0.39-4.24, p=0.683)
**Hypertension**	Nature Deficient / Nature Light	Reference			

	Nature Adequate	1.02 (0.86-1.22, p=0.815)	0.99 (0.86-1.13, p=0.848)	0.98 (0.85-1.14, p=0.825)	1.06 (0.76-1.49, p=0.713)
	Nature Rich	1.08 (0.91-1.27, p=0.391)	0.90 (0.80-1.02, p=0.107)	0.92 (0.78-1.07, p=0.264)	0.94 (0.63-1.41, p=0.764)
	Nature Utopia	1.09 (0.93-1.29, p=0.294)	0.88 (0.78-1.01, p=0.064)	**0.74 (0.58-0.96, p=0.020)**	0.71 (0.38-1.33, p=0.268)
**Diabetes Mellitus**	Nature Deficient / Nature Light	Reference			
	Nature Adequate	1.00 (0.90-0.90, p=0.957)	0.94 (0.87-1.01, p=0.089)	0.98 (0.89-1.09, p=0.745)	1.26 (0.96-1.67, p=0.101)
	Nature Rich	0.99 (0.89-1.10, p=0.869)	**0.92 (0.86-0.98, p=0.014)**	**0.88 (0.79-0.98, p=0.021)**	1.27 (0.91-1.77, p=0.164)
	Nature Utopia	0.94 (0.85-1.05, p=0.275)	**0.91 (0.85-0.98, p=0.013)**	**0.75 (0.62-0.91, p=0.004)**	1.33 (0.75-2.36, p=0.335)
**Dyslipidemia**	Nature Deficient / Nature Light	Reference			

	Nature Adequate	1.00 (0.89-1.11, p=0.932)	1.03 (0.96-1.11, p=0.450)	1.04 (0.94-1.15, p=0.419)	1.14 (0.89-1.46, p=0.314)
	Nature Rich	1.03 (0.93-1.14, p=0.564)	**1.08 (1.01-1.16, p=0.023)**	1.07 (0.96-1.18, p=0.215)	1.01 (0.75-1.36, p=0.953)
	Nature Utopia	0.97 (0.87-1.07, p=0.501)	1.06 (0.99-1.14, p=0.093)	0.97 (0.81-1.15, p=0.709)	0.95 (0.57-1.59, p=0.852)
**Obesity**	Nature Deficient / Nature Light	Reference			
	Nature Adequate	0.98 (0.87-1.10, p=0.682)	1.00 (0.92-1.08, p=0.961)	1.06 (0.95-1.18, p=0.319)	**0.71 (0.52-0.98, p=0.040)**
	Nature Rich	1.01 (0.90-1.12, p=0.898)	1.00 (0.93-1.07, p=0.930)	1.04 (0.92-1.17, p=0.555)	0.95 (0.66-1.37, p=0.777)
	Nature Utopia	0.97 (0.87-1.08, p=0.545)	1.00 (0.92-1.08, p=0.950)	1.05 (0.86-1.29, p=0.629)	0.87 (0.45-1.67, p=0.673)
**Smoking**	Nature Deficient / Nature Light	Reference			

	Nature Adequate	0.99 (0.88-1.11, p=0.810)	0.93 (0.86-1.01, p=0.068)	0.94 (0.85-1.04, p=0.197)	1.05 (0.82-1.35, p=0.700)
	Nature Rich	0.97 (0.87-1.08, p=0.590)	**0.93 (0.86-1.00, p=0.049)**	**0.85 (0.76-0.94, p=0.002)**	1.00 (0.74-1.35, p=0.976)
	Nature Utopia	0.96 (0.86-1.06, p=0.411)	0.93 (0.86-1.00, p=0.053)	**0.78 (0.65-0.93, p=0.006)**	0.97 (0.58-1.62, p=0.913)
		**Age group 80+ years (n=68,354)**			
**Any CV risk factor**	Nature Deficient / Nature Light	Reference			
	Nature Adequate	0.99 (0.95-1.03, p=0.614)	0.99 (0.95-1.03, p=0.614)	0.99 (0.95-1.03, p=0.614)	0.99 (0.95-1.03, p=0.614)
	Nature Rich	**0.96 (0.92-0.99, p=0.017)**	**0.96 (0.92-0.99, p=0.017)**	**0.96 (0.92-0.99, p=0.017)**	**0.96 (0.92-0.99, p=0.017)**
	Nature Utopia	**0.92 (0.89-0.95, p<0.001)**	**0.92 (0.89-0.95, p<0.001)**	**0.92 (0.89-0.95, p<0.001)**	**0.92 (0.89-0.95, p<0.001)**
**Hypertension**	Nature Deficient / Nature Light	Reference			

	Nature Adequate	0.97 (0.93-1.01, p=0.133)	**1.05 (1.02-1.08, p=0.001)**	**0.93 (0.90-0.97, p=0.001)**	**0.89 (0.81-0.99, p=0.029)**
	Nature Rich	**0.95 (0.92-0.99, p=0.010)**	**1.05 (1.02-1.08, p<0.001)**	**0.93 (0.89-0.97, p<0.001)**	**0.82 (0.73-0.93, p=0.002)**
	Nature Utopia	**0.93 (0.89-0.96, p<0.001)**	1.01 (0.98-1.04, p=0.515)	**0.88 (0.81-0.94, p=0.001)**	0.91 (0.73-1.13, p=0.393)
**Diabetes Mellitus**	Nature Deficient / Nature Light	Reference			
	Nature Adequate	**0.93 (0.89-0.98, p=0.005)**	**1.05 (1.02-1.09, p=0.004)**	1.02 (0.97-1.07, p=0.473)	**0.83 (0.70-0.99, p=0.035)**
	Nature Rich	**0.93 (0.89-0.98, p=0.003)**	**1.04 (1.00-1.07, p=0.025)**	0.97 (0.91-1.02, p=0.217)	0.88 (0.72-1.08, p=0.230)
	Nature Utopia	**0.90 (0.86-0.94, p<0.001)**	0.97 (0.94-1.00, p=0.089)	**0.88 (0.79-0.98, p=0.018)**	0.95 (0.65-1.39, p=0.799)
**Dyslipidemia**	Nature Deficient / Nature Light	Reference			

	Nature Adequate	**1.07 (1.03-1.11, p<0.001)**	**1.03 (1.00-1.06, p=0.026)**	1.02 (0.98-1.05, p=0.353)	0.95 (0.87-1.04, p=0.256)
	Nature Rich	**1.04 (1.01-1.08, p=0.024)**	**1.04 (1.01-1.06, p=0.005)**	0.99 (0.95-1.02, p=0.434)	**0.82 (0.73-0.91, p<0.001)**
	Nature Utopia	1.01 (0.98-1.05, p=0.536)	**1.05 (1.03-1.08, p<0.001)**	1.00 (0.93-1.07, p=0.904)	0.86 (0.69-1.06, p=0.155)
**Obesity**	Nature Deficient / Nature Light	Reference			
	Nature Adequate	1.00 (0.97-1.04, p=0.982)	**1.06 (1.03-1.08, p<0.001)**	1.03 (1.00-1.07, p=0.082)	0.98 (0.88-1.09, p=0.717)
	Nature Rich	0.98 (0.94-1.01, p=0.134)	**1.07 (1.05-1.10, p<0.001)**	0.95 (0.92-0.99, p=0.018)	**0.79 (0.69-0.91, p=0.001)**
	Nature Utopia	**0.95 (0.92-0.98, p=0.004)**	**1.05 (1.03-1.08, p<0.001)**	**0.87 (0.81-0.94, p<0.001)**	0.83 (0.63-1.10, p=0.194)
**Smoking**	Nature Deficient / Nature Light	Reference			

	Nature Adequate	**0.95 (0.92-0.99, p=0.025)**	**1.05 (1.02-1.08, p<0.001)**	1.01 (0.97-1.05, p=0.804)	0.95 (0.85-1.06, p=0.322)
	Nature Rich	**0.94 (0.90-0.97, p=0.001)**	**1.05 (1.02-1.08, p<0.001)**	**0.93 (0.89-0.97, p=0.002)**	**0.87 (0.76-1.00, p=0.045)**
	Nature Utopia	**0.91 (0.88-0.95, p<0.001)**	0.99 (0.97-1.02, p=0.665)	**0.89 (0.82-0.96, p=0.003)**	1.04 (0.81-1.33, p=0.758)

aOR: Adjusted odds ratio; ASCVD: Atherosclerotic cardiovascular disease; CI: Confidence intervals; CV: Cardiovascular.

Model 4: Adjusted for age, sex, race/ethnicity, Area Deprivation Index (ADI), and WalkScore.
